# Nest substrate reflects incubation style in extant archosaurs with implications for dinosaur nesting habits

**DOI:** 10.1038/s41598-018-21386-x

**Published:** 2018-03-15

**Authors:** Kohei Tanaka, Darla K. Zelenitsky, François Therrien, Yoshitsugu Kobayashi

**Affiliations:** 1 Research Fellow of Japan Society for the Promotion of Science, Nagoya University Museum, Furocho, Chikusa-Ku, Nagoya, Aichi 464-8601 Japan; 2 Department of Geoscience, University of Calgary, 2500 University Drive, Northwest, Calgary, Alberta T2N 1N4 Canada; 3 Royal Tyrrell Museum of Palaeontology, PO Box 7500, Drumheller, Alberta T0J 0Y0 Canada; 4 Hokkaido University Museum, Kita 10, Nishi 8, Kita-Ku, Sapporo, Hokkaido, 060-0801 Japan

## Abstract

Dinosaurs thrived and reproduced in various regions worldwide, including the Arctic. In order to understand their nesting in diverse or extreme environments, the relationships between nests, nesting environments, and incubation methods in extant archosaurs were investigated. Statistical analyses reveal that species of extant covered nesters (i.e., crocodylians and megapodes) preferentially select specific sediments/substrates as a function of their nesting style and incubation heat sources. Relationships between dinosaur eggs and the sediments in which they occur reveal that hadrosaurs and some sauropods (i.e., megaloolithid eggs) built organic-rich mound nests that relied on microbial decay for incubation, whereas other sauropods (i.e., faveoloolithid eggs) built sandy in-filled hole nests that relied on solar or potentially geothermal heat for incubation. Paleogeographic distribution of mound nests and sandy in-filled hole nests in dinosaurs reveals these nest types produced sufficient incubation heat to be successful up to mid latitudes (≤47°), 10° higher than covered nesters today. However, only mound nesting and likely brooding could have produced sufficient incubation heat for nesting above the polar circle (>66°). As a result, differences in nesting styles may have placed restrictions on the reproduction of dinosaurs and their dispersal at high latitudes.

## Introduction

Dinosaurs nested in a variety of regions and environments as indicated by the geologic and paleogeographic occurrences of their egg remains worldwide^1–3^. With egg remains recovered from low (18°) to high (up to 77°) paleolatitudes^2,4^, this range indicates that dinosaurs successfully incubated their eggs in various climates, presumably using brooding behaviors or various styles of nests like living archosaurs (i.e., crocodylians and birds). Brooding birds today are able to nest in a wide latitudinal range because the parent provides constant, adequate heat to incubate the eggs. In contrast, covered nesters (i.e., crocodylians and megapode birds), for which the eggs are enclosed in nest materials during incubation, are presumably restricted to nesting at low latitudes (≤37°) because, in part, they rely on heat sources like solar radiation for incubation^5,6^.

Covered nesting appears to have been a common behavior among non-avian dinosaurs, particularly for taxa outside of maniraptoran theropods^7–11^. These dinosaurs likely used either in-filled holes in sand or mound nests consisting of organic nest materials to incubate their eggs as in living crocodylians and megapodes. However, little is known about nest style/composition and incubation heat source of dinosaurs taxa because these are generally not preserved. Presumably, nest composition and style would have played an important role in the generation and/or transfer of heat to the eggs as they do in living archosaurs^5,6,12^. It also stands to reason that there is a relationship between nest composition/style (e.g., plant materials, sands, and soil^13–16^) and the nature of heat source (e.g., solar radiation, microbial respiration, and geothermal activity^5,6,12^), although this remains untested statistically among living archosaur species. In this study, we test several relationships between nest composition/style and incubation heat sources in extant archosaurs in order to infer the nesting style and incubation heat source of particular dinosaur taxa. Paleolatitudinal occurrences of nesting sites among non-avian dinosaurs are examined in order to infer how dinosaurs nested at various latitudes, including in the Arctic region.

## Statistical Tests and Results

### Extant covered nesters

Among living crocodylians and megapode birds, we test whether nest temperature is significantly higher than ambient temperature at the nesting site in order to determine if certain types of covered nests could theoretically have been used in cooler climates or high paleolatitudes by dinosaurs. Nests were subdivided into two categories based on the source of incubation heat used: (1) nests that derive incubation heat mainly from decomposition of organic matter due to microbial respiration (including termite nests^12^), and (2) nests that derive incubation heat primarily from solar radiation.

**Figure 1:**
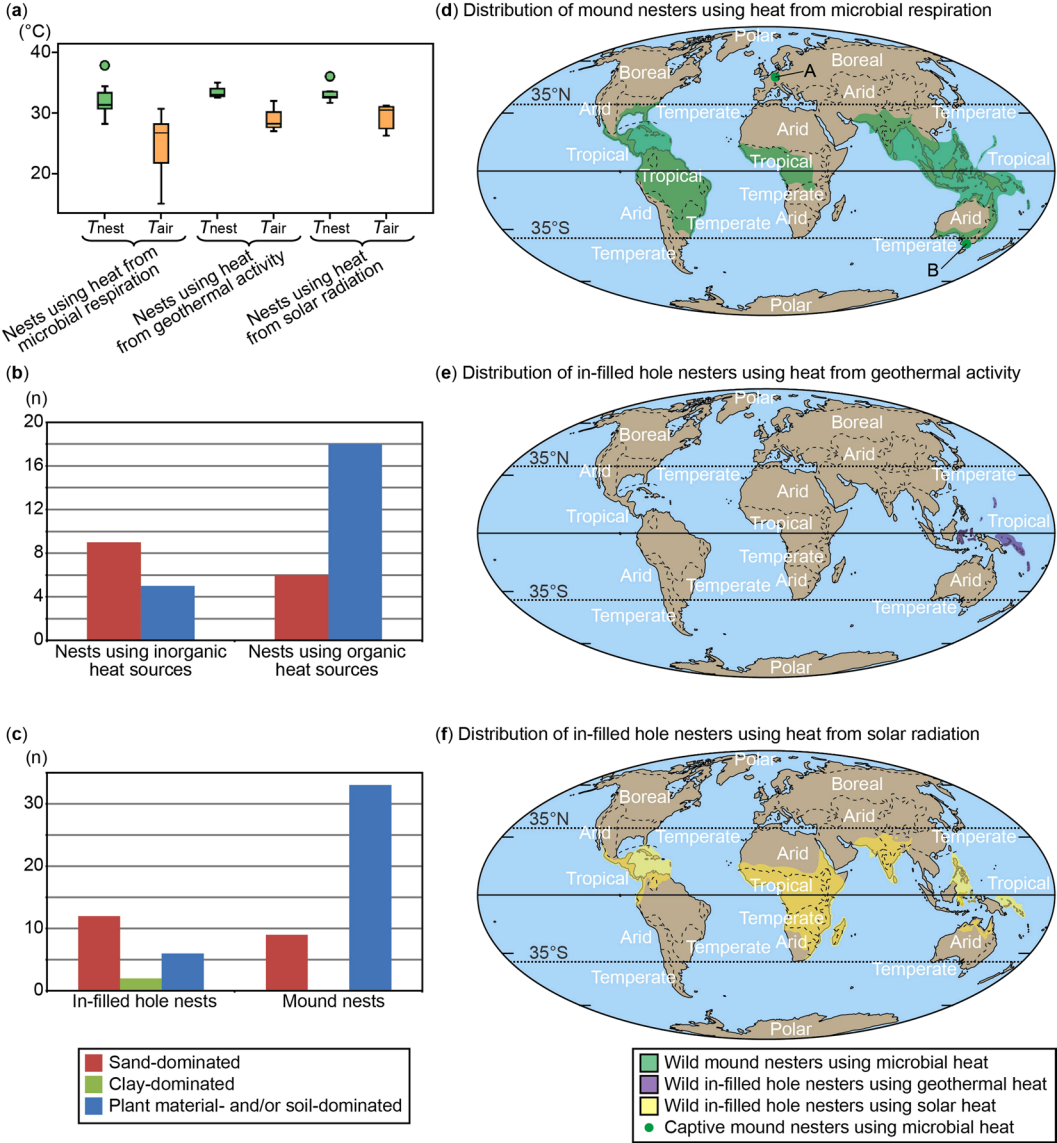
Nesting parameters of living covered nesters. (**a**) Comparison of nest (T_nest_) and ambient air temperature (T_air_) among nests using different incubation heat sources, showing that temperature in all types of nests is higher than ambient temperature. (**b**) Comparison of nest materials/substrates between nests that use inorganic or organic heat sources, showing that use of nest material/substrate is different between these two incubation heat sources. (**c**) Comparison of nest material/substrate composition between mound and in-filled hole nests, showing that use of nest materials/substrates is different between these two nest structures. (**d**) Geographic distributions of mound nesters that use heat from microbial respiration. A and B represent Frankfurt and Melbourne, respectively, where successful nesting of captive mound-nesting megapodes has been documented. (**e**) Geographic distributions of in-filled hole nesters that use heat from geothermal activity. (**f**) Geographic distributions of in-filled hole nesters that use heat from solar radiation. The maps were created with Adobe Illustrator CS5 based on the original map provided by Global Paleogeography and Tectonics in Deep Time ©2016 Colorado Plateau Geosystems Inc.

Paired-samples T-tests revealed that nest temperature derived from both microbial respiration and solar radiation is significantly different from ambient air temperature (t = 7.26, d.f. = 16, p < 0.01 for nests that derive heat from microbial respiration and t = 3.93, d.f. = 8, p < 0.01 for nests that derive heat from solar radiation). With the exception of a single case (i.e., *Alligator mississippiensis*^13^), the temperature of covered nests is always higher than ambient air temperature among living archosaurs. For nests that use incubation heat derived from solar radiation, the difference between nest and air temperature ranged from 0.98 °C to 6.75 °C (mean 3.93 °C, standard deviation 2.29), whereas the temperature difference was considerably higher (mean 7.26 °C) and more variable (from −2.50 °C to 22.20 °C, standard deviation 6.35) for nests that derive incubation heat from microbial respiration (Fig. 1a).

Because both the type of material/substrate used to build the nest and nest style play a significant role in the generation and/or transfer of heat to the eggs in covered nesters^5,6,12^, we test whether a relationship exists between the type of nest materials/substrates (i.e., sand, clay, and plant material and/or soil), the incubation heat source (i.e., heat from microbial respiration, geothermal activity, or solar radiation) and the nest structure (i.e., mounds or in-filled holes) in living archosaurs. A Fisher’s exact test reveals that a relationship exists between the type of nest materials/substrates and the source of incubation heat among living archosaurs (p < 0.05, Fig. 1b). In nests where heat from solar radiation and geothermal activities (herein referred to as inorganic heat sources) are used for incubation, sand is a more frequently-used nesting substrate (n = 9) than plant materials and/or soils (n = 5). In contrast, nests that predominantly use heat from microbial respiration (referred to as organic heat sources in this study) for incubation more commonly consist of plant materials and/or soils (n = 18) than sand (n = 6). Nests made of clay were not observed among the extant species considered in this test.

Interestingly, mound nests are shown to be built from different nest materials/substrates than in-filled hole nests (Fisher’s exact test: p < 0.01, Fig. 1c). The majority of mound nesters use plant materials and/or soils (n = 33), whereas sand (n = 9) is much less common and clay is not used. However, in-filled hole nesters commonly lay eggs within sand (n = 12), whereas clay (n = 2) or plant materials (n = 6) are infrequently used.

### Non-avian dinosaurs

Lithologic data for nests, eggs, and eggshells of various dinosaur egg taxa (i.e., ootaxa) were statistically compared to determine if some dinosaurs favored certain types of sediments to build covered nests. If a relationship between these variables exists, then it is possible to infer the nest structures and incubation heat sources used by dinosaurs from the lithology of their nests. We compiled and tested two datasets: (1) a first dataset in which the egg clutches are presumably *in-situ*, and (2) a second dataset in which isolated eggs and eggshells are potentially not *in-situ* (assumed *ex-situ*).

Dinosaur egg/eggshell remains occur in both fine- (i.e., mudstone and siltstone) and coarse-grained sediments (i.e., sandstone and conglomerate) in sub-equal proportions (1:0.88 and 1:0.80 in the *in-situ* and *ex-situ* datasets, respectively) (Table 1), although relationships are found with some taxa and regions in the *in-situ* dataset (Table 2). For example, clutches of megaloolithid sauropod eggs tend to occur in fine-grained pedogenic deposits in Romania and Spain, but in coarse-grained pedogenic deposits in India. Clutches of oviraptorosaurs (elongatoolithid eggs) and troodontids (prismatoolithid eggs) are mainly recovered from fine-grained deposits in China (for oviraptorosaurs) and in North America (for troodontids), but from coarse-grained deposits in Mongolia. In contrast, faveoloolithid sauropod clutches are found mainly in coarse-grained deposits regardless of the geographic region of the egg sites.

**Table 1:** Results of one-way χ^2^-tests for the *in-situ* and *ex-situ* datasets of dinosaur ootaxa.

Ootaxa	n for fine-grained	n for coarse-grained	Total n	Expected n for fine-grained	Expected n for coarse-grained	χ^2^ value	d.f.	p
***In-situ*** **dataset**								
Dendroolithidae	5	5	10	5.313	4.687	0.039	1	0.843
Elongatoolithidae	14	13	27	14.344	12.656	0.018	1	0.894
Faveoloolithidae	4	32	36	19.125	16.875	25.518	1	<0.001
Megaloolithidae	44	22	66	35.063	30.937	4.860	1	0.027
Prismatoolithidae	9	11	20	10.625	9.375	0.530	1	0.530
Spheroolithidae	10	3	13	NA	NA	NA	NA	NA
Other ootaxa	16	4	20	10.625	9.375	5.801	1	0.0016
Total	102	90	192	NA	NA	NA	NA	NA
***Ex-situ*** **dataset**								
Dendroolithidae	1	3	4	NA	NA	NA	NA	NA
Elongatoolithidae	15	14	29	16.096	12.904	0.168	1	0.682
Faveoloolithidae	7	9	16	8.880	7.120	0.894	1	0.344
Megaloolithidae	25	29	54	29.971	24.029	1.853	1	0.173
Prismatoolithidae	10	10	20	11.100	8.900	0.245	1	0.621
Spheroolithidae	15	9	24	13.321	10.679	0.476	1	0.490
Other ootaxa	43	19	62	34.411	27.589	4.818	1	0.028
Total	116	93	209	NA	NA	NA	NA	NA

Spheroolithidae from the *in-situ* dataset and Dendroolithidae from the *ex-situ* dataset were not tested due to the small sample sizes. Abbreviations: d.f., degree of freedom; n, sample size; NA, not applicable; p, probability for χ^2^-tests.

**Table 2:** Number of occurrences of major dinosaur clutches among countries.

Ootaxa	Country	Fine-grained deposits	Coarse-grained deposits
Elongatoolithidae	China	10 (0)	2 (0)
	Mongolia	1 (0)	8 (1)
	Other countries	3 (0)	3 (2)
Faveoloolithidae	Argentina	0 (0)	8 (7)
	South Korea	3 (1)	21 (0)
	Other countries	1 (0)	3 (0)
Megaloolithidae	Argentina	5 (2)	5 (2)
	France	5 (0)	0 (0)
	India	0 (0)	10 (10)
	Romania	12 (9)	1 (1)
	Spain	22 (14)	6 (3)
Prismatoolithidae	Mongolia	0 (0)	8 (1)
	Canada and USA	6 (4)	1 (0)
	Other countries	3 (0)	2 (0)

Parentheses indicate number of pedogenic deposits in each case.

**Figure 2:**
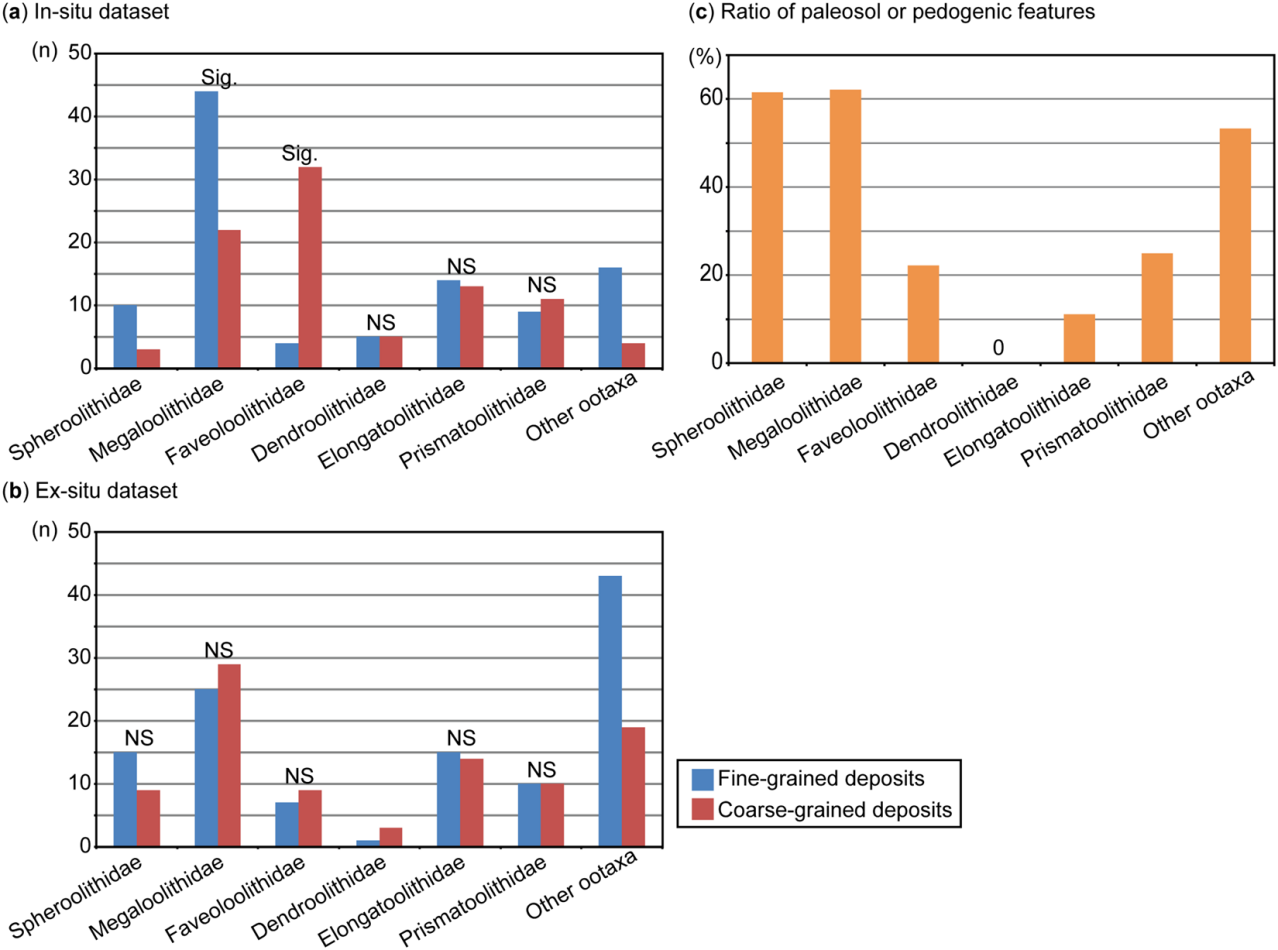
Comparison of sediment types among dinosaur oofamilies. (**a**) Comparison of lithology from the *in-situ* clutch dataset, showing that *in-situ* clutches of Spheroolithidae, Megaloolithidae, and Faveoloolithidae tend to be associated with particular type of sediments whereas other oofamilies show no preferential association with lithology. (**b**) Comparison of lithology from the *ex-situ* dataset, showing that oofamilies in the *ex-situ* dataset are not significantly associated with specific lithologies. (**c**) Relative abundance of paleosol or pedogenic features found in dinosaur clutches, showing that clutches of Spheroolithidae and Megaloolithidae are strongly associated with paleosols, whereas other groups are not. Abbreviations: sig., significant; NS, not significant at 0.05 level.

One-way χ^2^-tests for the *in-situ* dataset reveal that certain types of dinosaur egg clutches preferentially occur in certain lithologies (Table 1, Fig. 2a). Faveoloolithid clutches are significantly associated with coarse-grained deposits, whereas megaloolithid clutches are preferentially associated with fine-grained deposits. Although hadrosaur clutches (Spheroolithidae) were not subjected to a statistical test due to the small sample size, they tend to be associated with fine-grained deposits (n = 10) rather than coarse-grained deposits (n = 3). In contrast, there was no significant lithological association observed for clutches of Dendroolithidae (non-avian theropods such as therizinosaurs), Elongatoolithidae (oviraptorosaurs) and Prismatoolithidae (troodontids). For the *ex-situ* dataset, one-way χ^2^-tests revealed no evident relationships between lithology and particular taxa/ootaxa (Table 1, Fig. 2b), presumably reflecting their transportation and re-deposition away from the original sediments.

Evidence of pedogenic development is common in clutch horizons of hadrosaurs and of sauropods that lay megaloolithid eggs, with a frequency of 61.5% and 62.1%, respectively (Fig. 2c). However, pedogenic features were much less common in the sediments associated with other taxa/ootaxa, such as dendroolithid theropods, faveoloolithid sauropods, oviraptorosaurs, and troodontids, where the frequency of pedogenic features was 25% or less.

## Discussion

Our study demonstrates that a statistically-significant relationship exists between nest types, nest substrates, and incubation heat sources among extant covered-nesting archosaurs (i.e., crocodylians and megapode birds), results that can be applied to infer aspects of nesting in extinct non-avian dinosaurs. Among living archosaurs, we have statistically shown that species that build mound nests preferentially use plant materials or soil^13,15^, whereas those that utilize in-filled hole nests preferentially build in sand^17–20^. Furthermore, egg clutches incubated by organic heat sources (microbial respiration) are usually laid in mound nests made of plant materials and/or soil, whereas those incubated by inorganic heat sources (solar radiation, geothermal activity) are commonly laid in nests built in sand, probably due to the high thermal conductivity of this substrate^21,22^. Although covered nest temperature tends to be higher than ambient air temperature regardless of heat source, nest temperature derived from microbial respiration is not correlated with ambient air temperature^23–25^, whereas nest temperature derived from solar radiation is^16,26^. Microbial respiration produces nest temperatures that are much higher than ambient air temperature (mean difference 7.26 °C, with cases reaching 15–44 °C above ambient temperature^27,28^), whereas solar radiation produces nest temperatures only moderately higher than ambient air temperature (mean difference 3.93 °C). Thus, mound nests that rely on organic heat sources (i.e., microbial respiration) for incubation would be suited for animals nesting in cooler or more northerly regions as they can provide adequate incubation heat even when ambient air temperature is much lower than nest temperature. Although extant mound nesters are limited to within 37° of the equator, captive mound-nesting megapodes are able to naturally incubate at much higher latitudes and cooler temperatures than the bird’s native habitats^29,30^ (Fig. 1d). Although few living species rely, even partly, on geothermal heat for incubation^15,31,32^ (Fig. 1e), this heat source theoretically could also be used to incubate eggs at high latitudes or in cool environments if available. In contrast, living archosaurs that rely mainly on solar radiation to incubate their eggs can only nest where the ambient air temperature is close to the required nest/incubation temperature (Fig. 1a). As such, these animals are restricted to nest primarily to within 30° of the equator (Fig. 1f), presumably because required solar radiation in these regions is sufficiently high to maintain nest temperature ≥28 °C, the minimum temperature required for successful incubation in living archosaurs^33^. Thus, unlike microbial respiration and potentially geothermal activity, solar radiation is likely not a reliable source of incubation heat in cooler or northerly environments.

**Figure 3:**
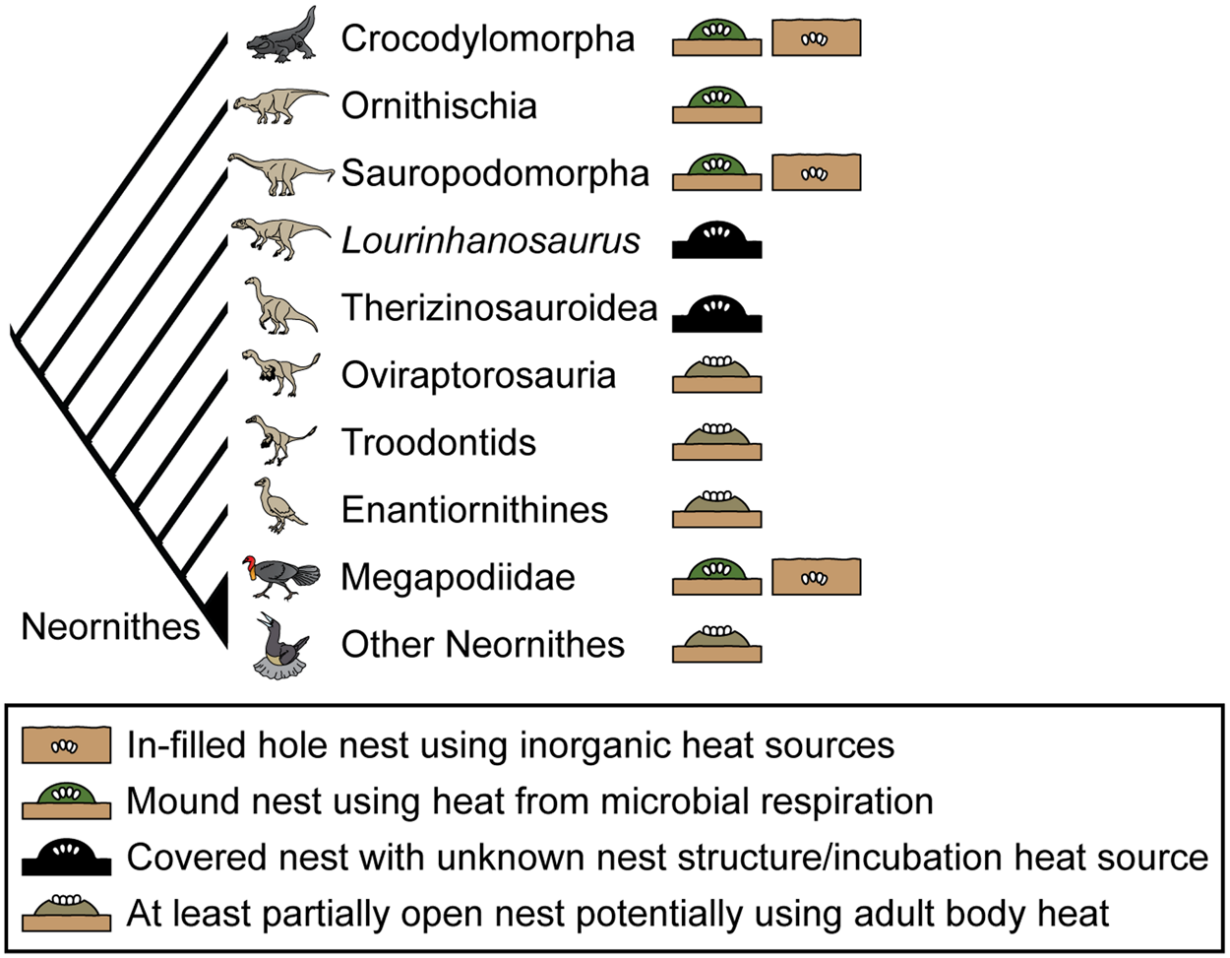
Inferred nest structures and incubation heat sources for dinosaurs. Covered and open nest types were inferred from eggshell porosity and/or taphonomy^9,11^. Illustrations of archosaurs, drawn by one of the authors (KT), are modified from Tanaka *et al*.^11^.

Although some dinosaurs, namely hadrosaurs and sauropods, are shown to have built covered nests to incubate their eggs^7,9–11,34^, the nature of their nests and incubation heat sources has remained uncertain. Our analyses reveal that these covered nesters favored certain styles or substrates for their nests, which correspond to particular incubation heat sources (Fig. 3). Eggs of hadrosaurs (i.e., spheroolithids) and some sauropods (i.e., megaloolithids) are preferentially associated with fine-grained pedogenic sediments, indicating these animals built mound nests from organic-rich materials to incubate their eggs via microbial respiration. In contrast, eggs of other sauropods (i.e., faveoloolithid) are shown to be preferentially associated with non-pedogenic, coarse-grained sediments (particularly sandstone), suggesting that they were incubated in in-filled hole nests excavated in sand, like some crocodylian and megapode species (see species lists in Greer^35^ and Booth & Jones^6^). As such, these sauropods presumably relied on inorganic heat sources, like solar radiation or possibly geothermal heat^36^, to incubate their eggs.

Some maniraptoran theropods (i.e., oviraptorosaurs and troodontids), in contrast to hadrosaurs and sauropods, are known to have built open nests where the eggs were at least partly exposed during incubation^11,37^, which has been related to brood-like behaviors in these animals^38–40^. Our study shows that their eggs are not associated preferentially with a specific lithology, indicating that these dinosaurs did not select particular substrates for nesting. This result is expected for animals for which incubation heat is provided by the adult and nest substrate is not crucial for generation or absorption of heat as in covered nesters. With direct body-egg contact, oviraptorosaurs and troodontids were likely freed from the restrictions imposed by external incubation heat sources and could be less selective in terms of nesting substrates.

**Figure 4:**
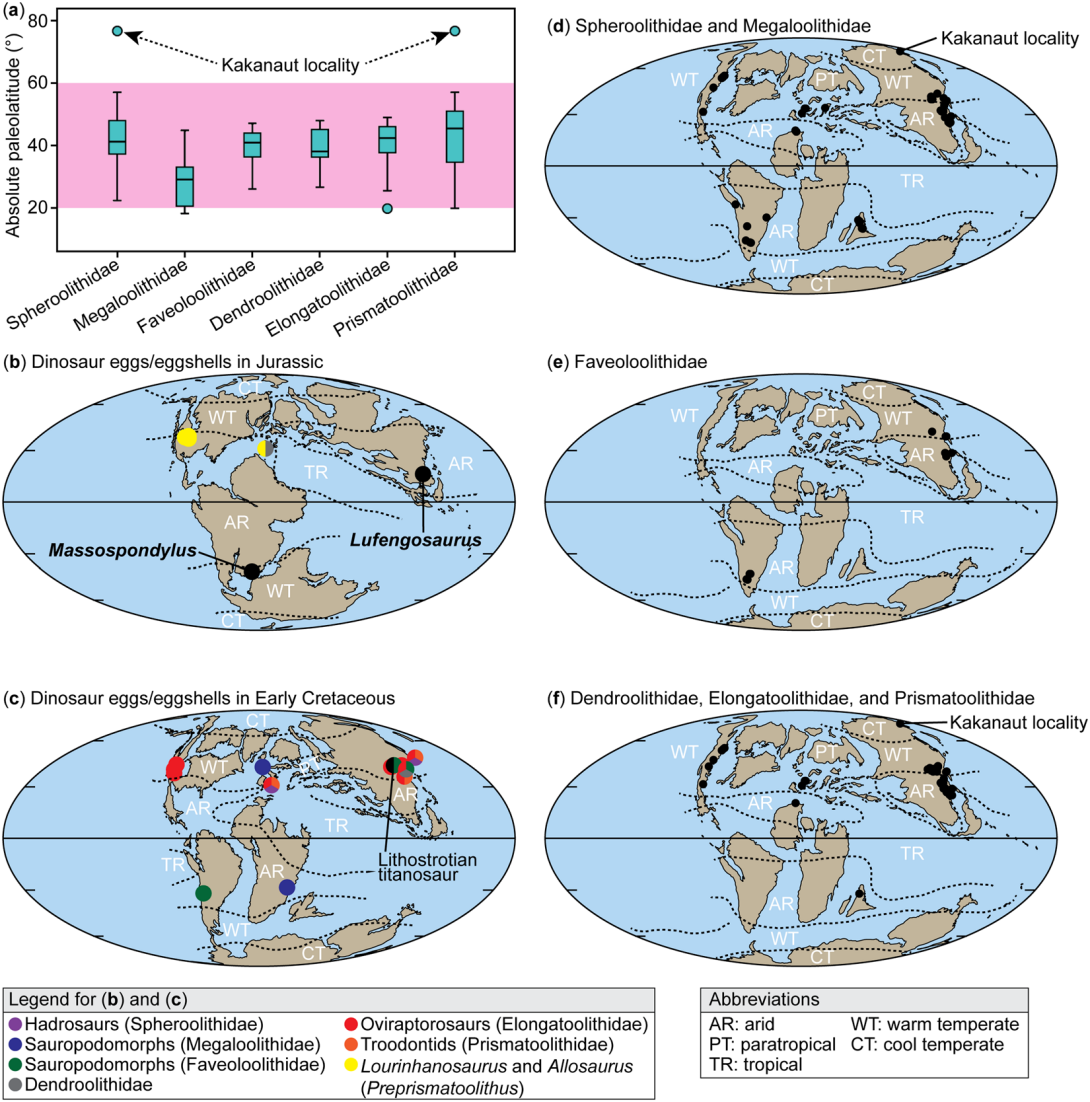
Paleogeographic distribution of major dinosaur oofamilies. (**a**) Comparison of paleolatitude of dinosaur egg/eggshell localities, showing that most localities range within 20° and 60° (highlighted in pink) whereas spheroolithid and prismatoolithid eggshells are also known from higher latitudes (Kakanaut locality at 76.7°N). (**b**) Paleogeographic distribution of dinosaur egg/eggshell localities of Jurassic age. (**c**) Paleogeographic distribution of dinosaur egg/eggshell localities of Early Cretaceous. (**d**) Paleogeographic distribution of Spheroolithidae and Megaloolithidae in the Late Cretaceous (dots). (**e**) Paleogeographic distribution of Faveoloolithidae during the Late Cretaceous (dots). (**f**) Paleogeographic distribution of Dendroolithidae, Elongatoolithidae, and Prismatoolithidae during the Late Cretaceous (dots). The maps were created with Adobe Illustrator CS5 based on the original maps provided by Global Paleogeography and Tectonics in Deep Time ©2016 Colorado Plateau Geosystems Inc.

The various nesting strategies here recognized in dinosaurs help explain patterns in the paleolatitudinal distribution of different taxa (Table 3; Fig. 4). Among covered nesting dinosaurs, sauropod nest sites (megaloolithid and faveoloolithid eggs) are distributed up to 47° from the equator, whereas hadrosaur egg remains (spheroolithids) have the widest latitudinal range, up to 76.7°N (Kakanaut locality^2^, Table 3; Fig. 4a). The paleogeographic distribution of covered nesting dinosaurs far exceeds the range of living covered-nesting archosaurs (generally restricted to ≤37° from the equator^15,41^). This poleward spread of covered nesters is undoubtedly related to warmer paleoclimatic conditions as there is widespread evidence that the Late Cretaceous climate, the time interval from which most dinosaur eggs are known, was much warmer, with a lower latitudinal temperature gradient than today^42,43^. The Campanian Auca Mahuevo nesting site, for example, which has produced numerous sauropod clutches (megaloolithids) in Patagonia (42.3°S of paleolatitude) is estimated to have had an average ambient temperature of 40 °C for a warm month^44^. This warmer climate appears to have allowed both mound nesting and in-filled hole nesting dinosaurs to successfully reproduce at least 10° latitude higher than modern archosaurs of similar nesting habits. Situated farther north at 76.7°N, Late Cretaceous dinosaurs nesting in the Arctic faced a much cooler paleoclimate, with mean annual and summer temperatures of 10 °C and 19 °C, respectively^45,46^. Nevertheless, these climatic conditions are similar to those of Frankfurt (50°N, data derived from http://www.dwd.de/EN/Home/home_node.html), where mound-nesting megapodes can breed successfully in captivity^30^ despite being situated 13° north of the natural range of covered nesters (Fig. 1d). Thus, successful nesting by hadrosaurs in the Cretaceous Arctic was likely related to their mound-nesting behaviors, as such nests were probably the only style of covered nest capable of generating sufficient incubation heat. In contrast, in-filled hole nests were presumably not a viable option at such extreme latitudes due to their reliance on solar radiation to maintain sufficient incubation heat. Among open nesters, troodontids have the highest latitudinal occurrence of 76.7°N, which as probable brooders likely allowed them, and other maniraptorans, to nest in more diverse climates/habitats, including the Arctic, because they had little or no reliance on external incubation heat sources (Fig. 4d,f). These conclusions suggest that only mound-nesting or brooding dinosaurs could have reproduced in the Arctic. As such, one can predict that if sauropod eggs are ever discovered in the Arctic, they should be megaloolithid eggs rather than faveoloolithid eggs as only the former were generally incubated in mound nests.

**Table 3:** Ranges of absolute paleolatitudes in major dinosaur ootaxa.

Ootaxa	n	Range of absolute paleolatitudes
Dendroolithidae	18	26.70° to 48.00°
Elongatoolithidae	54	19.85° to 49.00°
Faveoloolithidae	13	26.10° to 47.10°
Megaloolithidae	28	18.30° to 44.85°
Prismatoolithidae	33	19.95° to 76.70°
Spheroolithidae	45	22.45° to 76.70°

Abbreviation: n, sample size.

Ultimately, nesting strategies may have played an underappreciated role in the paleobiogeographic distribution of dinosaurs. Although geographic barriers, climate, and biological filters have been used to explain why some dinosaur clades succeeded or failed to spread to various regions^47^, aspects of their reproduction are rarely considered^46^. Recognition that certain nesting styles have intrinsic requirements that restrict the latitudinal distribution of archosaurs reveals that reproduction may have affected the dispersal of some dinosaurs to high latitudes during the Mesozoic. For example, high-latitude intercontinental land bridges may have prevented the dispersal of in-filled hole nesters while the movement of mound nesters and brooders was unimpeded. Various dinosaur taxa (e.g., hadrosaurs, ankylosaurs, ceratopsians, pachycephalosaurs, tyrannosaurs, troodontids, and dromaeosaurs) are known to have inhabited Alaska and Siberia^2,48,49^, dispersing between western North America and Asia via a high-latitude land bridge during the Late Cretaceous. Their subsistence in the Arctic may have been possible, in part, because these dinosaurs either built mound nests or brooded, while other taxa (e.g., faveoloolithid sauropods) failed to disperse at high latitude because their nesting style required more solar radiation than available for egg incubation. Although factors influencing dispersal events are complex, the ability to assess the nesting strategies for dinosaurs in a paleogeographic context may help evaluate possible scenarios to explain the global distribution of dinosaurs.

## Methods

### Datasets and statistical tests for extant taxa

Nest and ambient air temperature for Crocodylia and Megapodiidae was obtained from the literature and the difference between these two variables was calculated for each species/population (see Supplementary Table S1). Most previous studies measured mean nest temperature at the center of the nest or around the egg chamber during incubation. Ambient air temperature represents either measurements taken around the nests or averages between minimum and maximum air temperature during nesting season. Ambient air temperature at crocodylian and megapode nesting sites are fairly stable; fluctuations of the temperature during nesting season and the mean daily temperature are less than 10 °C in most cases. Heat produced by embryonic metabolism may contribute to nest temperature^12,50–52^; however, metabolic heat was not considered in this study because it is difficult to distinguish metabolic heat from nest temperature. Embryonic metabolic heat can potentially raise egg temperature 1–3 °C in crocodylian and megapode nests^51–53^, and could be a small contribution to nest temperature^12,54^. Paired-samples T-tests were used to compare nest and air temperature for nests which derive incubation heat from microbial respiration and from solar radiation. All statistical analyses were conducted with IBM SPSS Statistics v. 22.0.0 (IBM SPSS Inc.) at an α level of 0.05.

In order to determine if the type of nesting material/substrate differs between nests of different incubation heat sources and structure (i.e., mound and in-filled hole nests), data on nest material/substrate and nest structure in 42 species of Crocodylia and Megapodiidae were gleaned from the literature (see Supplementary Table S2) and compared using Fisher’s exact test. Incubation heat sources were classified as predominantly from either: (1) inorganic heat source (i.e., solar radiation and geothermal activities), or (2) organic heat source (i.e., microbial respiration associated with decaying plant material or termite nest with potential supplemental contribution from inorganic heat sources). Nest material/substrate was classified as either: (1) sand-dominated, (2) clay-dominated, or (3) plant material- (e.g., leaves, twigs/sticks, roots, tree stump^13^) and/or soil-dominated. Nest structure was classified as either a mound (pile of nest materials on the burrowed or flat ground) or in-filled hole nest (underground hole).

### Datasets and statistical tests for fossil taxa/ootaxa

In order to test whether dinosaur nests are associated with particular types of sediments, lithologic data of dinosaur egg specimens were obtained from the literature, supplemented with specimen observations at the Royal Tyrrell Museum of Palaeontology in Drumheller, Canada (TMP 1994.179.01, TMP 1996.086.01, TMP 2008.075.51, TMP 1989.079.53, TMP 1988.079.36, and TMP1997.63.1). For this study, two datasets were compiled based on the quality of fossil preservation: (1) clutches and nesting grounds in which the eggs are presumably *in-situ* (see Supplementary Table S3); and (2) isolated eggs and eggshells potentially not *in-situ* (assumedly *ex-situ*) (see Supplementary Table S4). Both datasets consist of various dinosaur ootaxa, including eggs that can be accurately referred to specific dinosaur taxa (see Supplementary Table S5). In this study, oofamily Spheroolithidae is referred to as hadrosaur eggs, Megaloolithidae and Faveoloolithidae as sauropodomorph eggs, Elongatoolithidae as oviraptorosaur eggs, and Prismatoolithidae as troodontid (and possibly other non-oviraptorosaur maniraptoran) eggs. *Preprismatoolithus* eggs were excluded from the oofamily Prismatoolithidae because they belong to *Allosaurus* and *Lourinhanosaurus*, which are not maniraptorans^55,56^. In the *in-situ* dataset, multiple clutches from a single ootaxon discovered within a single horizon (including colonial grounds) were considered as one occurrence. Clutches of a single ootaxon discovered from multiple horizons within a single locality were considered as multiple occurrences by counting the number of clutch horizons. Lithologic data were classified into either fine-grained (i.e., siltstone and mudstone, including marls) or coarse-grained (i.e., sandstone and conglomerate). One-way χ^2^-tests were conducted in order to test whether a particular type of sediments (i.e., fine-grained and coarse-grained) is associated with each ootaxon, following the method of Lyson & Longrich^57^.

Evidence of paleosol and/or pedogenic features (e.g., root traces, rhizoliths, caliche nodules, slickensides, bioturbation, and pedotubules^58^) in clutch horizons was also recorded for the *in-situ* dataset. Although paleosols that developed after burial of the nests^59–61^ were not incorporated into the dataset, it was often difficult to determine in pedogenic development occurred before or after nesting.

Additionally, the paleogeographic distribution of egg/eggshell remains was investigated for major dinosaur oofamilies (see Supplementary Table S6). Information about fossil egg/eggshell localities from around the world was gathered from the literature, and their approximate paleolatitudes were calculated using the website paleolatitude.org^62^. If paleolatitudes could not be calculated with this website, the Paleobiology Database (https://paleobiodb.org/#/) was used.

### Data availability

All data generated or analyzed during this study are included in this published article (and its Supplementary Information file).

## Electronic supplementary material

Supplementary Information
