# Supplementary material for: Nest substrate reflects incubation style in extant archosaurs with implications for dinosaur nesting habits
Source: Sci Rep. 2018 Mar 15;8:3170. doi: 10.1038/s41598-018-21386-x (PMC5854591; doi:10.1038/s41598-018-21386-x)
Supplement: Supplementary file 1 — Supplementary Information [file 41598_2018_21386_MOESM1_ESM.pdf]

Nest substrate reflects incubation style in extant archosaurs with implications for dinosaur nesting habits

Kohei Tanaka, Darla K. Zelenitsky, François Therrien, and Yoshitsugu Kobayashi

Supplementary Table S1. Mean nest temperature (T_{nest}, °C), mean ambient air temperature (T_{air}, °C), and the difference between T_{nest} and T_{air} (°C) of living covered nesters.

Note that * indicates mean values were calculated in this study using the data provided by the papers.

Incubation heat source	Taxon	T_{nest}	T_{air}	Difference	References
Microbial respiration	*Aepypodius arfakianus*	31.100	21.800	9.300	Sinclair (2001)
	Aepypodius sp.	37.800	15.600	22.200	Frith (1956)
	Alectura lathami	33.300	15.100	18.200	Jones (1988)
		32.950	17.800	15.150	Seymour and Bradford (1992)
	Leipoa ocellata	32.626*	27.185*	5.441	Lewis (1940)
	Megapodius decollatus	31.100	22.100	9.000	Sinclair (2001)
	Megapodius freycinet	34.400	26.750	7.650	Lincoln (1974)
		33.500*	26.750*	6.750	Stuebing and Johan (1986)
	Megapodius laperouse	31.300	28.500	2.800	Wiles and Conry (2001)
	Megapodius nicobariensis	32.440	28.170	4.270	Sivakumar and Sankaran (2003)
	Talegalla jobiensis	30.600	21.800	8.800	Sinclair (2001)

Incubation heat source	Taxon	T_{nest}	T_{air}	Difference	References
	Alligator mississippiensis	34.034	28.020	6.014	McIlhenny (1934)
		28.200	30.700	-2.500	Joanen (1969)
		29.833	28.267	1.566	Chabreck (1973)
	Caiman c. crocodilus	29.900*	29.571*	0.329	Staton and Dixon (1977)
	Crocodylus cataphractus	30.700*	25.500*	5.200	Waitkuwait (1985)
	Melanosuchus niger	31.200	27.900	3.300	Villamarln-Jurado and Suarez (2007)
Geothermal heat	*Macrocephalon maleo*	35.000*	28.250*	6.750	MacKinnon (1978)
	Megapodius eremita	33.000	32.000	1.000	Roper (1983)
	Megapodius pritchardii	32.500*	27.000*	5.500	Goth and Vogel (1996–1997)
Solar radiation	*Eulipoa wallacei* (dry season values)	32.600	27.442*	5.158	Heij et al. (1997)
	Eulipoa wallacei (rainy season values)	32.500	26.285*	6.215	Heij et al. (1997)
	Macrocephalon maleo	36.000	31.000	5.000	MacKinnon (1978)
	Megapodius laperouse	33.500*	30.2500*	3.250	Glass et al. (1988)
	Crocodylus acutus	31.670	30.689	0.981	Charruau (2012)
	Crocodylus niloticus	32.725*	31.213*	1.512	Modha (1967)

Supplementary Table S2. Nest type, nest material, and heat source of incubation in living covered nesters.

Abbreviations: C, clay; H, in-filled hole nest; I, inorganic heat source; M, mound nest; O, organic heat source (with possibly some inroganic heat); PS, plant material and/or soil; Sa, sand.

Order	Species	Nest type	Nest material	Heat source	References
Galliformes	*Aepypodius arfakianus*	M	PS	O	Sinclair (2001)
	Aepypodius bruijni	M	PS	O	Mauro (2005)
	Alectura lathami	M	PS	O	Le Souef (1899); Frith (1956)
	Eulipoa wallacei	H	Sa	I	Ripley (1960); Rand and Gilliard (1967); Dekker et al. (1995); Heij et al. (1997); Baker (1999); Baker and Dekker (2000)
	Leipoa ocellata	M	Sa	O	Le Souef (1899); Frith (1956; 1959)
	Macrocephalon maleo	H	Sa	I	MacKinnon (1978); Dekker (1988); Argeloo and Dekker (1996)
		H	PS	I	Dekker (1988)
	Megapodius bernsteinii	M	PS	I	Indrawan et al. (1998)
		M	Sa	I	Indrawan et al. (1998)
	Megapodius cumingii	M	Sa	O	Jones et al. (1995)
	Megapodius decollatus	M	PS	O	Sinclair (2001)
	Megapodius eremita	H	Sa	I	Roper (1983)
	Megapodius forstenii	M	PS		Jones et al. (1995)
		M	Sa		Jones et al. (1995)

Order	Species	Nest type	Nest material	Heat source	References
		M	PS		Stresemann (1914)
	Megapodius freycinet	H	Sa	O	MacKinnon (1978)
		M	Sa	I	Lincoln (1974)
		M	PS		Ripley (1960)
		M	PS	O	Lincoln (1974)
	Megapodius laperous laperous	H	Sa		Falanruw (1975)
	Megapodius l. senex	H	PS	I	Glass et al. (1988)
		M	PS		Stinson and Glass (1992)
		M	Sa	O	Wiles and Conry (2001)
	Megapodius layardi	H	PS	O	Bowen (1996)
		H	PS	I	Bregulla (1992)
	Megapodius nicobariensis	M	Sa	O	Sankaran (1995); Sivakumar and Sankaran (2003)
	Megapodius pritchardii	H	Sa	I	Friedlander (1899)
		H	PS	I	Kellers (1931); Weir (1973)
	Megapodius reinwardt	M	PS	O	Le Souef (1899); Crome and Brown (1979)
		M	Sa	O	Crome and Brown (1979)
	Talegalla cuvieri	M	PS		Gilliard and LeCroy (1970)
	Talegalla fuscirostris	M	PS		Jones et al. (1995)

Order	Species	Nest type	Nest material	Heat source	References
	Talegalla jobiensis	M	PS	O	Sinclair (2001)
Crocodylia	*Alligator mississippiensis*	M	PS	O	Joanen (1969); Chabreck (1975); Metzen (1977); Goodwin and Marion (1978)
	Alligator sinensis	M	PS	O	Thorbjarnarson et al. (2001)
	Caiman crocodilus	M	PS	O	Ouboter and Nanhoe (1987); Allsteadt (1994)
		M	PS	O	Staton and Dixon (1977)
	Caiman latirostris	M	PS		Montini et al. (2006)
	Caiman yacare	M	PS		Crawshaw and Schaller (1980); Cintra (1988)
	Crocodylus acutus	H	Sa	I	Platt and Thorbjarnarson (2000); Charruau (2012)
		M	PS		Ogden (1978); Mazzotti (1989)
		M	Sa		Ogden (1978); Lutz and Dunbar-Vooper (1984); Kushlan and Mazzotti (1989); Mazzotti (1989)
	Crocodylus cataphractus	M	PS	O	Waitkuwait (1985)
	Crocodylus intermedius	H	Sa		Medem (1981); Bolhm (1982); Thorbjarnarson and Hernandez (1993); Seijas (1998); Espinosa-Blanco et al. (2013)
		M	PS		Medem (1981)
	Crocodylus johnstoni	H	Sa	I	Webb et al. (1983a); Somaweera and Shine (2013)
	Crocodylus mindorensis	M	PS		van Weerd et al. (2006); van Weerd (2010)

Order	Species	Nest type	Nest material	Heat source	References
	Crocodylus moreletii	M	PS	O	Platt et al. (2008); Lopez-Luna et al. (2015)
	Crocodylus niloticus	H	C		Pooley (1969)
		H	Sa	I	Cott (1961); Modha (1967); Pooley (1969); Kofron (1989); Swanepoel et al. (2000)
	Crocodylus novaeguineae	M	PS	O	Neil (1946); Hall and Johnson (1987)
	Crocodylus palustris	H	C		Whitaker and Whitaker (1976)
		H	Sa		Whitaker and Whitaker (1976); Rao and Gurjwar (2013)
	Crocodylus porosus	H	PS		Whitaker and Whitaker (1976)
		M	PS	O	Webb et al. (1977; 1983b)
	Crocodylus siamensis	M	PS		Bezuijen et al. (2013)
	Gavialis gangeticus	H	Sa		Bustard (1980); Rao (1988); Nair and Katdare (2013); Rao et al. (2013); Rao and Gurjwar (2013)
	Melanosuchus niger	M	PS	O	Herron et al. (1990); Villamarln-Jurado and Suarez (2007)
	Osteolaemus tetraspis	M	PS		Riley and Huchzermeyer (1999)
	Paleosuchus palpebrosus	M	PS		Medem (1958; 1971)
	Paleosuchus trigonatus	M	PS	O	Magnusson et al. (1985); Rivas et al. (2001)
	Tomistoma schlegelii	M	PS		Staniewicz (2011)

Supplementary Table S3. Lithology of in-situ dinosaur eggs (clutches and nesting grounds).

*Indicates pedogenic feature(s) is identified. Abbreviation: NA, not available; TMP, Royal Tyrrell Museum of Palaeontology, Drumheller, Canada.

Ootaxon/taxon	Material	Lithology	Formation	Locality	References
Cairanoolithidae					
Cairanoolithus dughii	Clutch(es)	Fine	NA	Commune de Rousset, near Cairanne, Aix-en-Provence, France	Williams et al. (1984)
Cairanoolithus cf. *roussetensis*	Clutch(es)	Fine	Tremp	Coll de Nargo, Lleida, Catalonia, Spain	Sellés et al. (2013)
Dendroolithidae (/Phaceloolithidae)					
D. cf. *dendriticus*	Clutch(es)	Fine	Chichengshan	Shuangtang Tiantai Basin, Zhejiang, China	Fang et al. (1998; 2000)
D. fengguangcunensis	Clutch(es)	Coarse	Dongyuan	Heyuan Basin, Guangdong, China	Fang et al. (2005)
D. sp.	Clutch(es)	Coarse	Nemegt	Khaichin Ula, Gobi, Mongolia	Suzuki and Watabe (2000b)
Phaceloolithus hunanensis	Clutch(es)	Fine	Fenshuiao	Western Dongting Basin, Hunan, China	Zeng and Zhang (1979)
Dendroolithidae indet.	Clutch(es)	Coarse	?Djadokhta	Toosgot area, Abdrant Nuru, Gobi, Mongolia	Watabe (2004)

Ootaxon/taxon	Material	Lithology	Formation	Locality	References
Dendroolithidae indet.	Clutch(es)	Coarse	Shiwa	Shiwa Bay, Whaseong, Gyeonggi, South Korea	Lee (2003)
Dendroolithidae indet.	Clutch(es) (Location 8-1G)	Fine	Sihwa	Hanyom, Sihwa Basin, South Korea	Kim et al. (2009)
Dendroolithidae indet.	Clutch(es) (Locations 8-2 and 8-3)	Coarse	Sihwa	Hanyom, Sihwa Basin, South Korea	Kim et al. (2009)
Dendroolithidae indet.	Clutch(es)	Fine	Baynshire	Bayn Shire, Gobi, Mongolia	Watabe et al. (2010)
Torvosaurus	Clutch(es) with embryonic bones	Fine	Lourinha	Walen, Porto das Barcas, Portugal	Araujo et al. (2013)
Dictyoolithidae					
Dictyoolithus gongzhulingensis	Clutch(es)	Fine	Quantou	Gongzhuling area, Jilin, China	Wang et al. (2006)
D. hongpoensis	Clutch(es)	Coarse	Chichengshan	Lishui Basin, Zhejiang, China	Jin et al. (2010)
Protodictyoolithus neixiangensis	Clutch(es)	Fine	Goseong	Yeondo, Dosanmyeon, Tongyeong City, Gyeongsang, South Korea	Kim et al. (2011)
P. neixiangensis	Clutch(es)	Fine	Goseong	Southwest coast	Kim et al. (2011)

Ootaxon/taxon	Material	Lithology	Formation	Locality	References
				Pyeongri, Dosanmyeon, Tongyeong City, Gyeongsang, South Korea	
Elongatoolithidae					
Ellipsoolithus khedaensis	Clutch(es)	Coarse*	Lameta	Lavariya Muwada, Kheda, Gujarat, India	Mohabey (1998)
Elongatoolithus andrewsi	Clutch(es)	Fine	Yuanpu and Pingling	Tangmienling, Shuikou, Nanxiong Basin, Guangdong, China	Young (1965); Zhao (1975)
E. elongatus	Clutch(es)	Fine	Yuanpu and Pingling	Nanxiong Basin, Guangdong, China	Young (1965); Zhao (1975)
E. elongatus	Clutch(es)	Coarse	NA	Baiyuan, Pingxiang, Jiangxi, China	Wu and Peng (2003)
E. magnus	Clutch(es)	Fine	Fenshuiao	Western Dongting Basin, Hunan, China	Zeng and Zhang (1979)
Macroelongatoolithus carlylei	A pair of eggs	Fine	Wayan	Bonneville County, Idaho, USA	Krumenacker et al. (2016)
M. goseongensis	Clutch(es)	Fine	Goseong	Ddabakseom, Pyeongri, Dosanmyeon, Tongyeong City,	Kim et al. (2011)

Ootaxon/taxon	Material	Lithology	Formation	Locality	References
				Gyeongsang, South Korea	
M. xixiaensis	Clutch(es)	Fine	Gaogou	Xixia Basin, Henan, China	Wang and Zhou (1995)
M. xixiaensis	Clutch(es)	Fine	NA	Zhouzhi Hill, Zhaoying Village, Yangcheng Town, Xixia Basin, Henan, China	Li et al. (1995)
M. xixiaensis	Clutch(es)	Fine	Chichengshan	Shuangtang, Tiantai Basin, Zhejiang, China	Wang et al. (2010)
M. xixiaensis	Clutch(es)	Fine	Gyeongsang Basin	Located on the island of Aphae-do in Shinan-gun, Jeollanam-do, southwestern Korea	Huh et al. (2014)
Macroolithus rugustus	Clutch(es)	Fine	Yuanpu and Pingling	Lashuyuan, Wuching, Nanxiong Basin, Guangdong, China	Young (1965); Zhao (1975)
M. rugustus?	Clutch(es)	Coarse*	Nemegt	Khaychin-Ula, Gobi, Mongolia	Sochava (1969); Mikhailov (1994b)
M. yaotunensis	Clutch(es)	Fine	Yuanpu and Pingling	Yaotun, Nanxiong Basin, Guangdong,	Young (1965); Zhao (1975)

Ootaxon/taxon	Material	Lithology	Formation	Locality	References
				China	
M. yaotunensis	Clutch(es)	Fine	Yuanpu and Pingling	Yaotun, Nanxiong Basin, Guangdong, China	Young (1965); Zhao (1975)
Nanhsiungoolithus chuetienensis	Clutch(es)	Fine	Yuanpu and Pingling	Nanxiong, Nanxiong Basin, Guangdong, China	Young (1965); Zhao (1975)
Trachoolithus sp.	Clutch(es)	Coarse*	Lameta	Lavariay Muwada, Gujarat, India	Mohabey (2000; 2001)
Elongatoolithidae indet.	Clutch(es)	Coarse	Shiwa	Shiwa Bay, Whaseong, Gyeonggi, South Korea	Lee (2003)
Elongatoolithidae indet.	Clutch(es)	Coarse	Djadokhta	Bayn Dzak, Gobi, Mongolia	Watabe et al. (2010)
Elongatoolithidae indet.	Clutch(es)	Coarse	Baynshire	Bayn Shire, Gobi, Mongolia	Watabe et al. (2010)
Elongatoolithidae indet.	Clutch(es)	Coarse	NA	Tel Ulan Chaltsai (Mogoin Daatsyn Khuduk), Mongolia	Watabe et al. (2010)
E. frustrabilis	Clutch(es)	Coarse	Djadokhta	Shabarakh Usu MNG, Mongolia	Brown and Schlaikjer (1940); van Straelen (1925)

Ootaxon/taxon	Material	Lithology	Formation	Locality	References
E. frustrabilis?	Clutch(es) with embryonic bones	Coarse	Djadokhta	Xanadu, Ukhaa Tolgod, south-central Mongolia	Norell et al. (1994; 2001)
Citipati osmolskae	Clutch(es) with adult skeleton	Coarse	Djadokhta	Ankylosaur Flats, Ukhaa Tolgod, Mongolia	Clark et al. (1999); Grellet-Tinner et al. (2006)
Nemegtomaia barsboldi	Clutch(es) with adult skeleton	Coarse	Baruungoyot	Northern Sayr of the Nemegt locality, Mongolia	Fanti et al. (2012)
Oviraptor philoceratops (cf. *Machairasaurus*)	Clutch(es) with adult skeleton	Coarse	Djadokhta	North Canyon, Bayan Mandahu, Inner Mongolia, China	Dong and Currie (1996)
Elongatoolithidae indet.	Clutch(es) with embryonic bones	Fine	Nemegt	Bugin-Tsav, Gobi, Mongolia	Weishampel et al. (2008)
Faveoloolithidae					
Faveoloolithus ningxiaensis	Clutch(es)	Coarse	NA	Bayinwulashan cahanaobao, Alxa Left Banner (Inner Mongolia), Ningxia, China	Zhao and Ding (1976); Zhang (2010)
F. ningxiaensis	Clutch(es)	Coarse	Baruungoyot	Ologoy-Ulan-Tsav, Gobi, Mongolia	Sochava (1969); Mikhailov (1994a)

Ootaxon/taxon	Material	Lithology	Formation	Locality	References
F. sp.	Clutch(es)	Fine*	Seonso	Bosung County, Chullanam-do, South Korea	Huh and Zelenitsky (2002); Kim et al. (2009); Paik et al. (2004)
Parafaveoloolithus macroporus	Clutch(es)	Fine	Laijia	Laijia Village, Tiantai, Zhejiang, China	Zhang (2010)
Faveoloolithidae indet.	Clutch(es)	Coarse	Shiwa	Shiwa Bay, Whaseong, Gyeonggi, South Korea	Lee (2003)
Faveoloolithidae indet.	Clutch(es) (Locations 1-1 I5, 1-2, and 1-4)	Fine	Sihwa	Hanyom, Sihwa Basin, South Korea	Kim et al. (2009)
Faveoloolithidae indet.	Clutch(es) (Location 3-1 Islets between)	Fine	Sihwa	Hanyom, Sihwa Basin, South Korea	Kim et al. (2009)
Faveoloolithidae indet.	Clutch(es) (Locations 3-2 HH and JH, 3-3, and 3-4)	Coarse	Sihwa	Hanyom, Sihwa Basin, South Korea	Kim et al. (2009)
Faveoloolithidae indet.	Clutch(es) (Location 3-5)	Coarse	Sihwa	Hanyom, Sihwa Basin, South Korea	Kim et al. (2009)
Faveoloolithidae indet.	Clutch(es) (Location 3-6)	Coarse	Sihwa	Hanyom, Sihwa Basin, South Korea	Kim et al. (2009)

Ootaxon/taxon	Material	Lithology	Formation	Locality	References
Faveoloolithidae indet.	Clutch(es) (Locations 4-1 JH, 4-2, 4-2, 4-3, and 4-4)	Coarse	Sihwa	Hanyom, Sihwa Basin, South Korea	Kim et al. (2009)
Faveoloolithidae indet.	Clutch(es) (Location 4-5)	Coarse	Sihwa	Hanyom, Sihwa Basin, South Korea	Kim et al. (2009)
Faveoloolithidae indet.	Clutch(es) (Location 5-2)	Coarse	Sihwa	Hanyom, Sihwa Basin, South Korea	Kim et al. (2009)
Faveoloolithidae indet.	Clutch(es) (Location 5-3)	Coarse	Sihwa	Hanyom, Sihwa Basin, South Korea	Kim et al. (2009)
Faveoloolithidae indet.	Clutch(es) (Location 5-4)	Coarse	Sihwa	Hanyom, Sihwa Basin, South Korea	Kim et al. (2009)
Faveoloolithidae indet.	Clutch(es) (Locations 6-1 H and 6-2)	Coarse	Sihwa	Hanyom, Sihwa Basin, South Korea	Kim et al. (2009)
Faveoloolithidae indet.	Clutch(es) (Locations 6-3 and 6-4)	Coarse	Sihwa	Hanyom, Sihwa Basin, South Korea	Kim et al. (2009)
Faveoloolithidae indet.	Clutch(es) (Location 6-5)	Coarse	Sihwa	Hanyom, Sihwa Basin, South Korea	Kim et al. (2009)
Faveoloolithidae indet.	Clutch(es)	Coarse	Sihwa	Hanyom, Sihwa Basin,	Kim et al. (2009)

Ootaxon/taxon	Material	Lithology	Formation	Locality	References
	(Location 6-6)			South Korea	
Faveoloolithidae indet.	Clutch(es) (Location 6-7)	Coarse	Sihwa	Hanyom, Sihwa Basin, South Korea	Kim et al. (2009)
Faveoloolithidae indet.	Clutch(es) (Location 6-8)	Coarse	Sihwa	Hanyom, Sihwa Basin, South Korea	Kim et al. (2009)
Faveoloolithidae indet.	Clutch(es) (Location 6-9)	Coarse	Sihwa	Hanyom, Sihwa Basin, South Korea	Kim et al. (2009)
Faveoloolithidae indet.	Clutch(es) (Location 9-1 D)	Coarse	Sihwa	Hanyom, Sihwa Basin, South Korea	Kim et al. (2009)
Faveoloolithidae indet.	Clutch(es) (Location 9-2)	Coarse	Sihwa	Hanyom, Sihwa Basin, South Korea	Kim et al. (2009)
Faveoloolithidae indet.	Clutch(es) (Location 9-3)	Coarse	Sihwa	Hanyom, Sihwa Basin, South Korea	Kim et al. (2009)
Faveoloolithidae indet.	Clutch(es) (Location 9-4)	Coarse	Sihwa	Hanyom, Sihwa Basin, South Korea	Kim et al. (2009)
Faveoloolithidae indet.	Clutch(es)	Coarse	Haman	Coast of Sinsudo Island, Samcheonpo, South Korea	Paik et al. (2012)
Faveoloolithidae indet.	Colonial nesting ground (one of 5 horisons)	Coarse	Los Llanos	Sanagasta, La Rioja, Argentina	Grellet-Tinner and Fiorelli (2010); Fiorelli et al. (2012)

Ootaxon/taxon	Material	Lithology	Formation	Locality	References
Faveoloolithidae indet.	Colonial nesting ground (one of 5 horisons)	Coarse*	Los Llanos	Sanagasta, La Rioja, Argentina	Grellet-Tinner and Fiorelli (2010); Fiorelli et al. (2012)
Faveoloolithidae indet.	Colonial nesting ground (one of 5 horisons)	Coarse*	Los Llanos	Sanagasta, La Rioja, Argentina	Grellet-Tinner and Fiorelli (2010); Fiorelli et al. (2012)
Faveoloolithidae indet.	Colonial nesting ground (one of 5 horisons)	Coarse*	Los Llanos	Sanagasta, La Rioja, Argentina	Grellet-Tinner and Fiorelli (2010); Fiorelli et al. (2012)
Faveoloolithidae indet.	Colonial nesting ground (one of 5 horisons)	Coarse*	Los Llanos	Sanagasta, La Rioja, Argentina	Grellet-Tinner and Fiorelli (2010); Fiorelli et al. (2012)
Faveoloolithidae indet.	Clutch(es) (Egg bed 3 of Salitral Ojo de Agua)	Coarse*	Allen	Arriagada I, Rio Negro, Argentina	Salgado et al. (2007)
Faveoloolithidae indet.	Clutch(es) (Egg bed 2 of Salitral de Santa Rosa-Salinas de Trapalco)	Coarse*	Allen	Santos I, Salitral de Santa Rosa-Salinas de Trapalco, Rio Negro, Argentina	Salgado et al. (2007)
Faveoloolithidae indet.	Clutch(es) (Egg bed 3 of Salitral de	Coarse*	Allen	Santos II and Berthe IV, Salitral de Santa Rosa-	Salgado et al. (2007)

Ootaxon/taxon	Material	Lithology	Formation	Locality	References
	Santa Rosa-Salinas de Trapalco)			Salinas de Trapalco, Rio Negro, Argentina	
Faveoloolithidae indet.	Clutch(es)	Coarse	NA	Algui Ulan Tsav, Gobi, Mongolia	Watabe et al. (2010)
Megaloolithidae					
Megaloolithus balasinorensis (/*baghensis*)	Clutch(es)	Coarse*	Lameta	Balasinor Quarry, Jetholi and Dhuvadiya, Kheda, Gujarat, India	Mohabey (1998)
M. dhoridungriensis	Clutch(es) with juvenile bones	Coarse*	Lameta	Dhoridungri, Gujarat, India	Mohabey (1998)
M. dhoridungriensis	Clutch(es) with hatchling bones	Coarse*	Lameta	Near Dhoridungri, Gujarat, India	Wilson et al. (2010)
M. khempurensis(/*walpurensis*?)	Clutch(es)	Coarse*	Lameta	Khempur and Werasa, Kheda, Gujarat, India	Mohabey (1998)
M. mammilare	Clutch(es) (one of multiple stratigraphic levels)	Fine	NA	Commune de Rousset, near Cairanne, Aix-en-Provence, France	Williams et al. (1984)
M. mammilare	Clutch(es) (one of multiple	Fine	NA	Commune de Chateauneuf Le Rouge,	Williams et al. (1984)

Ootaxon/taxon	Material	Lithology	Formation	Locality	References
	stratigraphic levels)			Aix-en-Provence, France	
M. matleyi (/*jabalpurensis*)	Clutch(es) (Site B)	Coarse*	Lameta	Pavna, Chandrapur, Maharashtra, India	Mohabey (1996; 1998)
M. matleyi (/*jabalpurensis*)	Nesting ground (Site A)	Coarse*	Lameta	Pavna, Chandrapur, Maharashtra, India	Mohabey (1996)
M. megadermus	Clutch(es)	Coarse*	Lameta	Dholidhanti and Paori, Dohad, Panchmahals, India	Mohabey (1998)
M. microtuberculata	Clutch(es)	Fine	NA	La Cairanne, Aix-en-Provence, France	Garcia and Vianey-Liaud (2001); Vianey-Liaud et al. (2003)
M. patagonicus (Titanosauridae)	Colonial nesting ground (Egg bed 3) with embryonic bones	Fine*	Anacleto	Auca Mahuevo, Neuquen, Patagonia, Argentina	Chiappe et al. (1998; 2001); Grellet-Tinner et al. (2004); Jackson et al. (2013)
M. patagonicus (Titanosauridae)	Colonial nesting ground (Egg bed 4)	Fine*	Anacleto	Auca Mahuevo, Neuquen, Patagonia, Argentina	Chiappe et al. (2001; 2004)
M. patagonicus (Titanosauridae)	Colonial nesting ground (Egg bed	Fine	Anacleto	Auca Mahuevo, Neuquen, Patagonia,	Chiappe et al. (2001; 2004)

Ootaxon/taxon	Material	Lithology	Formation	Locality	References
	2)			Argentina	
M. patagonicus (Titanosauridae)	Colonial nesting ground (Egg bed 1)	Fine	Anacleto	Auca Mahuevo, Neuquen, Patagonia, Argentina	Chiappe et al. (2001; 2004)
M. patagonicus (Titanosauridae)	Clutch(es)	Fine	Rio Colorado	Gran Neuquen neighbourhood, Neuquen, Patagonia, Argentina	Calvo et al. (1997)
M. phensaniensis (/*mohabeyi*)	Clutch(es)	Coarse*	Lameta	Phensani, Balasinor, Sonipur and Waniawao, Kheda, Gujarat, India	Mohabey (1998)
M. rahioliensis (/*cylindricus*)	Clutch(es)	Coarse*	Lameta	Rahioli, Kheda, Gujarat, India	Mohabey (1998)
M. siruguei	Clutch(es) (Egg horizon 1)	Fine*	Tremp	Coll de Nargo, Lleida, Catalonia, Spain	Jackson (2007); Vila et al. (2010a)
M. siruguei	Clutch(es) (Egg horizon 3)	Fine*	Tremp	Coll de Nargo, Lleida, Catalonia, Spain	Jackson (2007); Vila et al. (2010a)
M. siruguei	Clutch(es) (Egg horizon 5)	Fine*	Tremp	Coll de Nargo, Lleida, Catalonia, Spain	Jackson (2007); Vila et al. (2010a)
M. siruguei	Clutch(es) (Egg horizon 6)	Coarse*	Tremp	Coll de Nargo, Lleida, Catalonia, Spain	Jackson (2007); Vila et al. (2010a)

Ootaxon/taxon	Material	Lithology	Formation	Locality	References
M. siruguei	Clutch(es) (Egg horizon 7)	Coarse*	Tremp	Coll de Nargo, Lleida, Catalonia, Spain	Jackson (2007); Vila et al. (2010a)
M. siruguei	Clutch(es) (Egg horizon 8)	Coarse*	Tremp	Coll de Nargo, Lleida, Catalonia, Spain	Jackson (2007); Vila et al. (2010a)
M. siruguei	Clutch(es)	Fine*	Tremp	Font del Bullidor, Vallcebre Syncline, Spain	Vila et al. (2010b)
M. siruguei	Clutch(es)	Fine	NA	Commune de Fox-Amphoux, near Metisson, Aix-en-Provence, France	Williams et al. (1984)
M. siruguei	Clutch(es) (one of multiple stratigraphic levels)	Fine*	Tremp	Coll de Nargo, Lleida, Catalonia, Spain	Sellés et al. (2013)
M. siruguei	Clutch(es) (one of multiple stratigraphic levels)	Fine*	Tremp	Coll de Nargo, Lleida, Catalonia, Spain	Sellés et al. (2013)
M. siruguei	Clutch(es) (one of multiple	Fine*	Tremp	Coll de Nargo, Lleida, Catalonia, Spain	Sellés et al. (2013)

Ootaxon/taxon	Material	Lithology	Formation	Locality	References
	stratigraphic levels)				
M. siruguei	Clutch(es) (one of multiple stratigraphic levels)	Fine*	Tremp	Coll de Nargo, Lleida, Catalonia, Spain	Sellés et al. (2013)
M. siruguei	Clutch(es) (one of multiple stratigraphic levels)	Fine*	Tremp	Coll de Nargo, Lleida, Catalonia, Spain	Sellés et al. (2013)
M. siruguei	Clutch(es) (one of multiple stratigraphic levels)	Fine*	Tremp	Coll de Nargo, Lleida, Catalonia, Spain	Sellés et al. (2013)
M. siruguei	Clutch(es) (one of multiple stratigraphic levels)	Coarse	Tremp	Coll de Nargo, Lleida, Catalonia, Spain	Sellés et al. (2013)
M. siruguei	Clutch(es) (one of multiple stratigraphic	Fine*	Tremp	Coll de Nargo, Lleida, Catalonia, Spain	Sellés et al. (2013)

Ootaxon/taxon	Material	Lithology	Formation	Locality	References
	levels)				
M. siruguei	Clutch(es) (one of multiple stratigraphic levels)	Fine*	Tremp	Coll de Nargo, Lleida, Catalonia, Spain	Sellés et al. (2013)
M. siruguei	Clutch(es) (one of multiple stratigraphic levels)	Fine*	Tremp	Coll de Nargo, Lleida, Catalonia, Spain	Sellés et al. (2013)
M. siruguei	Clutch(es) (one of multiple stratigraphic levels)	Fine*	Tremp	Coll de Nargo, Lleida, Catalonia, Spain	Sellés et al. (2013)
M. siruguei	Clutch(es) (one of multiple stratigraphic levels)	Coarse	Tremp	Coll de Nargo, Lleida, Catalonia, Spain	Sellés et al. (2013)
M. siruguei	Clutch(es)	Fine	Tremp	Biscarri, Isona, Lleida, Spain	Lopez-Martinez et al. (2000)
M. siruguei	Clutch(es) (C1 nesting horizon)	Fine*	Densus-Ciula	Tustea, Hateg Basin, Romania	Grigorescu et al. (1994, 2010); Grigorescu

Ootaxon/taxon	Material	Lithology	Formation	Locality	References
					(2016); Botfalvai et al. (2017)
M. siruguei	Clutch(es) (C2 nesting horizon)	Fine*	Densus-Ciula	Tustea, Hateg Basin, Romania	Grigorescu et al. (1994, 2010); Grigorescu (2016); Botfalvai et al. (2017)
M. cf. *siruguei*	Clutch(es) (Horizon E)	Fine*	Sanpetru	Totesti-baraj, Hateg Basin, Romania	Codrea et al. (2002); Grigorescu et al. (1994; 2010); Grellet-Tinner et al. (2012)
M. cf. *siruguei*	Clutch(es) (Horizon A)	Fine*	Sanpetru	Totesti-baraj, Hateg Basin, Romania	Codrea et al. (2002); Grigorescu et al. (1994; 2010); Grellet-Tinner et al. (2012)
M. cf. *siruguei*	Clutch(es) (Horizon K)	Fine*	Sanpetru	Totesti-baraj, Hateg Basin, Romania	Codrea et al. (2002); Grigorescu et al. (1994; 2010); Grellet-Tinner et al. (2012)
M. cf. *siruguei*	Clutch(es) (Horizon B)	Fine*	Sanpetru	Totesti-baraj, Hateg Basin, Romania	Codrea et al. (2002); Grigorescu et al. (1994; 2010); Grellet-Tinner et

Ootaxon/taxon	Material	Lithology	Formation	Locality	References
					al. (2012)
M. cf. *siruguei*	Clutch(es) (Horizons G and H)	Fine*	Sanpetru	Totesti-baraj, Hateg Basin, Romania	Codrea et al. (2002); Grigorescu et al. (1994; 2010); Grellet-Tinner et al. (2012)
M. cf. *siruguei*	Clutch(es) (Find 8)	Fine*	Sanpetru	Nalat-Vad, Hateg Basin, Romania	Smith et al. (2002); Grigorescu et al. (1994; 2010)
M. cf. *siruguei*	Clutch(es) (Find 9)	Fine*	Sanpetru	Nalat-Vad, Hateg Basin, Romania	Smith et al. (2002); Grigorescu et al. (1994; 2010)
M. cf. *siruguei*	Clutch(es) (Find 2)	Fine*	Sanpetru	Nalat-Vad, Hateg Basin, Romania	Smith et al. (2002); Grigorescu et al. (1994; 2010)
M. cf. *siruguei*	Clutch(es) (Find 6)	Fine	Sanpetru	Nalat-Vad, Hateg Basin, Romania	Smith et al. (2002); Grigorescu et al. (1994; 2010)
M. cf. *siruguei*	Clutch(es) (Find 7)	Coarse*	Sanpetru	Nalat-Vad, Hateg Basin, Romania	Smith et al. (2002); Grigorescu et al. (1994; 2010)
M. sp.	Clutch(es)	Coarse*	Lameta	Salbardi, District	Srivastava and Mankar

Ootaxon/taxon	Material	Lithology	Formation	Locality	References
				Amravati, Maharashtra and Betul, India	(2015)
M. sp.	Colonial nesting ground	Coarse	Aren Sandstone	Barranc de la Costa Gran, Basturs, Tremp syncline, southern Pyrenees, Spain	Sanz et al. (1995); Sander et al. (1998); Diaz-Molina et al. (2007)
M. sp.	Clutch(es) (Horizon 1)	Fine	Tremp	Coll de Nargo, Lleida, Catalonia, Spain	Sander et al. (1998); Peitz (2001)
M. sp.	Clutch(es) (Horizon 2)	Fine	Tremp	Coll de Nargo, Lleida, Catalonia, Spain	Sander et al. (1998); Peitz (2001)
M. sp.	Clutch(es) (Horizon 3)	Fine	Tremp	Coll de Nargo, Lleida, Catalonia, Spain	Sander et al. (1998); Peitz (2001)
M. sp.	Clutch(es) (Horizon 4)	Fine	Tremp	Coll de Nargo, Lleida, Catalonia, Spain	Sander et al. (1998); Peitz (2001)
M. sp.	Clutch(es) (Horizon 5)	Fine	Tremp	Coll de Nargo, Lleida, Catalonia, Spain	Sander et al. (1998); Peitz (2001)
M. sp.	Clutch(es) (Horizon 6)	Fine	Tremp	Coll de Nargo, Lleida, Catalonia, Spain	Sander et al. (1998); Peitz (2001)
M. sp.	Clutch(es) (Horizon 7)	Fine	Tremp	Coll de Nargo, Lleida, Catalonia, Spain	Sander et al. (1998); Peitz (2001)
M. sp.	Clutch(es)	Fine*	Densus-Ciula	Tustea, Hateg Basin,	Venczel et al. (2015)

Ootaxon/taxon	Material	Lithology	Formation	Locality	References
				Romania	
M. sp. (Type 2A: probably *M. patagonicus*)	Clutch(es) (Egg level 3 of Salitral de Santa Rosa-Salinas de Trapalco)	Coarse*	Allen	Berthe IV, Salitral de Santa Rosa-Salinas de Trapalco, Rio Negro, Argentina	Salgado et al. (2007; 2009)
Megaloolithidae indet.	Clutch(es)	Coarse*	Allen	Bajo de Arriagada, Patagonia, Rio Negro, Argentina	Genise and Sarzetti (2011)
Megaloolithidae indet. (Type 2B)	Clutch(es) (Egg level 2 of Salitral Ojo de Agua)	Coarse	Allen	Arrigada III, Salitral Ojo de Agua, Rio Negro, Argentina	Salgado et al. (2007)
Megaloolithidae indet.	Clutch(es)	Coarse	Allen	Arrigada III, Rio Negro, Argentina	Salgado et al. (2007)
Megaloolithidae indet.	Clutch(es)	Coarse	Los Llanos	Tama, Sierra de Los Llanos, La Rioja, Argentina	Hechenleitner et al. (2016); Basilici et al. (2017)
"*Hypselosaurus priscus*"	Colonial nesting ground	Fine	NA (Marines Rouges Inferieures)	Rennes-le-chateau, Aude, France	Cousin et al. (1989)
Montanoolithidae					

Ootaxon/taxon	Material	Lithology	Formation	Locality	References
Montanoolithus strongorum	Clutch(es)	Coarse*	Two Medicine	Blackfeet Reservation, Montana, USA	Zelenitsky and Therrien (2008b)
Ovaloolithidae					
Ovaloolithus sp.	Clutch(es)	Fine	Chichengshan	Ganjiechang, Tiantai, Zhejiang, China	Fang et al. (2000)
Ovaloolithidae indet.	Clutch(es)	Fine*	Hasandong	Sumunri, Geumseong-myeon, Hadong, South Gyeongsang, South Korea	Lee (2003); Yun and Yang (1997); Paik et al. (2012)
Preprismatoolithus					
Preprismatoolithus coloradensis (*Allosaurus*)	Clutch(es)	Fine	Morrison	Young Locality, Delta County, Colorado, USA	Hirsch (1994)
Preprismatoolithus (*Lourinhanosaurus antunesi*)	Clutch(es) with embryonic bones	Fine	Lourihna	Pai Mogo, Lourinha, Estramadur, Portugal	Mateus et al. (1997); Antunes et al. (1998)
Prismatoolithidae					
Prismatoolithus gebiensis	Clutch(es)	Fine	Gaogou	Xixia Basin, Henan, China	Wang and Zhou (1995)
P. gebiensis	Clutch(es)	Fine	Djadokhta	Bayan Mandahu, Urad Houqi, Inner Mongolia,	Zhao and Li (1993)

Ootaxon/taxon	Material	Lithology	Formation	Locality	References
				China	
P. heyuanensis	Clutch(es)	Coarse	Dongyuan	Fengguang Village of Yuannan Town, Yuancheng District, Heyuan, Guangdong, China	Lü et al. (2006); Tanaka et al. (2012)
P. jenseni	Clutch(es)	Coarse	North Horn	Sauropod Locality, Emery County, Utah, USA	Jensen (1966); Bray (1999)
P. levis (*Troodon formosus*)	Clutch(es)	Fine	Oldman	New Egg Site, Devil's Coulee, Alberta, Canada	Zelenitsky and Hills (1996); this study (TMP 1994.179.1)
P. levis (*Troodon formosus*)	Clutch(es)	Fine*	Oldman	North Baby Butte, Devil's Coulee, Alberta, Canada	This study (TMP 1996.86.1)
P. levis (*Troodon formosus*)	Clutch(es)	Fine*	Two Medicine	Egg Mountain, Willow Creek Anticline and the Red Rock Locality, Choteau, Teton County, Montana, USA	Varricchio et al. (1997; 1999; 2002)
P. levis (*Troodon*	Clutch(es)	Fine	Two Medicine	Unnamed site, Willow	Varricchio et al. (2002;

Ootaxon/taxon	Material	Lithology	Formation	Locality	References
formosus)				Creek Anticline and the Red Rock Locality, Choteau, Teton County, Montana; Glacier County, Montana, USA	2015)
P. tiantaiensis	Clutch(es)	Fine	Chichengshan	Tiantai Basin, Zhejiang, China	Qian et al. (2008)
Protoceratopsidovum fluxuosum	Clutch(es)	Coarse	Barun-Goyot	Khermin-Tsav, Gobi, Mongolia	Mikhailov (1994b)
P. minimum	Clutch(es)	Coarse	Djadokhta	Baga-Tariach, Gobi, Mongolia	Mikhailov (1994b)
P. sincerum	Clutch(es)	Coarse	Djadokhta	Bain-Dzak, Gobi, Mongolia	Mikhailov (1994b)
P. sincerum	Clutch(es)	Coarse	Djadokhta	Main Field, Bain-Dzak, Gobi, Mongolia	Sabath (1991)
P. sincerum	Clutch(es)	Coarse	Djadokhta	Dashzeveg Sayr, Bain-Dzak, Gobi, Mongolia	Sabath (1991)
P. sp.	Clutch(es)	Coarse*	Djadokhta	Zhinst Tolgoi, Udyn Sayr, Gobi, Mongolia	Suzuki and Watabe (2000a)
P. sp.	Clutch(es)	Coarse	Djadokhta	Bain-Dzak, Gobi, Mongolia	Suzuki and Watabe (2000b)

Ootaxon/taxon	Material	Lithology	Formation	Locality	References
P. sp.	Clutch(es)	Coarse	Djadokhta	Tugrikin Shire, Gobi, Mongolia	Watabe et al. (2010)
Sankofa pyrenaica	Clutch(es)	Coarse	Aren	Serrat Pedrego, Montsec area, Lleida, Catalonia, Spain	Lopez-Martinez and Vicens (2012)
Prismatoolithus sp.	Clutch(es)	Fine*	Oldman	Little Diablo's Hill and Saddle, Devil's Coulee, Alberta, Canada	This study (TMP 2008.75.51)
P. levis (*Troodon formosus*)	Clutch(es) with embryonic bones	Fine*	Two Medicine	Egg Island, Choteau, Teton County, Montana, USA	Varricchio et al. (2002)
Spheroolithidae					
Paraspheroolithus sanwangbacunensis	Clutch(es)	Coarse	Dongyuan	Heyuan Basin, Guangdong, China	Fang et al. (2005)
Spheroolithus spheroides	Clutch(es)	Fine	Wangshi Group	Near Chinkongkou and Tsotan, Laiyang, Shangdong, China	Chow (1951; 1954)
S. sp.	Clutch(es) (one from at least two stratigraphic levels)	Fine*	Seonso	Bosung County, Chullanam-do, South Korea	Huh and Zelenitsky (2002); Kim et al. (2009); Paik et al. (2004)

Ootaxon/taxon	Material	Lithology	Formation	Locality	References
S. sp.	Clutch(es) (one from at least two stratigraphic levels)	Fine*	Seonso	Bosung County, Chullanam-do, South Korea	Huh and Zelenitsky (2002); Kim et al. (2009); Paik et al. (2004)
Spheroolithidae indet.	Clutch(es) (Site 2-lower horizon)	Fine	Goseong	Goseong, South Korea	Paik et al. (2012)
Spheroolithidae indet.	Clutch(es) (Site 3)	Fine*	Goseong	Goseong, South Korea	Paik et al. (2012)
Spheroolithidae indet.	Clutch(es) (Site 4)	Fine	Goseong	Goseong, South Korea	Paik et al. (2012)
Spheroolithidae indet.	Clutch(es)	Coarse	Barungoyot	Shiluut Ula, Gobi, Mongolia	Watabe et al. (2010)
Spheroolithidae indet.	Clutch(es)	Coarse*	Djadokhta	Dzamin Khond, Gobi, Mongolia	Watabe et al. (2010)
Maiasaura peeblesorum	Colonial nesting ground	Fine*	Two Medicine	Choteau, Teton County, Montana, USA	Horner and Makela (1979); Horner (1982)
Hypacrosaurus stebingeri	Clutch(es)	Fine*	Two Medicine	Blacktail Creek, Glacier County, Montana, USA	Horner and Currie (1994)
H. stebingeri	Clutch(es) (one of multiple stratigraphic levels)	Fine*	Oldman	Diablo's Hill, Devil's Coulee, Alberta, Canada	Horner and Currie (1994); this study (TMP 1989.79.53; TMP1988.79.36)
H. stebingeri	Clutch(es) (one of	Fine*	Oldman	Diablo's Hill, Devil's	This study (TMP

Ootaxon/taxon	Material	Lithology	Formation	Locality	References
	multiple stratigraphic levels)			Coulee, Alberta, Canada	1997.63.1)
Youngoolithidae					
Youngoolithus xiaguanensis	Clutch(es)	Fine	NA	Xianguan Basin, Neixiang County, Henan, China	Zhao (1979); Zhang (2010)
Oofamily incertae sedis					
Continuoolithus canadensis	Clutch(es)	Fine*	Two Medicine	Flaming Cliff locality, Willow Creek Anticline and the Red Rock Locality, Choteau, Teton County, Montana, USA	Jackson et al. (2015)
Massospondylus carinatus	Clutch(es) (one of four horisons)	Fine*	Upper Elliot	Golden Gate Highlands National Park, South Africa	Kitching (1979); Zelenitsky and Modesto (2002); Reisz et al. (2012)
M. carinatus	Clutch(es) (one of four horisons)	Fine*	Upper Elliot	Golden Gate Highlands National Park, South Africa	Kitching (1979); Zelenitsky and Modesto (2002); Reisz et al. (2012)

Ootaxon/taxon	Material	Lithology	Formation	Locality	References
M. carinatus	Clutch(es) (one of four horisons)	Fine*	Upper Elliot	Golden Gate Highlands National Park, South Africa	Kitching (1979); Zelenitsky and Modesto (2002); Reisz et al. (2012)
M. carinatus	Clutch(es) (one of four horisons)	Fine*	Upper Elliot	Golden Gate Highlands National Park, South Africa	Kitching (1979); Zelenitsky and Modesto (2002); Reisz et al. (2012)
Multicanaliculate eggs	Clutch(es)	Coarse*	Mercedes	Soriano, Uruguay	Faccio (1994)
Protoceratops andrewsi eggs	Clutch(es)	Fine	Djadokhta	Ukhaa Tolgod, Mongolia	Erickson et al. (2017)
Indeterminate dinosaur eggs	Clutch(es)	Coarse	Jindong	Goseong, South Korea	Lee (2003)

Supplementary Table S4. Lithology of dinosaur eggs and eggshells that are unlikely in-situ (assumedly ex-situ).

Note that * indicates that additional lithologic data were retrieved from the collection database at the Royal Tyrrell Museum of Palaeontology, Drumheller, Canada. Abbreviation: NA, not available.

Ootaxon/taxon	Material	Lithology	Formation	Locality	References
Arriagadoolithidae					
Arriagadoolithus patagoniensis (*Bonapartenykus ultimus*?)	Isolated egg(s) with *Bonapartenykus* bones	Coarse	Allen	Salitral Ojo de Agua, North Patagonia, Argentina	Agnolin et al. (2012)
A. patagoniensis	Eggshell(s) (Egg level 2 of Salitral Ojo de Agua)	Coarse	Allen	Arrigada III, Salitral Ojo de Agua, Rio Negro, Argentina	Salgado et al. (2007)
Triprismatoolithus sp.	Eggshell(s)	Coarse	Milk River	Verdigris Coulee, Alberta, Canada	Zelenitsky et al. (2017a)
Cairanoolithidae					
Cairanoolithus dughii	Eggshell(s)	Fine	Red Marls of Maurine Formation	Les Boudous and Gourg de l'Encantado, Upper Aude Valley, France	Fondevilla et al. (2016)
C. cf. *roussetensis*	Isolated egg(s) (one of multiple stratigraphic levels)	Fine	Tremp	Coll de Nargo, Lleida, Catalonia, Spain	Sellés et al. (2013)

Ootaxon/taxon	Material	Lithology	Formation	Locality	References
C. cf. *roussetensis*	Isolated egg(s) (one of multiple stratigraphic levels)	Fine	Tremp	Coll de Nargo, Lleida, Catalonia, Spain	Sellés et al. (2013)
Dendroolithidae (/Phaceloolithidae)					
Dendroolithus dendriticus	Eggshell(s)	Fine	Zhaoying	Xinping-Chimei Basin, Xixia, Henan, China	Fang et al. (2007)
Phaceloolithidae indet.	Isolated egg(s)	Coarse	Lourinha	Porto das Barcas, near Lourinha, Portugal	Ribeiro et al. (2014)
Dendroolithidae indet.	Eggshell(s)	Coarse	Barungoyot/ Nemegt	Khermeen Tsav, Gobi, Mongolia	Watabe et al. (2010)
Dendroolithidae indet.	Eggshell(s)	Coarse	?Nemegt	Yagaan Khovil, Gobi, Mongolia	Watabe et al. (2010)
Elongatoolithidae					
Elongatoolithus andrewsi	Eggshell(s) (one of multiple stratigraphic levels)	Fine	Zhutian	Nanxiong Basin, Guangdong, China	Fang et al. (2009)
E. andrewsi	Eggshell(s) (one of multiple stratigraphic levels)	Fine	Zhutian	Nanxiong Basin, Guangdong, China	Fang et al. (2009)

Ootaxon/taxon	Material	Lithology	Formation	Locality	References
E. andrewsi	Eggshell(s) (one of multiple stratigraphic levels)	Fine	Zhutian	Nanxiong Basin, Guangdong, China	Fang et al. (2009)
E. sp.	Eggshell(s)	Fine	Fenshuiao	Western Dongting Basin, Hunan, China	Zeng and Zhang (1979)
E. sp.	Eggshell(s)	Coarse	?Djadokhta	Toosgot area, Abdrant Nuru, Gobi, Mongolia	Watabe (2004)
E. sp.	Eggshell(s)	Fine	'Lower Formation' of Sasayama Group	Kamitaki, Tamba, Hyogo, Japan	Tanaka et al. (2016)
E. sp.	Eggshell(s)	Coarse	Nemegt	Khaichin Ula, Gobi, Mongolia	Suzuki and Watabe (2000b)
Macroelongatoolithus sp.	Eggshell fragments	Coarse	Wayan	Robinson Bonebed (IMNH2251), Bonneville County, Idaho, USA	Krumenacker et al. (2016)
Macroelongatoolithus sp.	Eggshell fragments	Fine	Wayan	Brockman Creek (IMNH2427), Bonneville County, Idaho, USA	Krumenacker et al. (2016)

Ootaxon/taxon	Material	Lithology	Formation	Locality	References
Macroelongatoolithus sp.	Eggshell fragments	Fine	Wayan	Jackknife Creek (IMNH2428), Bonneville County, Idaho, USA	Krumenacker et al. (2016)
Macroolithus yaotunensis	Isolated egg(s)	Fine	Pingling	Datang, Nanxiong Basin, Guangdong, China	Zhao et al. (2002)
Megafusoolithus qiaoxiaensis	Isolated egg(s)	Fine	Chichengshan	Shuangtang, Tiantai Basin, Zhejiang, China	Wang et al. (2010)
Paraelongatoolithus reticulatus	Isolated egg(s)	Fine	Chichengshan	Chengguan, Tiantai Basin, Zhejiang, China	Wang et al. (2010)
Trachoolithus faticanus	Eggshell(s)	Fine	Dushi Ula (Doshuul)	Buylyasutuin-Khuduk, Ubur-Khangay, Mongolia	Kurzanov and Mikhailov (1989); Mikhailov (1994b)
Elongatoolithidae indet.	Eggshell(s)	Fine	Quipa	Luanchuan, Tantou Basin, Henan, China	Xu (2007); Tanaka et al. (2011); Jiang et al. (2011)
Elongatoolithidae indet.	Isolated egg(s)	Fine	Dadaepo	Busan, South Korea	Paik et al. (2012)
Elongatoolithidae indet.	Eggshell(s)	Fine	Blesa	La Cantalera, Teruel,	Canudo et al. (2010)

Ootaxon/taxon	Material	Lithology	Formation	Locality	References
				Spain	
Elongatoolithidae indet.	Eggshell(s)	Coarse	Djadokhta	Dzamin Khond, Gobi, Mongolia	Suzuki and Watabe (2000b)
Elongatoolithidae indet.	Eggshell(s)	Coarse	Djadokhta	Bayn Dzak, Gobi, Mongolia	Suzuki and Watabe (2000b)
Elongatoolithidae indet.	Eggshell(s)	Coarse	Djadokhta	Mongot, Gobi, Mongolia	Suzuki and Watabe (2000b)
Elongatoolithidae indet.	Isolated egg(s)	Coarse	Nemegt	Bugin Tsav, Gobi, Mongolia	Watabe et al. (2010)
Elongatoolithidae indet.	Isolated egg(s) (Red Beds)	Coarse	Barungoyot/ Nemegt	Khermeen Tsav, Gobi, Mongolia	Watabe et al. (2010)
Elongatoolithidae indet.	Eggshell(s) (White Beds)	Coarse	Barungoyot/ Nemegt	Khermeen Tsav, Gobi, Mongolia	Watabe et al. (2010)
Elongatoolithidae indet.	Eggshell(s)	Coarse	Nemegt	Khermeen Tsav-II, Gobi, Mongolia	Watabe et al. (2010)
Elongatoolithidae indet.	Eggshell(s)	Fine	Nemegt	Shar Tsav, Gobi, Mongolia	Watabe et al. (2010)
Elongatoolithidae indet.	Eggshell(s)	Coarse	Djadokhta	Tugrikin Shire, Gobi, Mongolia	Watabe et al. (2010)
Elongatoolithidae indet.	Eggshell(s)	Coarse	Djadokhta	Dzamin Khond, Gobi, Mongolia	Watabe et al. (2010)

Ootaxon/taxon	Material	Lithology	Formation	Locality	References
Elongatoolithidae indet.	Eggshell(s)	Coarse	Djadokhta	Bortolgoi, Gobi, Mongolia	Watabe et al. (2010)
Elongatoolithidae indet.	Eggshell(s)	Coarse	?Nemegt	Yagaan Khovil, Gobi, Mongolia	Watabe et al. (2010)
Faveoloolithidae					
Faveoloolithus sp.	Isolated egg(s)	Fine	Seonso	Bosung County, Chullanam-do, South Korea	Huh and Zelenitsky (2002); Kim et al. (2009); Paik et al. (2004)
F. sp.	Isolated egg(s)	Fine	Seonso	Bosung County, Chullanam-do, South Korea	Huh and Zelenitsky (2002); Kim et al. (2009); Paik et al. (2004)
Parafaveoloolithus microporus	Isolated egg(s)	Fine	Laijia	Fangshan, Tiantai, Zhejiang, China	Zhang (2010)
Sphaerovum erbei	Eggshell(s)	Coarse	Asencio	Colonia, 12a. Police Section, stream Miguelete Tala, Uruguay	Mones (1980)
Faveoloolithidae indet.	Isolated egg(s) (Location 1-3)	Fine	Sihwa	Hanyom, Sihwa Basin, South Korea	Kim et al. (2009)
Faveoloolithidae indet.	Isolated egg(s) (Location 2 HH)	Fine	Sihwa	Hanyom, Sihwa Basin, South Korea	Kim et al. (2009)

Ootaxon/taxon	Material	Lithology	Formation	Locality	References
Faveoloolithidae indet.	Isolated egg(s) (Location 5-1 SH)	Fine	Sihwa	Hanyom, Sihwa Basin, South Korea	Kim et al. (2009)
Faveoloolithidae indet.	Isolated egg(s) (Location 6-2)	Coarse	Sihwa	Hanyom, Sihwa Basin, South Korea	Kim et al. (2009)
Faveoloolithidae indet.	Isolated egg(s) (Location 6-10)	Fine	Sihwa	Hanyom, Sihwa Basin, South Korea	Kim et al. (2009)
Faveoloolithidae indet.	Eggshell(s) (Egg level 2 of Salitral Ojo de Agua)	Coarse	Allen	Arriagada II, Salitral Ojo de Agua, Rio Negro, Argentina	Salgado et al. (2007)
Faveoloolithidae indet.	Eggshell(s) (Egg level 5 of Salitral Ojo de Agua)	Coarse	Allen	Cerro de Guerra, Salitral Ojo de Agua, Rio Negro, Argentina	Salgado et al. (2007)
Faveoloolithidae indet.	Eggshell(s) (Egg level 1 of Salitral de Santa Rosa-Salinas de Trapalco)	Coarse	Allen	Berthe II, Salitral de Santa Rosa-Salinas de Trapalco, Rio Negro, Argentina	Salgado et al. (2007)
Faveoloolithidae indet.	Eggshell(s) (Egg level 2 of Salitral de Santa Rosa-Salinas de	Coarse	Allen	Santos IV, Cerro Bonaparte, Berthe I, and Berthe III, Salitral de Santa	Salgado et al. (2007)

Ootaxon/taxon	Material	Lithology	Formation	Locality	References
	Trapalco)			Rosa-Salinas de Trapalco, Rio Negro, Argentina	
Faveoloolithidae indet.	Eggshell(s) (Egg level 3 of Salitral de Santa Rosa-Salinas de Trapalco)	Coarse	Allen	Cerro Bonaparte, Salitral de Santa Rosa-Salinas de Trapalco, Rio Negro, Argentina	Salgado et al. (2007)
Faveoloolithidae indet.	Eggshell(s) (Egg level 4 of Salitral de Santa Rosa-Salinas de Trapalco)	Coarse	Allen	Berthe V, Berthe VI, and Cerro Tortugas, Salitral de Santa Rosa-Salinas de Trapalco, Rio Negro, Argentina	Salgado et al. (2007)
Faveoloolithidae indet.	Eggshell(s) (Egg level 5 of Salitral de Santa Rosa-Salinas de Trapalco)	Coarse	Allen	Garcia I, Garcia II, and Cerro Tortugas, Salitral de Santa Rosa-Salinas de Trapalco, Rio Negro, Argentina	Salgado et al. (2007)
Megaloolithidae					

Ootaxon/taxon	Material	Lithology	Formation	Locality	References
Megaloolithus aureliensis	Isolated egg(s)	Fine	Tremp	Coll de Nargo, Lleida, Catalonia, Spain	Sellés et al. (2013)
cf. *M. aureliensis*	Eggshell(s)	Coarse	Tremp	Fontllonga 6, Tremp Basin, Southern Pyrenees, Lleida, Spain	Vianey-Liaud and Lopez-Martinez (1997)
M. baghensis	Eggshell(s)	Fine	Lameta	Anjar, Gujarat, India	Khosla and Sahni (1995)
M. cf. *baghensis*	Isolated egg(s)	Fine	Tremp	Coll de Nargo, Lleida, Catalonia, Spain	Sellés et al. (2013)
M. magharebiensis	Eggshell(s)	Fine	Irbzer	Oukdiksou syncline, Morocco	Garcia et al. (2003)
M. mammilare	Eggshell(s)	Fine	Red Marls of Maurine Formation	Roque Fumade, Upper Aude Valley, France	Fondevilla et al. (2016)
Possibly *M. mammillare*	Isolated egg(s)	Coarse	Tremp	Bastus, Tremp Basin, Southern Pyrenees, Lleida, Spain	Vianey-Liaud and Lopez-Martinez (1997)
M. mammillare?	Isolated egg(s)	Coarse	Tremp	Abella, Tremp Basin, Southern Pyrenees, Lleida, Spain	Vianey-Liaud and Lopez-Martinez (1997)
M. matleyi (/*jabalpurensis*)	Eggshell(s)	Coarse	Lameta	Patbaba ridge,	Mohabey (1998)

Ootaxon/taxon	Material	Lithology	Formation	Locality	References
				Jabalpur, Madhya Pradesh	
M. cf. *patagonicus* (Titanosauridae)	Eggshell(s)	Fine	Tremp	Costa de la Coma, Figuerola d'Orcau, Isona I Conca Della, South-Central Pyrenees, Spain	Bravo and Gaete (2015)
M. petralta	Eggshell(s)	Coarse	Tremp	Fontllonga 6, Tremp Basin, Southern Pyrenees, Lleida, Spain	Vianey-Liaud and Lopez-Martinez (1997)
M. petralta	Eggshell(s)	Coarse	Aren	Moro, Tremp Basin, Southern Pyrenees, Lleida, Spain	Vianey-Liaud and Lopez-Martinez (1997)
M. pseudomamillare? (or *M. petralta*)	Isolated egg(s)	Coarse	Tremp	Suterranya, Tremp Basin, Southern Pyrenees, Lleida, Spain	Vianey-Liaud and Lopez-Martinez (1997)
M. siruguei	Eggshell(s)	Fine	Aix-en-Provence	Rousset-Routiers, Aix-en-Provence, France	Vianey-Liaud et al. (1994)

Ootaxon/taxon	Material	Lithology	Formation	Locality	References
M. siruguei	Eggshell(s)	Coarse	Aix-en-Provence	Rousset-Village, Aix-en-Provence, France	Vianey-Liaud et al. (1994)
M. siruguei	Eggshell(s)	Fine	Tremp	Biscarri, Tremp Basin, Southern Pyrenees, Lleida, Spain	Vianey-Liaud and Lopez-Martinez (1997)
M. siruguei	Eggshell(s) (one of multiple stratigraphic levels)	Fine	Tremp	Coll de Nargo, Lleida, Catalonia, Spain	Sellés et al. (2013)
M. siruguei	Eggshell(s) (one of multiple stratigraphic levels)	Coarse	Tremp	Coll de Nargo, Lleida, Catalonia, Spain	Sellés et al. (2013)
M. siruguei	Eggshell(s) (one of multiple stratigraphic levels)	Fine	Tremp	Coll de Nargo, Lleida, Catalonia, Spain	Sellés et al. (2013)
M. siruguei	Eggshell(s) (one of multiple stratigraphic levels)	Fine	Tremp	Coll de Nargo, Lleida, Catalonia, Spain	Sellés et al. (2013)
M. siruguei	Eggshell(s) (one of multiple stratigraphic levels)	Fine	Tremp	Coll de Nargo, Lleida, Catalonia, Spain	Sellés et al. (2013)

Ootaxon/taxon	Material	Lithology	Formation	Locality	References
M. siruguei	Eggshell(s) (one of multiple stratigraphic levels)	Fine	Tremp	Coll de Nargo, Lleida, Catalonia, Spain	Sellés et al. (2013)
M. siruguei	Eggshell(s) (one of multiple stratigraphic levels)	Fine	Tremp	Coll de Nargo, Lleida, Catalonia, Spain	Sellés et al. (2013)
M. siruguei	Eggshell(s) (one of multiple stratigraphic levels)	Coarse	Tremp	Coll de Nargo, Lleida, Catalonia, Spain	Sellés et al. (2013)
M. siruguei	Eggshell(s) (one of multiple stratigraphic levels)	Fine	Tremp	Coll de Nargo, Lleida, Catalonia, Spain	Sellés et al. (2013)
M. siruguei	Eggshell(s) (one of multiple stratigraphic levels)	Fine	Tremp	Coll de Nargo, Lleida, Catalonia, Spain	Sellés et al. (2013)
M. siruguei	Eggshell(s) (one of multiple stratigraphic levels)	Coarse	Tremp	Coll de Nargo, Lleida, Catalonia, Spain	Sellés et al. (2013)
M. siruguei	Eggshell(s) (one of multiple stratigraphic levels)	Fine	Tremp	Coll de Nargo, Lleida, Catalonia, Spain	Sellés et al. (2013)

Ootaxon/taxon	Material	Lithology	Formation	Locality	References
M. cf. *siruguei*	Eggshell(s) (one of at least two eggshell-bearing levels)	Fine	Densus-Ciula	Livezi, Hateg Basin, Romania	Grigorescu et al. (1994; 2010)
M. cf. *siruguei*	Eggshell(s) (one of at least two eggshell-bearing levels)	Fine	Densus-Ciula	Livezi, Hateg Basin, Romania	Grigorescu et al. (1994; 2010)
M. sp.	Eggshell(s)	Fine	NA	Cruzy (Quarantes basin, Herault) and Vitrolles-Couperigne (Aix basin, Bouches-du-Rhone), France	Garcia (2000)
M. sp.	Eggshell(s)	Coarse	Aren	Basturs, Catalunya, Spain	Peitz (2001)
M. sp.	Isolated egg(s)	Fine	Tremp	Gosol, Catalunya, Spain	Peitz (2001)
Pseudomegaloolithus atlasi	Eggshell(s)	Fine	Tendrara red beds	Douar Lgara, Tendrara High Plateaus, Morocco	Chassagne-Manoukian et al. (2013)
Megaloolithidae indet.	Isolated egg(s)	Fine	NA	Commune de	Williams et al. (1984)

Ootaxon/taxon	Material	Lithology	Formation	Locality	References
				Pourcieux, Aix-en-Provence, France	
Megaloolithidae indet.	Eggshell(s) (One of five horizons)	Coarse	Galula Formation of Red Sandstone Group	TZ-07, 20 km west of the town of Mbeya in the Mbeya District, southwestern Tanzania	Gottfried et al. (2004)
Megaloolithidae indet.	Eggshell(s) (One of five horizons)	Coarse	Galula Formation of Red Sandstone Group	TZ-07, 20 km west of the town of Mbeya in the Mbeya District, southwestern Tanzania	Gottfried et al. (2004)
Megaloolithidae indet.	Eggshell(s) (One of five horizons)	Coarse	Galula Formation of Red Sandstone Group	TZ-07, 20 km west of the town of Mbeya in the Mbeya District, southwestern Tanzania	Gottfried et al. (2004)
Megaloolithidae indet.	Eggshell(s) (One of five horizons)	Coarse	Galula Formation of Red Sandstone	TZ-07, 20 km west of the town of Mbeya in the Mbeya District, southwestern	Gottfried et al. (2004)

Ootaxon/taxon	Material	Lithology	Formation	Locality	References
			Group	Tanzania	
Megaloolithidae indet.	Eggshell(s) (One of five horizons)	Coarse	Galula Formation of Red Sandstone Group	TZ-07, 20 km west of the town of Mbeya in the Mbeya District, southwestern Tanzania	Gottfried et al. (2004)
Megaloolithidae indet.	Eggshell(s)	Coarse	Marilia	Peiropolis, Baruru Basin, Marilia County, San Paulo State, Brazil	Ribeiro (2002)
Megaloolithidae indet.	Eggshell(s)	Coarse	Marilia	Ponte Alta, Baruru Basin, Marilia County, San Paulo State, Brazil	Ribeiro (2002)
Megaloolithidae indet. (Type 2A)	Type 2A eggshell(s) (Egg level 4 of Salitral Ojo de Agua)	Coarse	Allen	Arrigada I, Salitral Ojo de Agua, Rio Negro, Argentina	Salgado et al. (2007)
Megaloolithidae indet. (Type 2A)	Type 2A eggshell(s) (Egg level 1 of Salitral de Santa	Coarse	Allen	Berthe II, Salitral de Santa Rosa-Salinas de Trapalco, Rio Negro,	Salgado et al. (2007)

Ootaxon/taxon	Material	Lithology	Formation	Locality	References
	Rosa-Salinas de Trapalco)			Argentina	
Megaloolithidae indet. (Type 2A)	Type 2A eggshell(s) (Egg level 2 of Salitral de Santa Rosa-Salinas de Trapalco)	Coarse	Allen	Cerro Bonaparte, Salitral de Santa Rosa-Salinas de Trapalco, Rio Negro, Argentina	Salgado et al. (2007)
Megaloolithidae indet. (Type 2A)	Type 2A isolated egg(s) (Egg level 4 of Salitral de Santa Rosa-Salinas de Trapalco)	Coarse	Allen	Berthe IV, Salitral de Santa Rosa-Salinas de Trapalco, Rio Negro, Argentina	Salgado et al. (2007)
Megaloolithidae indet. (Type 2A)	Type 2A eggshell(s) (Egg level 5 of Salitral de Santa Rosa-Salinas de Trapalco)	Coarse	Allen	Cerro Turtugas, Salitral de Santa Rosa-Salinas de Trapalco, Rio Negro, Argentina	Salgado et al. (2007)
Megaloolithidae indet. (Type 2B)	Type 2B eggshell(s) (Egg level 2 of Salitral de Santa Rosa-Salinas de	Coarse	Allen	Berthe III, Salitral de Santa Rosa-Salinas de Trapalco, Rio Negro, Argentina	Salgado et al. (2007)

Ootaxon/taxon	Material	Lithology	Formation	Locality	References
	Trapalco)				
Megaloolithidae indet. (Type 2B)	Type 2B eggshell(s) (Egg level 3 of Salitral de Santa Rosa-Salinas de Trapalco)	Coarse	Allen	Mansilla I and II, Salitral de Santa Rosa-Salinas de Trapalco, Rio Negro, Argentina	Salgado et al. (2007)
Megaloolithidae indet. (Type 2B)	Type 2B eggshell(s) (Egg level 4 of Salitral de Santa Rosa-Salinas de Trapalco)	Coarse	Allen	Berthe V and VI, Salitral de Santa Rosa-Salinas de Trapalco, Rio Negro, Argentina	Salgado et al. (2007)
Megaloolithidae indet. (Type 2B)	Type 2B eggshell(s) (Egg level 5 of Salitral de Santa Rosa-Salinas de Trapalco)	Coarse	Allen	Garcia I, Salitral de Santa Rosa-Salinas de Trapalco, Rio Negro, Argentina	Salgado et al. (2007)
"*Hypselosaurus priscus*"	Eggshell(s)	Coarse	NA	Rennes-le-chateau, Aude, France	Cousin et al. (1989)
Montanoolithidae					
Montanoolithus labadousensis	Eggshell(s)	Fine	NA	Rennes-le-chateau, Aude, France	Beetschen et al. (1977); Vila et al. (2017)

Ootaxon/taxon	Material	Lithology	Formation	Locality	References
M. cf. *strongorum*	Eggshell(s)	Fine	Willow Creek	Todd Creek area, Alberta Canada	Zelenitsky et al. (2017b)
Ovaloolithidae					
Ovaloolithidae indet.	Eggshell(s)	Fine	NA	Tel Ulan Chaltsai (Mogoin Daatsyn Khuduk), Mongolia	Watabe et al. (2010)
Pinnatoolithae					
Lanceoloolithus xiapingensis	Eggshell(s) (one of multiple stratigraphic levels)	Coarse	Zhutian	Nanxiong Basin, Guangdong, China	Fang et al. (2009)
L. xiapingensis	Eggshell(s) (one of multiple stratigraphic levels)	Coarse	Zhutian	Nanxiong Basin, Guangdong, China	Fang et al. (2009)
Polyclonoolithidae					
Polyclonoolithus yangjiagouensis	Isolated egg(s)	Fine	Hekou Group	Yangjiagou, Lanzhou-Minhe Basin, Gansu, China	Xie et al. (2016)
Preprismatoolithus					
Preprismatoolithus (*Lourinhanosaurus antunesi*)	Eggshell(s)	Fine	Lourinha	Casal da Rola, near Lourinha, Portugal	Ribeiro et al. (2014)
P. coloradensis	Isolated egg(s)	Fine	Morrison	Cleveland-Lloyd	Hirsch et al. (1989);

Ootaxon/taxon	Material	Lithology	Formation	Locality	References
(*Allosaurus*)				Dinosaur Quarry, Emery County, Utah, USA	Hirsch (1994)
P. coloradensis (*Allosaurus*)	Eggshell(s)	Fine	Morrison	Garden Park Area, near Canyon City, Colorado, USA	Hirsch (1994)
Prismatoolithidae					
Prismatoolithus carboti	Isolated egg(s)	Fine	Villeveyrac-Meze Basin	Grande Marquise, Herault, France	Garcia et al. (2000)
P. carboti	Eggshell(s)	Fine	Villeveyrac-Meze Basin	Neuve, Bouches-du-Rhone, France	Garcia et al. (2000)
P. cf. *levis*	Eggshell(s)	Fine	Willow Creek	Todd Creek area, Alberta Canada	Zelenitsky et al. (2017b)
aff. *P. matellensis*	Eggshell(s)	Coarse	Tremp	Fontllonga 6, Tremp Basin, Southern Pyrenees, Lleida, Spain	Vianey-Liaud and Lopez-Martinez (1997)
P. tenuis	Eggshell(s)	Coarse	Tremp	Fontllonga 6, Tremp Basin, Southern Pyrenees, Lleida, Spain	Vianey-Liaud and Lopez-Martinez (1997)

Ootaxon/taxon	Material	Lithology	Formation	Locality	References
Protoceratopsidovum fluxuosum	Eggshell(s)	Coarse	NA	Nemegt, Gobi, Mongolia	Sabath (1991)
P. minimum	Isolated egg(s)	Coarse	Nemegt	Altan Ula IV, Gobi, Mongolia	Sabath (1991)
P. sp.	Eggshell(s)	Coarse	Djadokhta	Bayn Dzak, Gobi, Mongolia	Suzuki and Watabe (2000b)
P. sp.	Eggshell(s)	Coarse	Djadokhta	Bayn Dzak, Gobi, Mongolia	Watabe et al. (2010)
Sankofa pyrenaica	Eggshell(s)	Coarse	Aren	Urbanizacion Montsec, Montsec area, Lleida, Catalonia, Spain	Lopez-Martinez and Vicens (2012)
Trigonoolithus amoae	Eggshell(s)	Fine	Belsa	La Cantalera site, Josa, Teruel, northern Spain	Canudo et al. (2010); Moreno-Azanza et al. (2014b)
Prismatoolithus sp.	Eggshell(s)	Coarse	Dinosaur Park	Bonebed BB098, Dinosaur Provincial Park, Alberta	Zelenitsky and Sloboda (2005)
P. sp.	Eggshell(s)	Fine	Blesa	La Cantalera, Teruel, Spain	Canudo et al. (2010)
P. sp.	Eggshell(s)	Coarse	Fruitland	NMMNH L-4010,	Tanaka et al. (2011)

Ootaxon/taxon	Material	Lithology	Formation	Locality	References
				Bisti/De-na-zin Wilderness area, San Juan Basin, New Mexico	
P. sp.	Eggshell(s)	Fine	'Lower Formation' of Sasayama Group	Kamitaki, Tamba, Hyogo, Japan	Tanaka et al. (2016)
P. sp.	Eggshell(s)	Fine	Willow Creek	Whiskey Gap area and Todd Creek area, Alberta Canada	Zelenitsky et al. (2017b)
P. sp.	Eggshell(s)	Coarse	Milk River	Verdigris Coulee, Alberta, Canada	Zelenitsky et al. (2017a)
cf. *Prismatoolithus*	Eggshell(s)	Fine	Late Cretaceous	Cruzy (Quarantes basin, Herault) and Vitrolles-Couperigne (Aix basin, Bouches-du-Rhone), France	Garcia (2000)
Prismatoolithus?	Eggshell(s)	Fine	Irbzer	Oukdiksou syncline, Morocco	Garcia et al. (2003)
Prismatoolithidae indet.	Eggshell(s)	Fine	'Lower	Kamitaki, Tamba,	Tanaka et al. (2016)

Ootaxon/taxon	Material	Lithology	Formation	Locality	References
			Formation' of Sasayama Group	Hyogo, Japan	
Similifaveoolithidae					
Similifaveoloolithus gongzhulingensis	Isolated egg(s)	Fine	Quantou	Liufangzi, Gongzhuling, Jilin, China	Wang et al. (2006; 2013)
Spheroolithidae					
Guegoolithus turolensis	Eggshell(s)	Fine	Blesa	Galve, Teruel, Spain	Moreno-Azanza et al. (2014a)
G. turolensis	Eggshell(s)	Coarse	Camarillas	Galve, Teruel, Spain	Moreno-Azanza et al. (2014a)
*Spheroolithus albertensis**	Eggshell(s)	Coarse	Dinosaur Park	Bonebed BB031, Dinosaur Provincial Park, Alberta	Zelenitsky and Sloboda (2005)
*S. albertensis**	Eggshell(s)	Fine	Dinosaur Park	Bonebed BB104, Dinosaur Provincial Park, Alberta	Zelenitsky and Sloboda (2005)
S. cf. *albertensis*	Eggshell(s)	Fine	Willow Creek	Whiskey Gap area and Todd Creek area, Alberta Canada	Zelenitsky et al. (2017b)

Ootaxon/taxon	Material	Lithology	Formation	Locality	References
S. cf. *choteauensis*	Eggshell(s)	Fine	Willow Creek	Junction of the Oldman River and Callum Creek, Whiskey Gap area, and Todd Creek area, Alberta Canada	Zelenitsky et al. (2017b)
S. cf. *choteauensis*	Eggshell(s)	Coarse	Milk River	Verdigris Coulee, Alberta, Canada	Zelenitsky et al. (2017a)
S. europaeus	Eggshell(s)	Coarse	Tremp	Pont d'Orrit, Tremp, Lleida, Catalonia, Spain	Sellés et al. (2014)
S. megadermus	Isolated egg(s)	Fine	Quantou	Changtu, Liaoning, China	Liu et al. (2013)
S. spheroides (including *S. chiangchiugtingensis*)	Isolated egg(s)	Fine	Quantou	Changtu, Liaoning, China	Liu et al. (2013)
S. sp.	Isolated egg(s)	Fine	Seonso	Bosung County, Chullanam-do, South Korea	Huh and Zelenitsky (2002); Kim et al. (2009); Paik et al. (2004)
S. sp.	Isolated egg(s)	Fine	Seonso	Bosung County, Chullanam-do, South Korea	Huh and Zelenitsky (2002); Kim et al. (2009)

Ootaxon/taxon	Material	Lithology	Formation	Locality	References
S. sp.	Eggshell(s)	Fine	'Lower Formation' of Sasayama Group	Kamitaki, Tamba, Hyogo, Japan	Tanaka et al. (2016)
S. sp.	Eggshell(s) with perinatal *Saurolophus angustirostris* bones	Coarse	Nemegt	Dragon's Tomb' dinosaur locality, Gobi, Mongolia	Dewaele et al. (2015)
Spheroolithus?	Eggshell(s) (one of multiple stratigraphic levels)	Fine	Lameta	Dongargaon, Pisdura, Polgaon and Tidkepar in Kholdoda and Chandrapur, Maharashtra, India	Mohabey (1996; 2001)
Spheroolithus?	Eggshell(s) (one of multiple stratigraphic levels)	Coarse	Lameta	Dongargaon, Pisdura, Polgaon and Tidkepar in Kholdoda and Chandrapur, Maharashtra, India	Mohabey (1996; 2001)
Spheroolithidae indet.	Eggshell(s)	Coarse	?Djadokhta	Toosgot area, Abdrant Nuru, Gobi, Mongolia	Watabe (2004)

Ootaxon/taxon	Material	Lithology	Formation	Locality	References
Spheroolithidae indet.	Eggshell(s) (Site 1: Lower Horizon)	Fine	Goseong	Goseong, South Korea	Paik et al. (2012)
Spheroolithidae indet.	Isolated egg(s) (Site 1: Upper Horizon)	Coarse	Goseong	Goseong, South Korea	Paik et al. (2012)
Spheroolithidae indet.	Isolated egg(s) (Site 2: Upper Horizon)	Fine	Goseong	Goseong, South Korea	Paik et al. (2012)
Spheroolithidae indet.	Eggshell(s)	Fine	Nemegt	Shar Tsav, Gobi, Mongolia	Watabe et al. (2010)
Spheroolithidae indet.	Eggshell(s)	Fine	Djadokhta	Bortolgoi, Gobi, Mongolia	Watabe et al. (2010)
Spheroolithidae indet.	Eggshell(s)	Coarse	?Nemegt	Yagaan Khovil, Gobi, Mongolia	Watabe et al. (2010)
cf. Spheroolithidae	Eggshell(s)	Fine	Blesa	La Cantalera, Teruel, Spain	Canudo et al. (2010)
Oofamily incertae sedis					
Ageroolithus fontllongensis	Eggshell(s)	Coarse	Tremp	Fontllonga 6, Tremp Basin, Southern Pyrenees, Lleida, Spain	Vianey-Liaud and Lopez-Martinez (1997)
A. fontllongensis	Eggshell(s)	Coarse	Aren	Moro, Tremp Basin, Southern Pyrenees,	Vianey-Liaud and Lopez-Martinez (1997)

Ootaxon/taxon	Material	Lithology	Formation	Locality	References
				Lleida, Spain	
cf. *Ageroolithus*	Eggshell(s)	Fine	NA	Vitrolles-Couperigne, France	Garcia (2000)
Continuoolithus canadensis	Eggshell(s)	Fine	Oldman	Little Diablo's Hill, Devil's Coulee, Alberta, Canada	Zelenitsky et al. (1996)
C. canadensis	Eggshell(s)	Fine	Oldman	North Baby Butte, Devil's Coulee, Alberta, Canada	Zelenitsky et al. (1996)
C. canadensis	Eggshell(s)	Fine	Oldman	Faye Walker's Coulee, Devil's Coulee, Alberta, Canada	Zelenitsky et al. (1996)
C. canadensis	Eggshell(s)	Fine	Oldman	Juvie Camp, Devil's Coulee, Alberta, Canada	Zelenitsky et al. (1996)
C. canadensis	Eggshell(s)	Fine	Oldman	Knight's Ranch, Alberta, Canada	Zelenitsky et al. (1996)
*C. canadensis**	Eggshell(s)	Coarse	Dinosaur Park	Bonebed BB031, Dinosaur Provincial Park, Alberta, Canada	Zelenitsky and Sloboda (2005)
*C. canadensis**	Eggshell(s)	Coarse	Dinosaur	Bonebed BB098,	Zelenitsky and Sloboda

Ootaxon/taxon	Material	Lithology	Formation	Locality	References
			Park	Dinosaur Provincial Park, Alberta, Canada	(2005)
*C. canadensis**	Eggshell(s)	Fine	Dinosaur Park	Bonebed BB104, Dinosaur Provincial Park, Alberta, Canada	Zelenitsky and Sloboda (2005)
C. canadensis	Eggshell(s)	Coarse	Fruitland	NMMNH L-4010, Bisti/De-na-zin Wilderness area, San Juan Basin, New Mexico, USA	Tanaka et al. (2011)
C. cf. *canadensis*	Eggshell(s)	Fine	Willow Creek	Whiskey Gap area, Alberta Canada	Zelenitsky et al. (2017b)
C. cf. *canadensis*	Eggshell(s)	Coarse	Milk River	Verdigris Coulee, Alberta, Canada	Zelenitsky et al. (2017a)
cf. *Continuoolithus*	Eggshell(s)	Fine	Aguja	"Purple Hill" microsite, Rattlesnake Mountain, Big Bend National Park, Texas, USA	Welsh and Sankey (2005)
Ornithoid basic type and ratite morphotype	Eggshell(s)	Fine	Morrison	Rio Puerco drainage, Arch Mesa, Sandoval	Bray and Lucas (1997)

Ootaxon/taxon	Material	Lithology	Formation	Locality	References
(*Continuoolithus*?)				County, New Mexico, USA	
Deinonychus antirrhopus	Isolated egg(s) with embryonic *Deinonychus antirrhopus* bones	Fine	Cloverly	'Cashen Pocket', Crow Indian reservation, Big Horn County, Montana, USA	Makovicky and Grellet-Tinner (2001); Grellet-Tinner and Makovicky (2006)
Dispersituberoolithus exilis	Eggshell(s)	Fine	Oldman	Little Diablo's Hill, Devil's Coulee, Alberta, Canada	Zelenitsky et al. (1996)
Probably *Lufengosaurus*	Eggshell(s) with embryonic possible *Lufengosaurus* bones	Fine	Lower Lufeng	Kunming, Yunnan, China	Reisz et al. (2013)
Multicanaliculate eggs	Eggshell(s)	Coarse	Mercedes	Soriano, Uruguay	Faccio (1994)
Multicanaliculate eggs	Eggshell(s)	Coarse	Mercedes	Algorta, Uruguay	Faccio (1994)
Nipponoolithus ramosus	Eggshell(s)	Fine	'Lower Formation' of Sasayama Group	Kamitaki, Tamba, Hyogo, Japan	Tanaka et al. (2016)
Porituberoolithus	Eggshell(s)	Fine	Oldman	Little Diablo's Hill,	Zelenitsky et al. (1996)

Ootaxon/taxon	Material	Lithology	Formation	Locality	References
warnerensis				Devil's Coulee, Alberta, Canada	
P. warnerensis	Eggshell(s)	Fine	Oldman	Faye Walker's Coulee, Devil's Coulee, Alberta, Canada	Zelenitsky et al. (1996)
P. warnerensis	Eggshell(s)	Fine	Oldman	Knight's Ranch, Alberta, Canada	Zelenitsky et al. (1996)
*P. warnerensis**	Eggshell(s)	Coarse	Dinosaur Park	Bonebed BB031, Dinosaur Provincial Park, Alberta, Canada	Zelenitsky and Sloboda (2005)
P. warnerensis	Eggshell(s)	Fine	Willow Creek	Whiskey Gap area and Todd Creek area, Alberta Canada	Zelenitsky et al. (2017b)
P. warnerensis	Eggshell(s)	Coarse	Fruitland	NMMNH L-4010, Bisti/De-na-zin Wilderness area, San Juan Basin, New Mexico, USA	Tanaka et al. (2011)
P. cf. *warnerensis*	Eggshell(s)	Coarse	Milk River	Verdigris Coulee, Alberta, Canada	Zelenitsky et al. (2017a)
cf. *Porituberoolithus*	Eggshell(s)	Fine	Aguja	"Purple Hill"	Welsh and Sankey

Ootaxon/taxon	Material	Lithology	Formation	Locality	References
				microsite, Rattlesnake Mountain, Big Bend National Park, Texas, USA	(2005)
Pseudogeckoolithus nodosus	Eggshell(s)	Coarse	Tremp	Fontllonga 6, Tremp Basin, Southern Pyrenees, Lleida, Spain	Vianey-Liaud and Lopez-Martinez (1997)
Pseudogeckoolithus?	Eggshell(s)	Fine	NA	Cruzy (Quarantes basin, Herault), France	Garcia (2000)
*Reticuloolithus hirschi**	Eggshell(s)	Fine	Dinosaur Park	Bonebed BB104, Dinosaur Provincial Park, Alberta, Canada	Zelenitsky and Sloboda (2005)
Tristraguloolithus cracioides	Eggshell(s)	Fine	Oldman	Knight's Ranch, Alberta, Canada	Zelenitsky et al. (1996)
T. cracioides	Eggshell(s)	Fine	Oldman	Little Diablo's Hill, Devil's Coulee, Alberta, Canada	Zelenitsky et al. (1996)
T. cracioides	Eggshell(s)	Fine	Oldman	Faye Walker's Coulee, Devil's Coulee,	Zelenitsky et al. (1996)

Ootaxon/taxon	Material	Lithology	Formation	Locality	References
				Alberta, Canada	
Indeterminate theropod eggshell	Eggshell(s)	Coarse	Fruitland	NMMNH L-4010, Bisti/De-na-zin Wilderness area, San Juan Basin, New Mexico, USA	Tanaka et al. (2011)
Dinosauroid spherulithic basic type	Eggshell(s)	Fine	Aguja	"Purple Hill" microsite, Rattlesnake Mountain, Big Bend National Park, Texas, USA	Welsh and Sankey (2005)
?Prismatoolithidae	Eggshell(s)	Coarse	Tremp	Fontllonga 6, Tremp Basin, Southern Pyrenees, Lleida, Spain	Vianey-Liaud and Lopez-Martinez (1997)
?Prismatoolithidae	Eggshell(s)	Fine	Morrison	Gallison Locality, Delta County, Colorado, USA	Hirsch (1994)
Dinosauroid prismatic basic type	Eggshell(s)	Fine	Aguja	"Purple Hill" microsite, Rattlesnake Mountain, Big Bend	Welsh and Sankey (2005)

Ootaxon/taxon	Material	Lithology	Formation	Locality	References
				National Park, Texas, USA	
Ratite morphotype	Eggshell(s)	Fine	Aguja	"Purple Hill" microsite, Rattlesnake Mountain, Big Bend National Park, Texas, USA	Welsh and Sankey (2005)
Prismatic morphotype	Eggshell(s)	Fine	NA	La Neuve and Grande Marquise, France	Garcia (2000)
Indet. type	Isolated egg(s)	Fine	Oxford Clay	Oxford Clay, Peterborough, England	Liston and Chapman (2014)
Indet. Type (thin ridged type)	Eggshell(s)	Fine	Cloverly	MOR CL-121, Bridger, Carbon County, Montana, USA	Maxwell and Horner (1994)
Indet. Type (thick ridged type)	Eggshell(s)	Fine	Cloverly	MOR CL-121, Bridger, Carbon County, Montana, USA	Maxwell and Horner (1994)
Indet. Type (nodes type)	Eggshell(s)	Fine	Cloverly	MOR CL-121,	Maxwell and Horner

Ootaxon/taxon	Material	Lithology	Formation	Locality	References
				Bridger, Carbon County, Montana, USA	(1994)
Indet. type	Eggshell(s)	Fine	Morrison	Callison Locality, Delta County, Colorado, USA	Hirsch (1994)

Supplementary Table S5. List of ootaxa and their possible parental taxa used in this study.

Oofamily	Parental taxa	Identification based on:	Reference
Dendroolithidae (also known as Phaceloolithidae)	*Torvosaurus* and possibly therizinosauroids	Embryos	Manning et al. (1997); Kundrat et al. (2008); Araujo et al. (2013); Ribeiro et al. (2014)
Elongatoolithidae	Oviraptorosauria	Embryos, a gravid adult, and adults atop clutches	Dong and Currie (1996); Clark et al., (1999); Norell et al. (2001); Sato et al. (2005); Cheng et al. (2008); Weishampel et al. (2008); Fanti et al. (2012); Wang et al. (2016)
Faveoloolithidae	?Sauropodomorpha	Eggshell morphology	Grellet-Tinner and Fiorelli (2010); Grellet-Tinner et al. (2012)
Megaloolithidae	Titanosaurs	Embryos	Chiappe et al. (1998; 2001)
Prismatoolithidae (excluding *Preprismatoolithus*)	Troodontids and non-oviraptorosaur maniraptorans	Embryos and eggshell morphology	Varricchio et al. (1997; 2002); Zelenitsky and Therrien (2008a); Lopez-Martinez and Vicens (2012)
Spheroolithidae	Ornithschian, including *Maiasaura* and *Hypacrosaurus*	Embryos	Horner and Makela (1979); Hirsch and Quinn (1990); Horner (1999; 2000)

Supplementary Table S6. Approximate paleolatitudes of egg localities for major dinosaur ootaxa.

Abbreviations: D, Dendroolithidae; E, Elongatoolithidae; Fa, Faveoloolithidae; Me, Megaloolithidae; NA, not available; Pre, *Preprismatoolithus*; Pri, Prismatoolithidae; Sp, Spheroolithidae. Positive and negative values of paleolatitudes represent north and south latitudes, respectively.

Ootaxon	Locality	Formation	Approximate age (Ma)	Approximate paleolatitude (°)	Example of references
D	Heyuan Basin, Guangdong, China	Dongyuan	65	29.10	Fang et al. (2005)
D	Nanyang Valley near Xinye, Henan, China	Nancho	80	36.50	Manning et al. (1997); Kundrat et al. (2008)
D	Xixia Basin (or Xinping-Chimei Basin), Xixia, Henan, China	Zhaoying	85	37.85	Fang et al. (2007)
D	Xixia Basin (or Xinping-Chimei Basin), Xixia, Henan, China	Zhoumagang	90	38.60	Fang et al. (2007)
D	Taohe, Xichuan, Henan, China	Majiacun	85	37.85	Zhao and Zhao (1998)
D	Dashiqiao, Xichuan, Henan, China	Gaogou	90	38.40	Zhao and Zhao (1998)

Ootaxon	Locality	Formation	Approximate age (Ma)	Approximate paleolatitude (°)	Example of references
D	Yunxian, Hubei, China	Gaogou	90	37.70	Zhou et al. (1998)
D	Heshui, Hubei, China	Gong-An-Zhai	80	35.70	Zhao and Li (1988)
D	Western Dongting Basin, Hunan, China	Fenshuiao	80	32.80	Zeng and Zhang (1979)
D	Tiantai Basin, Zhejiang, China	Chichengshan	95	36.30	Fang et al. (1998; 2000)
D	Khermeen Tsav, Gobi, Mongolia	Barungoyot/ Nemegt	75	44.95	Watabe et al. (2010)
D	Shilyust-Ula, Gobi, Mongolia	?Barungoyot	75	45.20	Mikhailov (1994a)
D	Bayn Shire, Gobi, Mongolia	Baynshire	85	48.00	Watabe et al. (2010)
D	Abdrant Nuru, Gobi, Mongolia	?Djadokhta	70	46.60	Watabe (2004)
D	Khaichin Ula, Gobi, Mongolia	Nemegt	70	45.90	Suzuki and Watabe (2000b)
D	Yagaan Khovil, Gobi, Mongolia	?Nemegt	70	45.50	Watabe et al. (2010)
D	Shiwa Bay, Whaseong, Gyeonggi, South Korea	Shiwa	120	41.50	Lee (2003)
D	Porto das Barcas, near	Lourinha	150	26.70	Ribeiro et al. (2014)

Ootaxon	Locality	Formation	Approximate age (Ma)	Approximate paleolatitude (°)	Example of references
	Lourinha, Portugal				
E	Maoming Basin, Guangdong, China	Tongguling	80	26.20	Zhang and Huang (1999)
E	Nanxiong Basin (or Nanxiong-Shixing Basin), Guangdong, China	Dongyuan, Nanxiong, Pingling, Yuanpu, Zhenshui, and Zhutian	65	30.35	Fang et al. (2009)
E	Sanshui-Guangzhou Basin, Guangdong, China	Sanshui	85	28.10	Zhang and Huang (1999)
E	Heyuan Basin, Guangdong, China	Nanxiong and Dongyuan	65	29.10	Zhang and Huang (1999)
E	Liguanqiao Basin, Henan, China	Sigou (or Luyemiao or Hugang)	75	36.80	Liang et al. (2009)
E	Lingbao Basin, Henan, China	Sigou (or Luyemiao or Hugang)	75	38.35	Liang et al. (2009)
E	Luanchuan, Tantou Basin, Henan, China	Quipa	65	38.55	Tanaka et al. (2011)
E	Tantou Basin, Henan, China	Sigou (or Luyemiao or Hugang)	75	38.00	Liang et al. (2009)
E	Xichuan Basin, Henan,	Majiacun	85	37.70	Zhao and Zhao

Ootaxon	Locality	Formation	Approximate age (Ma)	Approximate paleolatitude (°)	Example of references
	China				(1998)
E	Xichuan Basin, Henan, China	Sigou (or Luyemiao or Hugang)	75	37.15	Liang et al. (2009)
E	Xixia Basin (or Xinping-Chimei Basin), Xixia, Henan, China	Zhaoying	85	37.85	Fang et al. (2007)
E	Xixia Basin (or Xinping-Chimei Basin), Xixia, Henan, China	Gaogou (or Zoumagang)	90	38.60	Liang et al. (2009)
E	Western Dongting Basin, Hunan, China	Fenshuiao	80	32.80	Zeng and Zhang (1979)
E	Bayan Mandahu, Inner Mongolia, China	Djadokhta	70	44.70	Dong and Currie (1996)
E	Pingxiang Basin, Jiangxi, China	Zhoutian	80	32.00	Wang et al. (2013)
E	Ganzhou, Jiangxi, China	Nanxiong	65	31.20	Cheng et al. (2008); Ji (2009)
E	Badaohao, Heishan, Liaoning, China	Shahai	110	44.00	Zhao and Zhao (1999)
E	Laiyang-Zhucheng,	Jiangjunding	85	42.85	Zhao et al. (2013)

Ootaxon	Locality	Formation	Approximate age (Ma)	Approximate paleolatitude (°)	Example of references
	Shangdong, China	Formation in the Wangshi Group			
E	Laiyang-Zhucheng, Shangdong, China	Jingangkou Formation in the Wangshi Group	75	42.40	Zhao et al. (2013)
E	Tiantai Basin, Zhejiang, China	Chichengshan	90	35.80	Wang et al. (2010)
E	Tiantai Basin, Zhejiang, China	Liangtoutang	100	36.90	Jin et al. (2007)
E	Lavariya Muwada, Kheda, Gujarat, India	Lameta	65	-19.85	Mohabey (1998)
E	Kamitaki, Tamba, Hyogo, Japan	'Lower Formation' of Sasayama Group	110	49.00	Tanaka et al. (2016)
E	Northern Sayr, Nemegt, Gobi, Mongolia	Baruungoyot	75	45.15	Fanti et al. (2012)
E	Khermeen Tsav, Gobi, Mongolia	Barungoyot/ Nemegt	75	44.95	Watabe et al. (2010)
E	Ikh-Shunkht, Gobi, Mongolia	?Barun-Goyot	75	46.60	Mikhailov (1994b)
E	Bayn Shire, Gobi, Mongolia	Baynshire	85	48.00	Watabe et al. (2010)

Ootaxon	Locality	Formation	Approximate age (Ma)	Approximate paleolatitude (°)	Example of references
E	Mongot, Gobi, Mongolia	Djadokhta	70	46.40	Suzuki and Watabe (2000b)
E	Ukhaa Tolgod, Mongolia	Djadokhta	70	45.30	Norell et al. (1994; 2001)
E	Bayn Dzak, Gobi, Mongolia	Djadokhta	70	46.40	Suzuki and Watabe (2000b)
E	Bortolgoi, Gobi, Mongolia	Djadokhta	70	46.20	Watabe et al. (2010)
E	Dzamin Khond, Gobi, Mongolia	Djadokhta	70	46.30	Suzuki and Watabe (2000b)
E	Tugrikin Shire, Gobi, Mongolia	Djadokhta	70	46.50	Watabe et al. (2010)
E	Abdrant Nuru, Gobi, Mongolia	?Djadokhta	70	46.70	Watabe (2004)
E	Udan-Sayr, Gobi, Mongolia	Djadokhta	70	46.40	Mikhailov (1994b)
E	Buylyasutuin-Khuduk, Ubur-Khangay, Mongolia	Dushi Ula (Doshuul)	120	43.10	Kurzanov and Mikhailov (1989); Mikhailov (1994b)
E	Bugin-Tsav, Gobi, Mongolia	Nemegt	70	45.50	Weishampel et al. (2008)
E	Guriliyn-Tsav, Gobi,	Nemegt	70	46.10	Mikhailov (1994b)

Ootaxon	Locality	Formation	Approximate age (Ma)	Approximate paleolatitude (°)	Example of references
	Mongolia				
E	Khaichin Ula, Gobi, Mongolia	Nemegt	70	45.90	Suzuki and Watabe (2000b)
E	Khermeen Tsav-II, Gobi, Mongolia	Nemegt	70	45.50	Watabe et al. (2010)
E	Shar Tsav, Gobi, Mongolia	Nemegt	70	46.90	Watabe et al. (2010)
E	Tsagan-Khushu, Gobi, Mongolia	Nemegt	70	45.40	Mikhailov (1994b)
E	Yagaan Khovil, Gobi, Mongolia	?Nemegt	70	46.30	Watabe et al. (2010)
E	Busan, South Korea	Dadaepo	75	41.90	Paik et al. (2012)
E	Tongyeong, Gyeongsang, South Korea	Goseong	75	41.60	Kim et al. (2011)
E	Aphae-do, Shinan-gun, Jeollanam-do, South Korea	Gyeongsang Basin	80	41.00	Huh et al. (2014)
E	Shiwa Bay, Whaseong, Gyeonggi, South Korea	Shiwa	120	41.40	Lee (2003)
E	Teruel, Spain	Blesa	130	25.60	Canudo et al. (2010)
E	Bonneville County, Idaho, USA	Wayan	109.0–99.7	43.30	Krumenacker et al. (2016)

Ootaxon	Locality	Formation	Approximate age (Ma)	Approximate paleolatitude (°)	Example of references
E	Pancake and Fish Creek Ranges, Nevada, USA	Newark Canyon	125.5– 99.7	41.90	Bonde et al. (2015)
E	Castle Dale, Emery County, Utah	Cedar Mountain	105.3– 94.3	39.70	Zelenitsky et al. (2000)
E	Section 4 Site 7, Kitchen Locality, Sauropod Locality, Sauropod One Egg, Pond Locality, Lizard Locality, and North Horn Mountain, Emery County, Utah, USA	North Horn	70.6–66.0	47.20	Bray (1999)
E	UCM Loc. #78203, Emery County, Utah, USA	Dakota	105.3–94.3	39.70	Zelenitsky et al. (2000)
Fa	Qinglongshan, Yunxian, Hubei, China	Gaogou	90	37.70	Zhou et al. (1998)
Fa	Pingxiang Basin, Jiagxi, China	Zhoutian	80	32.00	Zou et al. (2013)
Fa	Tiantai, Zhejiang, China	Laijia	95	36.35	Zhang (2010)
Fa	Algui Ulan Tsav, Gobi, Mongolia	NA	115	44.25	Watabe et al. (2010)
Fa	Ologoy-Ulan-Tsav, Gobi,	Barungoyot	75	47.10	Sochava (1969);

Ootaxon	Locality	Formation	Approximate age (Ma)	Approximate paleolatitude (°)	Example of references
	Mongolia				Mikhailov (1994a)
Fa	Bosung County, Chullanam-do Province, South Korea	Seonso	80	41.00	Huh and Zelenitsky (2002)
Fa	Coast of Sinsudo Island, Samcheonpo, South Korea	Haman	100	43.20	Paik et al. (2012)
Fa	Hanyom, Sihwa Basin, South Korea	Sihwa	120	41.50	Kim et al. (2009)
Fa	Ojo de Agua, Rio Negro, Argentina	Allen	75	-44.05	Salgado et al. (2007)
Fa	Salitral de Santa Rosa-Salinas de Trapalco, Rio Negro, Argentina	Allen	75	-44.85	Salgado et al. (2007)
Fa	Sanagasta, La Rioja, Argentina	Los Llanos	125	-26.10	Grellet-Tinner and Fiorelli (2010); Fiorelli et al. (2012)
Fa	Colonia, 12a. Police Section, stream Miguelete Tala, Uruguay	Asencio	95	-38.75	Mones (1980)
Fa	Quebracho, Paysandu, Uruguay	Guichon	100	-36.10	Soto et al. (2012)

Ootaxon	Locality	Formation	Approximate age (Ma)	Approximate paleolatitude (°)	Example of references
Me	Douar Lgara, Tendrara High Plateaus, Morocco	Tendrara red beds	65	19.15	Chassagne-Manoukian et al. (2013)
Me	Oukdiksou syncline, Morocco	Irbzer	65	19.95	Garcia et al. (2003)
Me	TZ-07, Mbeya District, Tanzania	Galula Formation of Red Sandstone Group	105	-28.70	Gottfried et al. (2004)
Me	Rahioli and Balasinor region, Kheda, Gujarat, India	Lameta	65	-19.85	Srivastava et al. (1986)
Me	Anjar, Kachchh, Gujarat, India	Lameta	65	-18.30	Khosla and Sahni (1995)
Me	Jhabua, Madhya Pradesh, India	Lameta	65	-20.40	Khosla and Sahni (1995)
Me	Khempur, Kheda, Gujarat, India	Lameta	65	-18.30	Khosla and Sahni (1995)
Me	Dohad, Panchmahals, Gujarat, India	Lameta	65	-20.25	Mohabey and Mathur (1989)
Me	Dhar, Madhya Pradesh,	Lameta	65	-20.80	Khosla and Sahni

Ootaxon	Locality	Formation	Approximate age (Ma)	Approximate paleolatitude (°)	Example of references
	India				(1995)
Me	Padiyal, Madhya Pradesh, India	Lameta	65	-20.95	Khosla and Sahni (1995)
Me	Jabalpur, Madhya Pradesh, India	Lameta	65	-21.55	Khosla and Sahni (1995)
Me	Amravati, Maharashtra, India	Lameta	65	-23.10	Srivastava and Mankar (2015)
Me	Chandrapur, Maharashtra, India	Lameta	65	-24.50	Mohabey (1996; 1998)
Me	Durlston Bay, Swanage, Dorset, England	Chery Freshwater Member of the Lulworth Formation	140	33.10	Ensom (1996)
Me	Aix-en-Provence region, France	NA	75	33.20	Vianey-Liaud et al. (1994)
Me	Aix-en-Provence region, France	NA	65	32.45	Vianey-Liaud et al. (1994)
Me	Aude region, France	Red Marls of Maurine	65	32.00	Fondevilla et al. (2016)
Me	Hateg Basin, Romania	Densus-Ciula	70.6–66.0	32.70	Grigorescu et al. (1994; 2010)

Ootaxon	Locality	Formation	Approximate age (Ma)	Approximate paleolatitude (°)	Example of references
Me	Southern Pyrenees region (e.g., Coll de Nargo and Tremp), Spain	Aren	70	30.00	Sanz et al. (1995); Diaz-Molina et al. (2007)
Me	Southern Pyrenees region (e.g., Coll de Nargo and Tremp), Spain	Tremp	65	29.65	Vianey-Liaud and Lopez-Martinez (1997)
Me	Auca Mahuevo, Neuquen Province, Patagonia, Argentina	Anacleto	80	-42.30	Chiappe et al. (1998)
Me	Arrigada I, Salitral Ojo de Agua, Rio Negro, Argentina	Allen	75	-44.05	Salgado et al. (2007)
Me	Salitral de Santa Rosa-Salinas de Trapalco, Rio Negro, Argentina	Allen	75	-44.85	Salgado et al. (2007)
Me	Bajo de Arriagada, Patagonia, Rio Negro, Argentina	Allen	75	-44.00	Genise and Sarzetti (2011)
Me	Neuquen, Neuquen, Patagonia, Argentina	Rio Colorado	85	-43.85	Calvo et al. (1997)
Me	Sierra de Los Llanos, La	Los Llanos	80	-35.50	Hechenleitner et al.

Ootaxon	Locality	Formation	Approximate age (Ma)	Approximate paleolatitude (°)	Example of references
	Rioja, Argentina				(2016); Basilici et al. (2017)
Me	Baruru Basin, Marilia County, San Paulo State, Brazil	Marilia	65	-29.95	Ribeiro (2002)
Me	Laguna Umayo, Bagua Basin, Peru	Fundo El Triunfo	75	-21.10	Vianey-Liaud et al. (1997; 2003)
Pre	Casal da Rola, near Lourinha, Portugal	Lourinha	150	28.60	Ribeiro et al. (2014)
Pre	Pai Mogo, Lourinha, Portugal	Lourihna	150	28.40	Mateus et al. (1997); Antunes et al. (1998)
Pre	Freemont County, Colorado, USA	Morrison	155.7–145.5	34.00	Zelenitsky (2004)
Pre	Delta and Montrose County, Colorado, USA	Morrison	155.7–150.8	33.70	Hirsch (1994)
Pre	Cleveland-Lloyd Dinosaur Quarry, Emery County, Utah, USA	Morrison	155.7–150.8	34.90	Hirsch et al. (1989); Hirsch (1994)
Pre	Fox Mesa, Wyoming, USA	Morrison	155.7–145.5	40.30	Carrano et al. (2013)
Pri	Oukdiksou syncline,	Irbzer	65	19.95	Garcia et al. (2003)

Ootaxon	Locality	Formation	Approximate age (Ma)	Approximate paleolatitude (°)	Example of references
	Morocco				
Pri	Heyuan Basin, Guangdong, China	Dongyuan	65	29.10	Lü et al. (2006); Tanaka et al. (2012)
Pri	Xixia Basin (or Xinping-Chimei Basin), Xixia, Henan, China	Majiacun (or Zhaoying)	85	37.85	Liang et al. (2009)
Pri	Xixia Basin (or Xinping-Chimei Basin), Xixia, Henan, China	Gaogou	90	38.60	Wang and Zhou (1995)
Pri	Hejiagou, Yunxian, Hubei, China	Gaogou	90	37.70	Zhou et al. (1998)
Pri	Bayan Mandahu, Urad Houqi, Inner Mongolia, China	Djadokhta	70	44.70	Zhao and Li (1993)
Pri	Ganzhou, Jiangxi, China	Nanxiong	65	31.20	Tanaka pers. obs. at Ganzhou Museum, Jiangxi Province, China
Pri	Tiantai Basin, Zhejiang, China	Chichengshan and Laijia	95	36.35	Fang et al. (2000; 2003); Wang et al.

Ootaxon	Locality	Formation	Approximate age (Ma)	Approximate paleolatitude (°)	Example of references
					(2011)
Pri	Tiantai Basin, Zhejiang, China	Liangtoutang	100	36.90	Varricchio et al. (2015)
Pri	Kamitaki, Tamba, Hyogo, Japan	'Lower Formation' of Sasayama Group	110	49.00	Tanaka et al. (2016)
Pri	Khermin-Tsav, Gobi, Mongolia	Barun-Goyot	75	45.25	Mikhailov (1994b)
Pri	Baga-Tariach, Gobi, Mongolia	Dzhadokhta	70	48.90	Mikhailov (1994b)
Pri	Tugrikin Shire, Gobi, Mongolia	Djadokhta	70	46.50	Watabe et al. (2010)
Pri	Zhinst Tolgoi, Udyn Sayr, Gobi, Mongolia	Djadokhta	70	46.40	Suzuki and Watabe (2000a)
Pri	Bayn Dzak, Gobi, Mongolia	Djadokhta	70	46.50	Watabe et al. (2010)
Pri	Khashaat, Gobi, Mongolia	Dzhadokhta	70	46.50	Sabath (1991)
Pri	Altan Ula IV, Gobi, Mongolia	Nemegt	70	45.50	Sabath (1991)
Pri	Kakanaut, Koryak Upland, Chukotka Autonomous Region, Russia	Kakanaut	65	76.70	Godefroit et al. (2009)

Ootaxon	Locality	Formation	Approximate age (Ma)	Approximate paleolatitude (°)	Example of references
Pri	Herault region, France	Villeveyrac-Meze Basin	75	32.85	Garcia et al. (2000)
Pri	Aix-en-Provence region, France	NA	75	32.95	Garcia et al. (2000)
Pri	Teruel, Spain	Belsa	130	25.60	Canudo et al. (2010); Moreno-Azanza et al. (2014b)
Pri	Southern Pyrenees region (e.g., Coll de Nargo and Tremp), Spain	Tremp	65	29.65	Vianey-Liaud and Lopez-Martinez (1997)
Pri	Southern Pyrenees region (e.g., Coll de Nargo and Tremp), Spain	Aren	70	30.00	Lopez-Martinez and Vicens (2012)
Pri	Dinosaur Provincial Park, Alberta	Dinosaur Park	75	57.05	Zelenitsky and Sloboda (2005)
Pri	Devil's Coulee, Alberta, Canada	Oldman	75	56.05	Zelenitsky and Hills (1996)
Pri	Todd Creek area, Alberta Canada	Willow Creek	65	55.65	Zelenitsky et al. (2017b)
Pri	Verdigris Coulee, Alberta,	Milk River	85	54.35	Zelenitsky et al.

Ootaxon	Locality	Formation	Approximate age (Ma)	Approximate paleolatitude (°)	Example of references
	Canada				(2017a)
Pri	Near Choteau, Teton County, Montana; Glacier County, Montana, USA	Two Medicine	75	54.55	Varricchio et al. (2002)
Pri	First Find Microsite, Sevenmile Hill outcrops, Teton County, Montana, USA	Two Medicine	80	54.00	Jackson and Varrichhio (2010)
Pri	Hill County, Montana, USA	Judith River	75	54.75	Jackson et al. (2010)
Pri	Bone Hill Locality, Red Rock Locality, Teton County, Montana; Glacier County, Montana, USA	Two Medicine	85	53.10	Hirsch and Quinn (1990)
Pri	NMMNH L-4010, Bisti/De-na-zin Wilderness area, San Juan Basin, New Mexico, USA	Fruitland	84.9–70.6	43.60	Tanaka et al. (2011)
Pri	Lizard and Sauropod localities, Emery County, Utah, USA	North Horn	70.6–66.0	47.20	Bray (1999)

Ootaxon	Locality	Formation	Approximate age (Ma)	Approximate paleolatitude (°)	Example of references
Pri	UCM L79059, Mudbank Site 1.2, Emery County, Utah, USA	North Horn	70.6–66.0	46.10	Bray (1999)
Pri	El Pantano, Coahuila, Mexico	Cerro del Pueblo	84.9–70.6	31.80	Aguillon-Martinez et al. (2004)
Sp	Heyuan Bsin, Guangdong, China	Nanxiong	65	29.10	Zhang and Huang (1999)
Sp	Maoming Basin, Guangdong, China	Tongguling	80	25.65	Zhang and Huang (1999)
Sp	Nanxiong Basin (or Nanxiong-Shixing Basin), Guangdong, China	Nanxiong and Pingling	65	30.35	Zhang and Huang (1999)
Sp	Sanshui-Guangzhou Basin, Guangdong, China	Sanshui	90	28.80	Zhang and Huang (1999)
Sp	Sanshui-Guangzhou Basin, Guangdong, China	Baihedong Group	125	26.25	Zhang and Huang (1999)
Sp	Loushan Basin, Henan, China	Majiacun (or Zhaoying)	85	37.25	Liang et al. (2009)
Sp	Wulichuan Basin, Henan, China	Majiacun (or Zhaoying)	85	38.35	Liang et al. (2009)

Ootaxon	Locality	Formation	Approximate age (Ma)	Approximate paleolatitude (°)	Example of references
Sp	Wulichuan Basin, Henan, China	Gaogou (or Zoumagang)	90	39.00	Liang et al. (2009)
Sp	Xiaguan Basin, Henan, China	Majiacun (or Zhaoying)	85	37.90	Liang et al. (2009)
Sp	Xichuan Basin, Henan, China	Majiacun (or Zhaoying)	85	37.70	Liang et al. (2009)
Sp	Xichuan Basin, Henan, China	Gaogou (or Zoumagang)	90	38.40	Liang et al. (2009)
Sp	Xixia Basin (or Xinping-Chimei Basin), Xixia, Henan, China	Majiacun (or Zhaoying)	85	37.85	Liang et al. (2009)
Sp	Xixia Basin (or Xinping-Chimei Basin), Xixia, Henan, China	Gaogou (or Zoumagang)	90	38.60	Liang et al. (2009)
Sp	Xixia Basin (or Xinping-Chimei Basin), Xixia, Henan, China	Sigou (or Luyemiao or Hugang)	75	37.25	Liang et al. (2009)
Sp	Yangji Basin, Henan, China	Majiacun (or Zhaoying)	85	38.10	Liang et al. (2009)
Sp	Qinglongshan, Yunxian,	Gaogou	90	37.70	Zhou et al. (1998)

Ootaxon	Locality	Formation	Approximate age (Ma)	Approximate paleolatitude (°)	Example of references
	Hubei, China				
Sp	Changtu, Liaoning, China	Quantou	110	45.40	Liu et al. (2013)
Sp	Laiyang-Zhucheng, Shangdong, China	Jiangjunding Formation in the Wangshi Group	85	42.85	Young (1965); Zhao and Jiang (1974)
Sp	Laiyang-Zhucheng, Shangdong, China	Jingangkou Formation in the Wangshi Group	75	41.25	Zhao et al. (2013)
Sp	Tiantai Basin, Zhejiang, China	Chichengshan and Laijia	95	36.35	Fang et al. (2003; 2000); Wang et al. (2011)
Sp	Kholdoda and Chandrapur, Maharashtra, India	Lameta	65	-22.45	Mohabey (1996; 2001)
Sp	Kamitaki, Tamba, Hyogo, Japan	'Lower Formation' of Sasayama Group	110	49.00	Tanaka et al. (2016)
Sp	Shiluut Ula, Gobi, Mongolia	Barungoyot	75	45.20	Watabe et al. (2010)
Sp	Abdrant Nuru, Gobi, Mongolia	?Djadokhta	70	46.60	Watabe (2004)
Sp	Baga-Tariach, Gobi,	Djadokhta	70	48.90	Mikhailov (1994a)

Ootaxon	Locality	Formation	Approximate age (Ma)	Approximate paleolatitude (°)	Example of references
	Mongolia				
Sp	Bortolgoi, Gobi, Mongolia	Djadokhta	70	46.20	Watabe et al. (2010)
Sp	Dzamin Khond, Gobi, Mongolia	Djadokhta	70	46.30	Watabe et al. (2010)
Sp	Dragon's Tomb' dinosaur locality, Gobi, Mongolia	Nemegt	70	45.40	Dewaele et al. (2015)
Sp	Shar Tsav, Gobi, Mongolia	Nemegt	70	46.80	Watabe et al. (2010)
Sp	Yagaan Khovil, Gobi, Mongolia	?Nemegt	70	46.20	Watabe et al. (2010)
Sp	Kakanaut, Koryak Upland, Chukotka Autonomous Region, Russia	Kakanaut	65	76.70	Godefroit et al. (2009)
Sp	Bosung, Chullanam-do, South Korea	Seonso	80	41.00	Huh and Zelenitsky (2002)
Sp	Goseong, South Korea	Goseong	75	41.60	Paik et al. (2012)
Sp	Teruel, Spain	Blesa	130	25.60	Moreno-Azanza et al. (2014a)
Sp	Teruel, Spain	Camarillas, Marambel, and Upper El Castellar	130	25.50	Moreno-Azanza et al. (2014a)

Ootaxon	Locality	Formation	Approximate age (Ma)	Approximate paleolatitude (°)	Example of references
Sp	Southern Pyrenees region (e.g., Coll de Nargo and Tremp), Spain	Tremp	65	29.65	Sellés et al. (2014)
Sp	Dinosaur Provincial Park, Alberta	Dinosaur Park and Oldman	75	57.05	Zelenitsky and Sloboda (2005)
Sp	Devil's Coulee, Alberta, Canada	Oldman	75	56.05	Zelenitsky and Hills (1997)
Sp	Knight's Ranch, Alberta, Canada	Oldman	75	55.95	Zelenitsky and Hills (1997)
Sp	Whiskey Gap area and Todd Creek area, Alberta Canada	Willow Creek	65	55.65	Zelenitsky et al. (2017b)
Sp	Verdigris Coulee, Alberta, Canada	Milk River	85	54.35	Zelenitsky et al. (2017a)
Sp	Near Choteau, Teton County, Montana, USA	Two Medicine	75	54.55	Horner and Makela (1979)
Sp	Sevenmile Hill, Teton County, Montana, USA	Two Medicine	80	54.00	Jackson and Varrichhio (2010)
Sp	Blacktail Creek, Glacier County, Montana, USA	Two Medicine	75	55.35	Horner and Currie (1994)
Sp	One Place Locality, Red	Two Medicine	85	53.10	Hirsch and Quinn

Ootaxon	Locality	Formation	Approximate age (Ma)	Approximate paleolatitude (°)	Example of references
	Rock Locality, Teton County, Montana; Glacier County, Montana				(1990)
Sp	Lizard Locality, Emery County, Utah, USA	North Horn	70.6–66.0	47.20	Bray (1999)
Sp	El Pantano, Coahuila, Mexico	Cerro del Pueblo	84.9–70.6	31.80	Aguillon-Martinez et al. (2004)

References

Agnolin, F.L., Powell, J.E., Novas, F.E. and Kundrat, M., 2012. New alvarezsaurid (Dinosauria, Theropoda) from uppermost Cretaceous of north-western Patagonia with associated eggs. Cretaceous Research 35: 33-56.

Aguillon-Martinez, M., Zelenitsky, D.K., Brinkman, D. and Eberth, D., 2004. Eggshell fragments from the Uppermost Cerro del Pueblo Formation (Upper Cretaceous; Mexico). Journal of Vertebrate Paleontology 24: 33A.

Allsteadt, J., 1994. Nesting ecology of *Caiman crocodilus* in Cano Negro, Costa Rica. Journal of Herpetology 28: 12-19.

Antunes, M.T., Taquet, P.V. and Ribeiro, V., 1998. Upper Jurassic dinosaur and crocodile eggs from Pai Mogo nesting site (Lourinha - Portugal). Memorias da Academia das Ciencias de Lisboa 37: 83-99.

Araujo, R., Castanhinha, R., Martins, R.M.S., Mateus, O., Hendrickx, C., Beckmann, F., Schell, N. and Alves, L.C., 2013. Filling the gaps of dinosaur eggshell phylogeny: Late Jurassic theropod clutch with embryos from Portugal. Scientific Reports 3: 10.1038/Srep01924.

Argeloo, M. and Dekker, R.W.R.J., 1996. Exploitation of megapode eggs in Indonesia: the role of traditional methods in the

conservation of megapodes. Oryx 30: 59-64.

Baker, G.C., 1999. Temporal and spatial patterns of laying in the Moluccan megapode *Eulipoa wallacei* (G.R. Gray). Zoologische Verhandelingen 327: 53-60.

Baker, G.C. and Dekker, R.W.R.J., 2000. Lunar synchrony in the reproduction of the Moluccan Megapode *Megapodius wallacei*. Ibis 142: 382-388.

Basilici, G., Hechenleitner, E.M., Fiorelli, L.E., Dal Bo, P.F. and Mountney, N.P. 2017. Preservation of titanosaur egg clutches in Upper Cretaceous cumulative palaeosols (Los Llanos Formation, La Rioja, Argentina). Palaeogeography, Palaeoclimatology, Palaeoecology 482: 83-102.

Beetschen, J.-C., Dughi, R. and Sirugue, F., 1977. Sur la presence de coquilles d'œufs d'Oiseaux dans le cretace superieur des Corbieres occidentales. Comptes Rendus de l'Académie des Sciences-Series II: A-Earth and Planetary Sciences 284: 2491-2494.

Bezuijen, M.R., Cox, J.H., Thorbjarnarson, J.B., Phothitay, C., Hedemark, M. and Rasphone, A., 2013. Status of Siamese Crocodile (*Crocodylus siamensis*) Schneider, 1801 (Reptilia: Crocodylia) in Laos. Journal of Herpetology 47: 41-65.

Bolhm, T., 1982. Husbandry of Orinoco crocodiles (*Crocodylus intermedius*) in Venezuela. Crocodiles: Proceedings of the 5th Working Meeting of the Crocodile Specialist Group, IUCN–The World Conservation Union, Gland, Switzerland: 267-285.

Bonde, J.W., Hilton, R.P., Jackson, F.D. and Druschke, P.A., 2015. Fauna of the Newark Canyon Formation (Lower Cretaceous), east-central Nevada. New Concepts and Discoveries 1-2: 721-732.

Botfalvai, G., Csiki-Sava, Z., Grigorescu, D. and Vasile, S., 2017. Taphonomical and palaeoecological investigation of the Late Cretaceous (Maastrichtian) Tuştea vertebrate assemblage (Romania: Haţeg Basin) - insight into a unique dinosaur nesting locality. Palaeogeography, Palaeoclimatology, Palaeoecology 468: 228-262.

Bowen, J., 1996. Notes on the Vanuatu Megapode *Megapodius layardi* on Ambrym, Vanuatu. Bird Conservation International 6: 401-408.

Bravo, A.M. and Gaete, R., 2015. Titanosaur eggshells from the Tremp Formation (Upper Cretaceous, Southern Pyrenees, Spain). Historical Biology 27: 1079-1089.

Bray, E.S., 1999. Eggs and eggshell from the Upper Cretaceous North Horn Formation, central Utah. In: D.D. Gillette (Editor), Vertebrate Paleontology in Utah. Geological Survey Miscellaneous Publication 99-1, Utah, pp. 361-376.

Bray, E.S. and Lucas, S.G., 1997. Theropod dinosaur eggshell from the Upper Jurassic of New Mexico. New Mexico Museum of Natural History and Science Bulletin 11: 41-43.

Bregulla, H.L., 1992. Birds of Vanuatu. Anthony Nelson, Shropshire, UK, 329 pp.

Brown, B. and Schlaikjer, E.M., 1940. The structure and relationships of *Protoceratops*. Transactions of the New York Academy of Sciences 2: 99-100.

Bustard, H.R., 1980. Clutch size, incubation and hatching success of Gharial [*Gavialis gangeticus* (Gmelin)] eggs from Narayani River, Nepal, 1976-1978. Journal of Bombay Natural History Society 77: 100-105.

Calvo, J.O., Engelland, S., Heredia, S.E. and Salgado, L., 1997. First record of dinosaur eggshells (?Sauropoda - Megaloolithidae) from Neuquen, Patagonia, Argentina. Gaia 14: 23-32.

Canudo, J.I., Gasca, J.M., Aurell, M., Badiola, A., Blain, H.A., Cruzado-Caballero, P., Gomez-Fernandez, D., Moreno-Azanza, M., Parrilla, J., Rabal-Garces, R. and Ruiz-Omenaca, J.I., 2010. La Cantalera: an exceptional window onto the vertebrate biodiversity of the Hauterivian-Barremian transition in the Iberian Peninsula. Journal of Iberian Geology 36: 205-224.

Carrano, M., Mateus, O. and Mitchell, J., 2013. First definitive association between embryonic *Allosaurus* bones and *Prismatoolithus* eggs in the Morrison Formation (Upper Jurassic, Wyoming, USA). Journal of Vertabrate Paleontology 33. Conference abstracts: 101.

Chabreck, R.H., 1973. Temperature variation in nests of the American Alligator. Herpetologica 29: 48-51.

Chabreck, R.H., 1975. Moisture variation in nests of the American Alligator (*Alligator mississippiensis*). Herpetologica 31: 385-389.

Charruau, P., 2012. Microclimate of American Crocodile nests in Banco Chinchorro biosphere reserve, Mexico: effect on incubation

length, embryos survival and hatchlings sex. Journal of Thermal Biology 37: 6-14.

Chassagne-Manoukian, M., Haddoumi, H., Cappetta, H., Charriere, A., Feist, M., Tabuce, R. and Vianey-Liaud, M., 2013. Dating the "red beds" of the Eastern Moroccan High Plateaus: evidence from late Late Cretaceous charophytes and dinosaur eggshells. Geobios 46: 371-379.

Cheng, Y.N., Jui, Q., Wu, X.C. and Shou, H.Y., 2008. Oviraptorosaurian eggs (Dinosauria) with embryonic skeletons discovered for the first time in China. Acta Geologica Sinica 82: 1089-1094.

Chiappe, L.M., Coria, R.A., Dingus, L., Jackson, F., Chinsamy, A. and Fox, M., 1998. Sauropod dinosaur embryos from the late Cretaceous of Patagonia. Nature 396: 258-261.

Chiappe, L.M., Dingus, L., Jackson, F.D., Grellet-Tinner, G., Coria, R., Loope, D., Clarke, J. and Garrido, A., 2001. Sauropod eggs and embryos from the Late Cretaceous of Patagonia. In: A.M. Bravo and T. Reyes (Editors), First International Symposium on Dinosaur Eggs and Babies Extended Abstracts, Catalonia, Spain, pp. 23-29.

Chiappe, L.M., Schmitt, J.G., Jackson, F.D., Garrido, A., Dingus, L. and Grellet-Tinner, G., 2004. Nest structure for sauropods: sedimentary criteria for recognition of dinosaur nesting traces. Palaios 19: 89-95.

Chow, M.M., 1951. Notes on the Late Cretaceous dinosaurian remains and the fossil eggs from Laiyang Shantung. Bulletin of the Geological Society of China 31: 89-96.

Chow, M.M., 1954. On the microstructure of the fossil egg-shells from Laiyang, Shantung. Acta Palaeontologica Sinica 2: 389-394.

Cintra, R., 1988. Nesting ecology of the Paraguayan Caiman (*Caiman yacare*) in the Brazilian Pantanal. Journal of Herpetology 22: 219-222.

Clark, J.M., Norell, M.A. and Chiappe, L.M., 1999. An oviraptorid skeleton from the Late Cretaceous of Ukhaa Tolgod, Mongolia, preserved in an avianlike brooding position over an oviraptorid nest. American Museum Novitates 3265: 1-35.

Codrea, V., Smith, T., Dica, P., Folie, A., Garcia, G., Godefroit, P. and Van Itterbeeck, J., 2002. Dinosaur egg nests, mammals and other vertebrates from a new Maastrichtian site of the Hateg Basin (Romania). Comptes Rendus Palevol 1: 173-180.

Cott, H.B., 1961. Scientific results of an inquiry into the ecology and economic status of the Nile Crocodile (*Crocodylus niloticus*) in Uganda and Northern Rhodesia. The Transactions of the Zoological Society of London 29: 211-356.

Cousin, R., Breton, G., Fournier, R. and Watte, J.-P., 1989. Dinosaur egg-laying and nesting: the case of an Upper Maastrichtian site at Rennes-le-Chateau (Aude, France). Historical Biology 2: 157-167.

Crawshaw, P.G. and Schaller, G.B., 1980. Nesting of Paraguayan Caiman (*Caiman yacare*) in Brazil. Papeis Avulsos de Zoologia 33: 283-292.

Crome, F.H.J. and Brown, H.E., 1979. Notes on social organization and breeding of the Orange-footed Scrubfowl *Megapodius reinwardt*. Emu 79: 111-119.

Dekker, R.W.R.J., 1988. Notes on ground temperatures at nesting sites of the Maleo *Macrocephalon maleo* (Megapodiidae). Emu 88: 124-127.

Dekker, R.W.R.J., Argeloo, M. and Jepson, P., 1995. Notes on the Moluccan Megapode *Eulipoa wallacei* (G.R. Gray, 1860) following the rediscovery of two major nesting grounds. Zoologische Mededelingen 69: 251-260.

Dewaele, L., Tsogtbaatar, K., Barsbold, R., Garcia, G., Stein, K., Escuillie, F. and Godefroit, P., 2015. Perinatal Specimens of *Saurolophus angustirostris* (Dinosauria: Hadrosauridae), from the Upper Cretaceous of Mongolia. PLoS One 10: 10.1371/journal.pone.0138806.

Diaz-Molina, M., Kalin, O., Benito, M.I., Lopez-Martinez, N. and Vicens, E., 2007. Depositional setting and early diagenesis of the dinosaur eggshell-bearing Aren Fm at Bastus, Late Campanian, south-central Pyrenees. Sedimentary Geology 199: 205-221.

Dong, Z.M. and Currie, P.J., 1996. On the discovery of an oviraptorid skeleton on a nest of eggs at Bayan Mandahu, Inner Mongolia, People's Republic of China. Canadian Journal of Earth Sciences 33: 631-636.

Ensom, P.C., 1996. Reptile eggshell from the Purbeck Limestone Group of Dorset, Southern England. Proceedings of Dorset Natural History and Archaeological Society 118: 79-83.

Erickson, G.M., Zelenitsky, D.K., Kay, D.I. and Norell, M.A., 2017. Dinosaur incubation periods directly determined from growth-line

counts in embryonic teeth show reptilian-grade development. Proceedings of the National Academy of Sciences of the United States of America 114: 540-545.

Espinosa-Blanco, A.S., Seijas, A.E. and Hernández, O., 2013. Egg collection as a conservation tool of Orinoco Crocodile (*Crocodylus intermedius*) in the Cojedes River System, Venezuela. Interciencia 38: 358.

Faccio, G., 1994. Dinosaurian eggs from the Upper Cretaceous of Uruguay. In: K. Carpenter, K.F. Hirsch and J.R. Horner (Editors), Dinosaur Eggs and Babies. Cambridge University Press, Cambridge, UK, pp. 47-55.

Falanruw, M.V.C., 1975. Distribution of the Micronesian Megapode *Megapodius laperouse* in the Northern Mariana Islands. Micronesica 11: 149-150.

Fang, X., Lu, L., Cheng, Z., Zou, Y., Pang, Q., Wang, Y., Chen, K., Yin, Z., Wang, X., Liu, J., Xie, H. and Jin, Y., 1998. On the Cretaceous Fossil Eggs of Xixia County, Henan Province. Geological Publishing House, Beijing, China, 125 pp.

Fang, X., Lu, L., Jiang, Y. and Yang, L., 2003. Cretaceous fossil eggs from the Tiantai basin of Zhejiang, with a discussion on the extinction of dinosaurs. Geological Bulletin of China 22: 512-520.

Fang, X., Wang, Y. and Jiang, Y., 2000. On the Late Cretaceous fossil eggs of Tiantai, Zhejiang. Geological Review 46: 107-112.

Fang, X., Zhang, Z., Pang, Q., Li, P.X., Han, Y., Xie, H., Yan, R., Pang, F., Lu, J. and Cheng, Z., 2007. Cretaceous strata and egg fossils in Xixia, Henan Province. Acta Geologica Sinica 28: 123-142.

Fang, X., Zhang, Z., Zhang, X., Lu, L., Han, Y. and Li, P., 2005. Fossil eggs from the Heyuan Basin, east-central Guangdong, China. Geological Bulletin of China 24: 682-686.

Fang, X.S., Li, P.X., Zhang, Z.J., Zhang, X.Q., Lin, Y.L., Guo, S.B., Cheng, Y.M., Li, Z.Y., Zhang, X.J. and Cheng, Z.W., 2009. Cretaceous strata in Nanxiong Basin of Guangdong and the evolution from the dinosaur egg to the bird egg. Acta Geoscientica Sinica 30: 167-186.

Fanti, F., Currie, P.J. and Badamgarav, D., 2012. New specimens of *Nemegtomaia* from the Baruungoyot and Nemegt formations (Late Cretaceous) of Mongolia. PLoS One 7: 10.1371/journal.pone.0031330.

Fiorelli, L.E., Grellet-Tinner, G., Alasino, P.H. and Arganaraz, E., 2012. The geology and palaeoecology of the newly discovered Cretaceous neosauropod hydrothermal nesting site in Sanagasta (Los Llanos Formation), La Rioja, northwest Argentina. Cretaceous Research 35: 94-117.

Fondevilla, V., Dinares-Turell, J., Vila, B., Le Loeuff, J., Estrada, R., Oms, O. and Galobart, A., 2016. Magnetostratigraphy of the Maastrichtian continental record in the Upper Aude Valley (northern Pyrenees, France): Placing age constraints on the succession of dinosaur-bearing sites. Cretaceous Research 57: 457-472.

Friedlander, B., 1899. Über die Nestlöcher des *Megapodius pritchardi* auf der Insel Niuafu. Ornithologische Monatsberichte 7: 37-40.

Frith, H.J., 1956. Breeding habits in the family Megapodiidae. Ibis 98: 620-640.

Frith, H.J., 1959. Breeding of the Mallee Fowl, *Leipoa ocellata* Gould (Megapodiidae). Wildlife Research 4: 31-60.

Garcia, G., 2000. New data on the fossil eggshell diversity from the lower Maastrichtian of southern France. Geobios 33: 113-126.

Garcia, G., Feist, M., Cabot, A., Valentin, X. and Vianey-Liaud, M., 2000. New *Prismatoolithus* oospecies and other Upper Cretaceous dinosaur eggs from the Villeveyrac-Meze Basin (Herault, France). Bulletin de la Societe Geologique de France 171: 283-289.

Garcia, G., Tabuce, R., Cappetta, H., Marandat, B., Bentaleb, I., Benabdallah, A. and Vianey-Liaud, M., 2003. First record of dinosaur eggshells and teeth from the North-West African Maastrichtian (Morocco). Palaeovertebrata 32: 59-69.

Garcia, G. and Vianey-Liaud, M., 2001. Dinosaur eggshells as biochronological markers in Upper Cretaceous continental deposits. Palaeogeography Palaeoclimatology Palaeoecology 169: 153-164.

Genise, J.F. and Sarzetti, L.C., 2011. Fossil cocoons associated with a dinosaur egg from Patagonia, Argentina. Palaeontology 54: 815-823.

Gilliard, E.T. and LeCroy, M., 1970. Notes on birds from the Tamrau Mountains, New Guinea. American Museum Novitates 2420: 1-28.

Glass, P.O., Reichel, J.D., Villagomez, E.C., Taisacan, E. and Aldan, D., 1988. Micronesian megapode surveys and research. Five Year Progress Report 1983-1987 (Pittman-Robertson Federal Aid in Wildlife Restoration Program), pp. 131-153.

Godefroit, P., Golovneva, L., Shchepetov, S., Garcia, G. and Alekseev, P., 2009. The last polar dinosaurs: high diversity of latest Cretaceous arctic dinosaurs in Russia. Naturwissenschaften 96: 495-501.

Goodwin, T.M. and Marion, W.R., 1978. Aspects of nesting ecology of American alligators (*Alligator mississippiensis*) in north-central Florida. Herpetologica 34: 43-47.

Goth, A. and Vogel, U., 1996-1997. Egg laying and incubation of the Polynesian Megapodes. Annual Review of the World Pheasant Association: 43-54.

Gottfried, M.D., O'Connor, P.M., Jackson, R.D., Roberts, E.M. and Cham, R., 2004. Dinosaur eggshell from the red sandstone group of Tanzania. Journal of Vertebrate Paleontology 24: 494-497.

Grellet-Tinner, G., Chiappe, L., Norell, M. and Bottjer, D., 2006. Dinosaur eggs and nesting behaviors: a paleobiological investigation. Palaeogeography, Palaeoclimatology, Palaeoecology 232: 294-321.

Grellet-Tinner, G., Chiappe, L.M. and Coria, R., 2004. Eggs of titanosaurid sauropods from the Upper Cretaceous of Auca Mahuevo (Argentina). Canadian Journal of Earth Sciences 41: 949-960.

Grellet-Tinner, G. and Fiorelli, L.E., 2010. A new Argentinean nesting site showing neosauropod dinosaur reproduction in a Cretaceous hydrothermal environment. Nature Communications 1: 10.1038/Ncomms1031.

Grellet-Tinner, G., Fiorelli, L.E. and Salvador, R.B., 2012. Water vapor conductance of the Lower Cretaceous dinosaurian eggs from Sanagasta, La Rioja, Argentina: paleobiological and paleoecological implications for South American faveoloolithid and megaloolithid eggs. Palaios 27: 35-47.

Grellet-Tinner, G. and Makovicky, P., 2006. A possible egg of the dromaeosaur *Deinonychus antirrhopus*: phylogenetic and biological implications. Canadian Journal of Earth Sciences 43: 705-719.

Grigorescu, D., 2016. The 'Tuştea puzzle' revisited: Late Cretaceous (Maastrichtian) Megaloolithus eggs associated with *Telmatosaurus* hatchlings in the Haţeg Basin, Historical Biology 29: 627-640.

Grigorescu, D., Garcia, G., Csiki, Z., Codrea, V. and Bojar, A.V., 2010. Uppermost Cretaceous megaloolithid eggs from the Hateg

Basin, Romania, associated with hadrosaur hatchlings: search for explanation. Palaeogeography Palaeoclimatology Palaeoecology 293: 360-374.

Grigorescu, D., Weishampel, D.B., Norman, D., Seclamen, M., Rusu, M., Baltres, A. and Teodorescu, V., 1994. Late Maastrichtian dinosaur eggs from the Hateg Basin (Romania). In: K. Carpenter, K.F. Hirsch and J.R. Horner (Editors), Dinosaur Eggs and Babies. Cambridge University Press, Cambridge, UK, pp. 75-87.

Hall, P.M. and Johnson, D.R., 1987. Nesting biology of *Crocodylus novaeguineae* in Lake Murray District, Papua New Guinea. Herpetologica 43: 249-258.

Hechenleitner, E.M., Fiorelli, L.E., Grellet-Tinner, G., Leuzinger, L., Basilici, G., Taborda, J.R.A., De la Vega, S.R. and Bustamante, C.A., 2016. A new Upper Cretaceous titanosaur nesting site from La Rioja (NW Argentina), with implications for titanosaur nesting strategies. Palaeontology 59: 433-446.

Heij, C.J., Rompas, C.F.E. and Moeliker, C.W., 1997. The biology of the Moluccan Megapode *Eulipoa wallacei* (Aves, Galliformes, Megapodiidae) on Haruku and other Moluccan Islands; part 2: final report. Deinsea 3: 1-123.

Herron, J.C., Emmons, L.H. and Cadle, J.E., 1990. Observations on reproduction in the Black Caiman, *Melanosuchus niger*. Journal of Herpetology 24: 314-316.

Hirsch, K.F. and Quinn, B., 1990. Eggs and eggshell fragments from the Upper Cretaceous Two Medicine Formation of Montana. Journal of Vertebrate Paleontology 10: 491-511.

Hirsch, K.F., Stadtman, K.L., Miller, W.E. and Madsen, J.H., 1989. Upper Jurassic dinosaur egg from Utah. Science 243: 1711-1713.

Horner, J.R., 1982. Evidence of colonial nesting and 'site fidelity' among ornithischian dinosaurs. Nature 297: 675-676.

Horner, J.R., 1999. Egg clutches and embryos of two hadrosaurian dinosaurs. Journal of Vertebrate Paleontology 19: 607-611.

Horner, J.R., 2000. Dinosaur reproduction and parenting. Annual Review of Earth and Planetary Sciences 28: 19-45.

Horner, J.R. and Currie, P.J., 1994. Embryonic and neonatal morphology and ontogeny of a new species of *Hypacrosaurus* (Ornithischia, Lambeosauridae) from Montana and Alberta. In: K. Carpenter, K.F. Hirsch and J.R. Horner (Editors), Dinosaur

Eggs and Babies. Cambridge University Press, Cambridge, UK, pp. 312-336.

Horner, J.R. and Makela, R., 1979. Nest of juveniles provides evidence of family structure among dinosaurs. Nature 282: 296-298.

Huh, M., Kim, B.S., Woo, Y., Simon, D.J., Paik, I.S. and Kim, H.J., 2014. First record of a complete giant theropod egg clutch from Upper Cretaceous deposits, South Korea. Historical Biology 26: 218-228.

Huh, M. and Zelenitsky, D.K., 2002. Rich dinosaur nesting site from the Cretaceous of Bosung County, Chullanam-do Province, South Korea. Journal of Vertebrate Paleontology 22: 716-718.

Indrawan, M., Masala, Y. and Pesik, L., 1998. Observations on the breeding behaviour of Sula Scrubfowl *Megapodius bernsteinii* in the Banggai Islands, Sulawesi, Indonesia. Forktail 13: 13-16.

Jackson, F.D., 2007. Titanosaur reproductive biology: comparison of the Auca Mahuevo titanosaur nesting locality (Argentina), to the Pinyes *Megaloolithus* nesting locality (Spain). Ph.D. Thesis, Montana State University, Bozeman, 166 pp.

Jackson, F.D., Horner, J.R. and Varricchio, D.J., 2010. A study of a *Troodon* egg containing embryonic remains using epifluorescence microscopy and other techniques. Cretaceous Research 31: 255-262.

Jackson, F.D., Schaff, R.J., Varricchio, D.J. and Schmitt, J.G., 2015. A theropod nesting trace with eggs from the Upper Cretaceous (Campanian) Two Medicine Formation of Montana. Palaios 30: 362-372.

Jackson, F.D., Schmitt, J.G. and Oser, S.E., 2013. Influence of Vertisol development on sauropod egg taphonomy and distribution at the Auca Mahuevo locality, Patagonia, Argentina. Palaeogeography Palaeoclimatology Palaeoecology 386: 300-307.

Jackson, F.D. and Varricchio, D.J., 2010. Fossil eggs and eggshell from the lowermost Two Medicine Formation of western Montana, Sevenmile Hill locality. Journal of Vertebrate Paleontology 30: 1142-1156.

Jensen, J.A., 1966. Dinosaur eggs from the Upper Cretaceous North Horn Formation of Central Utah. Brigham Young University Geology Studies 13: 55-67.

Ji, Q., 2009. Study on dinosaur eggs in China: yesterday and today. Acta Geoscientia Sinica 30: 285-290.

Jiang, X.J., Liu, Y.Q., Ji, S.A., Zhang, X.L., Xu, L., Jia, S.H., Lü, J.C., Yuan, C.X. and Li, M., 2011. Dinosaur-bearing strata and K/T

boundary in the Luanchuan-Tantou Basin of western Henan Province, China. Science China-Earth Sciences 54: 1-7.

Jin, X.S., Azuma, Y., Jackson, F.D. and Varricchio, D.J., 2007. Giant dinosaur eggs from the Tiantai basin, Zhejiang Province, China. Canadian Journal of Earth Sciences 44: 81-88.

Jin, X.S., Jackson, F.D., Varricchio, D.J., Azuma, Y. and He, T., 2010. The first *Dictyoolithus* egg clutches from the Lishui Basin, Zhejiang Province, China. Journal of Vertebrate Paleontology 30: 188-195.

Joanen, T., 1969. Nesting ecology of alligators in Louisiana. Proceedings of the Annual Conference, Southeastern Association of Game and Fish Commissioneres 24: 141-151.

Jones, D.N., 1988. Construction and maintenance of the incubation mounds of the Australian Brush-Turkey *Alectura lathami*. Emu 88: 210-218.

Jones, D.N., Dekker, R.W.R.J. and Roselaar, C.S., 1995. The Megapodes. Oxford University Press, Oxford, UK, 262 pp.

Kellers, H.C., 1931. Biological collecting on "tin-can island". In: Explorations and Field-Work of the Smithsonian Institution in 1931. Smithsonian Institution, Washington, pp. 67-76.

Khosla, A. and Sahni, A., 1995. Parataxonomic classification of Late Cretaceous dinosaur eggshells from India. Journal of The Palaeontological Society of India 40: 87-102.

Kim, J.Y., Yang, S.Y., Choi, H., Seo, S.J. and Kim, K.S., 2011. Dinosaur eggs from the Cretaceous Goseong Formation of Tongyeong City, southern coast of Korea. Journal of Paleontological Society of Korea 27: 13-26.

Kim, S.B., Kim, Y.G., Jo, H.R., Jeong, K.S. and Chough, S.K., 2009. Depositional facies, architecture and environments of the Sihwa Formation (Lower Cretaceous), mid-west Korea with special reference to dinosaur eggs. Cretaceous Research 30: 100-126.

Kitching, J.W., 1979. Preliminary report on a clutch of six dinosaurian eggs from the Upper Triassic Elliot Formation, Northern Orange Free State. Palaeontologia Africana 22: 41-45.

Kofron, C.P., 1989. Nesting ecology of the Nile crocodile (*Crocodylus niloticuls*). African Journal of Ecology 27: 335-341.

Krumenacker, L.J., Simon, D.J., Scofield, G. and Varricchio, D.J., 2016. Theropod dinosaurs from the Albian-Cenomanian Wayan

Formation of eastern Idaho. Historical Biology: 10.1080/08912963.2015.1137913.

Kundrat, M., Cruickshank, A.R.I., Manning, T.W. and Nudds, J., 2008. Embryos of therizinosauroid theropods from the Upper Cretaceous of China: diagnosis and analysis of ossification patterns. Acta Zoologica 89: 231-251.

Kurzanov, S.M. and Mikhailov, K.E., 1989. Dinosaur eggshells from the Lower Cretaceous of Mongolia. In: D.D. Gillette and M.G. Lockley (Editors), Dinosaur Tracks and Traces. Cambridge University Press, New York, pp. 109-113.

Kushlan, J.A. and Mazzotti, F.J., 1989. Population biology of the American Crocodile. Journal of Herpetology 23: 7-21.

Le Souef, D., 1899. On the habits of the mound-building birds of Australia. Ibis 5: 9-19.

Lee, Y.-N., 2003. Dinosaur bones and eggs in South Korea. Memoir of the Fukui Prefectural Dinosaur Museum 2: 113-121.

Lewis, F., 1940. Notes on the breeding habits of the Mallee-Fowl. Emu 40: 97-110.

Li, Y., Yin, Z. and Liu, Y., 1995. The discovery of a new genus of dinosaur egg from Xixia, Henan, China. Journal of Wuhan Institute of Chemical Technology 17: 38-40.

Liang, X., Wen, S., Yang, D., Zhou, S. and Wu, S., 2009. Dinosaur eggs and dinosaur egg-bearing deposits (Upper Cretaceous) of Henan Province, China: occurrences, palaeoenvironments, taphonomy and preservation. Progress in Natural Science 19: 1587-1601.

Lincoln, G.A., 1974. Predation of incubator birds (*Megapodius freycinet*) by Komodo dragons (*Varanus komodoensis*). Journal of Zoology 174: 419-428.

Liston, J.J. and Chapman, S.D., 2014. Alfred Nicholson Leeds and the first fossil egg attributed to a 'saurian'. Historical Biology 26: 229-235.

Liu, J.Y., Wang, Q., Zhao, Z., Wang, X.L., Gao, C.L. and Shen, C.Z., 2013. A parataxonomic revision of spheroolithid eggs from the Upper Cretaceous Quantou Formation in Changtu, Liaoning. Vertebrata Palasiatica 51: 278-288.

Lopez-Luna, M.A., Hidalgo-Mihart, M.G., Aguirre-Leon, G., Gonzalez-Ramon, M.D. and Rangel-Mendoza, J.A., 2015. Effect of nesting environment on incubation temperature and hatching success of Morelet's crocodile (*Crocodylus moreletii*) in an urban

lake of Southeastern Mexico. Journal of Thermal Biology 49/50: 66-73.

Lopez-Martinez, N., Moratalla, J.J. and Sanz, J.L., 2000. Dinosaurs nesting on tidal flats. Palaeogeography Palaeoclimatology Palaeoecology 160: 153-163.

Lopez-Martinez, N. and Vicens, E., 2012. A new peculiar dinosaur egg, *Sankofa pyrenaica* oogen. nov. oosp. nov. from the Upper Cretaceous coastal deposits of the Aren Formation, south-central Pyrenees, Lleida, Catalonia, Spain. Palaeontology 55: 325-339.

Lü, J., Azuma, Y., Huang, D., Noda, Y. and Qiu, L., 2006. New troodontid dinosaur eggs from the Heyuan Basin of Guangdong Province, Southern China. In: J. Lü, Y. Kobayashi, D. Huang and Y.N. Lee (Editors), Papers from the 2005 Heyuan International Dinosaur Symposium. Geological Publishing House, Beijing, China, pp. 11-18.

Lutz, P.L. and Dunbar-Cooper, A., 1984. The nest environment of the American Crocodile (*Crocodylus acutus*). Copeia 1984: 153-161.

MacKinnon, J., 1978. Sulawesi Megapodes. Journal of the World Pheasant Association 3: 96-103.

Magnusson, W.E., Lima, A.P. and Sampaio, R.M., 1985. Sources of heat for nests of *Paleosuchus trigonatus* and a review of crocodilian nest temperatures. Journal of Herpetology 19: 199-207.

Makovicky, P.J. and Grellet-Tinner, G., 2001. Association between theropod eggshell and a specimen of *Deinonychus antirrhopus*. In: A.M. Bravo and T. Reyes (Editors), First International Symposium on Dinosaur Eggs and Babies Extended Abstracts, Catalonia, Spain, pp. 123-128.

Manning, T.W., Joysey, K.A. and Cruickshank, A.R.I., 1997. Observations of microstructures within dinosaur eggs from Henan Province, Peoples' Republic of China. In: D.L. Wolberg, E. Stump and R.D. Rosenberg (Editors), Dinofest International: Proceedings of a Symposium Held at Arizona State University. Academy of Natural Sciences, Philadelphia, pp. 287-290.

Mateus, I., Mateus, H., Antunes, M.T., Mateus, O., Taquet, P., Ribeiro, V. and Manuppella, G., 1997. Clutches, eggs and embryos of therapod dinosaur in the upper Jurassic of Lourinha, Portugal. Comptes Rendus de l'Académie des Sciences-Series II: A-Earth and Planetary Sciences 325: 71-78.

Mauro, I., 2005. Field discovery, mound characteristics, bare parts, vocalisations and behaviour of Bruijn's Brush-turkey (*Aepypodius bruijnii*). Emu 105: 273-281.

Maxwell, W.D. and Horner, J.R., 1994. Neonate dinosaurian remains and dinosaurian eggshell from the Cloverly Formation, Montana. Journal of Vertebrate Paleontology 14: 143-146.

Mazzotti, F.J., 1989. Factors affecting the nesting success of the American Crocodile, *Crocodylus acutus*, in Florida Bay. Bulletin of Marine Science 44: 220-228.

McIlhenny, E.A., 1934. Notes on incubation and growth of alligators. Copeia 2: 80-88.

Medem, F., 1958. The crocodilian genus *Paleosuchus*. Fieldiana Zoology 39: 227-247.

Medem, F., 1971. The reproduction of the Dwarf Caiman *Paleosuchus palpebrosus*, Crocodiles: Proceedings of the 1st Working Meeting of Crocodile Specialists. IUCN, New York, pp. 159-165.

Medem, F., 1981. Los Crocodylia de sur America Volumen 1. Los Crocodylia de Colombia. Universidad Nacional de Colombia, Bogota, Colombia, 354 pp.

Metzen, W.D., 1977. Nesting ecology of alligators on the Okefenokee National Wildlife Refuge. Proceedings of the Southeastern Association of Fish and Wildlife Agencies 31: 29-32.

Mikhailov, K., 1994a. Eggs of sauropod and ornithopod dinosaurs from the Cretaceous deposits of Mongolia. Paleontological Journal 28: 141-159.

Mikhailov, K., 1994b. Theropod and protoceratopsian dinosaur eggs from the Cretaceous of Mongolia and Kazakhstan. Paleontological Journal 28: 101-120.

Modha, M.L., 1967. The ecology of the Nile Crocodile (*Crocodylus niloticus* Laurenti) on Central Island, Lake Rudolf. African Journal of Ecology 5: 74-95.

Mohabey, D.M., 1996. A new oospecies, *Megaloolithus matleyi*, from the Lameta Formation (Upper Cretaceous) of Chandrapur district, Maharashtra, India, and general remarks on the palaeoenvironment and nesting behaviour of dinosaurs. Cretaceous

Research 17: 183-196.

Mohabey, D.M., 1998. Systematics of Indian Upper Cretaceous dinosaur and chelonian eggshells. Journal of Vertebrate Paleontology 18: 348-362.

Mohabey, D.M., 2000. Indian dinosaur eggs: a review. Journal of the Geological Society of India 58: 479-508.

Mohabey, D.M., 2001. Indian Upper Cretaceous (Maastrichtian) dinosaur eggs: their parataxonomy and implications in understanding the nesting behaviour. In: A.M. Bravo and T. Reyes (Editors), First International Symposium on Dinosaur Eggs and Babies Extended Abstracts, Catalonia, Spain, pp. 139-154.

Mohabey, D.M. and Mathur, U.B., 1989. Upper Cretaceous dinosaur eggs from new localities of Gujarat, India. Journal of the Geological Society of India 33: 32-37.

Mones, A., 1980. Nuevos elementos de la paleoherpetofauna del uruguay (Crocodilia y Dinosauria). Actas del Segundo Congreso Argentino de Paleontología y Bioestratigrafía y Primer Congreso Latinoamericano de Paleontología: Buenos Aires, Argentina, 2-6 de abril, 1978, 1: 265-278.

Montini, J.P., Piña, C.I., Larriera, A., Siroski, P. and Verdade, L.M., 2006. The relationship between nesting habitat and hatching success in *Caiman latirostris* (Crocodylia, Alligatoridae). Phyllomedusa: Journal of Herpetology 5: 91-96.

Moreno-Azanza, M., Canudo, J.I. and Gasca, J.M., 2014a. Spheroolithid eggshells in the Lower Cretaceous of Europe. Implications for eggshell evolution in ornithischian dinosaurs. Cretaceous Research 51: 75-87.

Moreno-Azanza, M., Canudo, J.I. and Gasca, J.M., 2014b. Unusual theropod eggshells from the Early Cretaceous Blesa Formation of the Iberian Range, Spain. Acta Palaeontologica Polonica 59: 843-854.

Nair, T. and Katdare, S., 2013. Dry-season assessment of gharials (*Gavialis gangeticus*) in the Betwa, Ken and Son Rivers, India. In: World crocodile conference. Proceedings of the 22nd Working Meeting of the IUCN-SSC Crocodile Specialist Group. IUCN, Gland, Switzerland, pp. 53-65.

Neil, W.T., 1946. Notes on *Crocodylus novaeguineae*. Copeia 1946: 17-20.

Norell, M., Clark, J.M. and Chiappe, L.M., 2001. An embryonic oviraptorid (Dinosauria: Theropoda) from the Upper Cretaceous of Mongolia. American Museum Novitates 3315: 1-17.

Norell, M.A., Clark, J.M., Demberelyin, D., Rhinchen, B., Chiappe, L.M., Davidson, A.R., Mckenna, M.C., Altangerel, P. and Novacek, M.J., 1994. A theropod dinosaur embryo and the affinities of the Flaming Cliffs dinosaur eggs. Science 266: 779-782.

Ogden, J.C., 1978. Status and nesting biology of American Crocodile, *Crocodylus acutus*, (Reptilia, Crocodilidae) in Florida. Journal of Herpetology 12: 183-196.

Ouboter, P.E. and Nanhoe, L.M.R., 1987. Notes on nesting and parental care in *Caiman crocodilus crocodilus* in northern Suriname and an analysis of crocodilian nesting habitats. Amphibia-Reptilia 8: 331-347.

Paik, I.S., Huh, M. and Kim, H.J., 2004. Dinosaur egg-bearing deposits (Upper Cretaceous) of Boseong, Korea: occurrence, palaeoenvironments, taphonomy, and preservation. Palaeogeography Palaeoclimatology Palaeoecology 205: 155-168.

Paik, I.S., Kim, H.J. and Huh, M., 2012. Dinosaur egg deposits in the Cretaceous Gyeongsang Supergroup, Korea: diversity and paleobiological implications. Journal of Asian Earth Sciences 56: 135-146.

Peitz, C., 2001. Megaloolithid dinosaur eggs from the Maastrichtian of Catalunya (NE-Spain) - parataxonomic implications and stratigraphic utility. In: A.M. Bravo and T. Reyes (Editors), First International Symposium on Dinosaur Eggs and Babies Extended Abstracts, Catalonia, Spain, pp. 155-160.

Platt, S.G., Rainwater, T.R., Thorbjarnarson, J.B. and McMurry, S.T., 2008. Reproductive dynamics of a tropical freshwater crocodilian: Morelet's crocodile in northern Belize. Journal of Zoology 275: 177-189.

Platt, S.G. and Thorbjarnarson, J.B., 2000. Nesting ecology of the American crocodile in the coastal zone of Belize. Copeia: 869-873.

Pooley, A.C., 1969. Preliminary studies on the breeding of the Nile Crocodile *Crocodylus niloticus*, in Zululand. Lammergeyer 10: 22-44.

Qian, M., Jiang, Y., Chen, R., Jiang, Y., Zhang, Y.L. and Xin, G., 2008. Late Cretaceous troodontid eggs from Tiantai, Zhejiang. Acta Palaeontologica Sinica: 248-255.

Rand, A.L. and Gilliard, E.T., 1967. Handbook of New Guinea Birds. Weidenfeld and Nicolson, London, UK, 612 pp.

Rao, R.J., 1988. Nesting Ecology of the Gharial in National Chambal Sanctuary. Wildlife Institute of India, Dehradun, India, 105 pp.

Rao, R.J. and Gaurjwar, R.K., 2013. Crocodile human conflict in National Chambal Sanctuary, India. In: World Crocodile Conference: Proceedings of the 22nd Working Meeting of the IUCN-SSC Crocodile Specialist Group. IUCN, Gland, Switzerland, pp. 105-109.

Reisz, R.R., Evans, D.C., Roberts, E.M., Sues, H.D. and Yates, A.M., 2012. Oldest known dinosaurian nesting site and reproductive biology of the Early Jurassic sauropodomorph *Massospondylus*. Proceedings of the National Academy of Sciences of the United States of America 109: 2428-2433.

Reisz, R.R., Huang, T.D., Roberts, E.M., Peng, S.R., Sullivan, C., Stein, K., LeBlanc, A.R.H., Shieh, D., Chang, R.S., Chiang, C.C., Yang, C.W. and Zhong, S.M., 2013. Embryology of Early Jurassic dinosaur from China with evidence of preserved organic remains. Nature 496: 210-214.

Ribeiro, C.M.M., 2002. Ovo e fragmentos de cascas de ovos de dinossauros, provenientes da regiao de Peiropolis, Uberaba, Minas Gerais. Arquivos do Museu Nacional 60: 223-228.

Ribeiro, V., Mateus, O., Holwerda, F., Araujo, R. and Castanhinha, R., 2014. Two new theropod egg sites from the Late Jurassic Lourinha Formation, Portugal. Historical Biology 26: 206-217.

Riley, J. and Huchzermeyer, F.W., 1999. African dwarf crocodiles in the Likouala swamp forests of the congo basin: habitat, density, and nesting. Copeia: 313-320.

Ripley, S.D., 1960. Distribution and niche differentiation in species of megapodes in the Moluccas and western Papuan Area. Proceedings of the XIIth International Ornithological Congress, Helsinki, 1958: 631-640.

Rivas, J.A., Aktay, S.A. and Owens, R.Y., 2001. *Paleosuchus trigonatus* (Schneider's Smooth-fronted Caiman) nesting. Herpetological Review 32: 251.

Roper, D.S., 1983. Egg incubation and laying behavior of the incubator bird *Megapodius freycinet* on Savo. Ibis 125: 384-389.

Sabath, K., 1991. Upper Cretaceous amniotic eggs from the Gobi Desert. Palaeontologica 36: 151-192.

Salgado, L., Coria, R.A., Ribeiro, C.M.M., Garrido, A., Rogers, R., Simon, M.E., Arcucci, A.B., Rogers, K.C., Carabajal, A.P., Apesteguia, S., Fernandez, M., Garcia, R.A. and Talevi, M., 2007. Upper Cretaceous dinosaur nesting sites of Rio Negro (Salitral Ojo de Agua and Salinas de Trapalco-Salitral de Santa Rosa), northern Patagonia, Argentina. Cretaceous Research 28: 392-404.

Salgado, L., Ribeiro, C.M., Garcia, R.A. and Fernandez, M.S., 2009. Late Cretaceous megaloolithid eggs from Salitral de Santa Rosa (Rio Negro, Patagonia, Argentina): inferences on the titanosaurian reproductive biology. Ameghiniana 46: 605-620.

Sander, P.M., Peitz, C., Gallemi, J. and Cousin, R., 1998. Dinosaurs nesting on a red beach? Comptes Rendus de l'Académie des Sciences-Series II: A-Earth and Planetary Sciences 327: 67-74.

Sankaran, R., 1995. The distribution, status and conservation of the Nicobar Megapode *Megapodius nicobariensis*. Biological Conservation 72: 17-25.

Sanz, J.L., Moratalla, J.J., Diazmolina, M., Lopezmartinez, N., Kalin, O. and Vianeyliaud, M., 1995. Dinosaur nests at the sea shore. Nature 376: 731-732.

Sato, T., Cheng, Y.N., Wu, X.C., Zelenitsky, D.K. and Hsiao, Y.F., 2005. A pair of shelled eggs inside a female dinosaur. Science 308: 375-375.

Seijas, A.E., 1998. The Orinoco Crocodile (*Crocodylus intemedius*) in the Cojedes River System, Venezuela: population status and ecological characteristics. Ph.D. Thesis, University of Florida, Gainesville, 191 pp.

Sellés, A.G., Bravo, A.M., Delclos, X., Colombo, F., Marti, X., Ortega-Blanco, J., Parellada, C. and Galobart, A., 2013. Dinosaur eggs in the Upper Cretaceous of the Coll de Nargo area, Lleida Province, south-central Pyrenees, Spain: oodiversity, biostratigraphy and their implications. Cretaceous Research 40: 10-20.

Sellés, A.G., Via, B. and Galobart, A., 2014. *Spheroolithus europaeus*, oosp. nov. (late Maastrichtian, Catalonia), the youngest oological record of hadrosauroids in Eurasia. Journal of Vertebrate Paleontology 34: 725-729.

Seymour, R.S. and Bradford, D.F., 1992. Temperature regulation in the incubation mounds of the Australian Brush-Turkey. Condor 94: 134-150.

Sinclair, J.R., 2001. Temperature regulation in mounds of three sympatric species of megapode (Aves: Megapodiidae) in Papua New Guinea: testing the 'Seymour Model'. Australian Journal of Zoology 49: 675-694.

Sivakumar, K. and Sankaran, R., 2003. The incubation mound and hatching success of the Nicobar Megapode *Megapodius nicobariensis* Blyth. Journal of the Bombay Natural History Society 100: 375-387.

Smith, T., Codrea, V.A., Sasaran, E., Van Itterbeeck, J., Bultynck, P., Csiki, Z., Dica, P., Farcas, C., Folie, A., Garcia, G. and Godefroit, P., 2002. A new exceptional vertebrate site from the Late Cretaceous of the Hateg Basin (Romania). Studia Universitatis Babes-Bolyai, Geologia, Special Issue 1: 321-330.

Sochava, A.V., 1969. Dinosaur eggs from the Upper Cretaceous of the Gobi Desert. Paleontological Journal 4: 517-527.

Somaweera, R. and Shine, R., 2013. Nest-site selection by crocodiles at a rocky site in the Australian tropics: making the best of a bad lot. Austral Ecology 38: 313-325.

Soto, M., Perea, D. and Cambiaso, A., 2012. First sauropod (Dinosauria: Saurischia) remains from the Guichon Formation, Late Cretaceous of Uruguay. Journal of South American Earth Sciences 33: 68-79.

Srivastava, A.K. and Mankar, R.S., 2015. *Megaloolithus* dinosaur nest from the Lameta Formation of Salbardi area, districts Amravati, Maharashtra and Betul, Madhya Pradesh. Journal of the Geological Society of India 85: 457-462.

Srivastava, S., Mohabey, D.M., Sahni, A. and Pant, S.C., 1986. Upper Cretaceous dinosaur egg clutches from Kheda District (Gujarat, India). Palaeontographica Abteilung A: Palaozoologie-Stratigraphie 193: 219-233.

Staniewicz, A., 2011. Diet and demography of *Tomistoma schlegelii* in Mesangat Lake, East Kalimantan, Indonesia: June–September 2011. University of Bristol, School of Biological Sciences. Unpublished Report for EAZA IUCN/SSC Southeast Asia Campaign: See http://www.dght-zgap-artenschutz.de/media/projekte/mesangat/2011%20Mesangat%20Tomistoma%20report.pdf.

Staton, M.A. and Dixon, J.R., 1977. Breeding biology of the Spectacled Caiman, *Caiman crocodilus crocodilus*, in the Venezuelan Llanos. In: Wildlife Research Report 5. United States Department of the Interior, Fish and Wildlife Service, Washington, pp. 1-21.

Stinson, D.W. and Glass, P.O., 1992. The Micronesian megapode *Megapodius laperouse*: conservation and research needs. Zoologische Verhandelingen 278: 53-55.

Stresemann, E., 1914. Beiträge zur Kenntnis der Avifauna von Buru. Notitates Zoologicae 21: 358-400.

Stuebing, R. and Johan, Z., 1986. The megapodes of Pulau Tiga. The Sabah Museum and State Archives Journal 1: 16-49.

Suzuki, S. and Watabe, M., 2000a. Report on the Japan - Mongolia Joint Paleontological Expedition to the Gobi desert, 1995. Hayashibara Museum of Natural Sciences Research Bulletin 1: 45-57.

Suzuki, S. and Watabe, M., 2000b. Report on the Japan - Mongolia Joint Paleontological Expedition to the Gobi desert, 1998. Hayashibara Museum of Natural Sciences Research Bulletin 1: 83-98.

Swanepoel, D.G.J., Ferguson, N.S. and Perrin, M.R., 2000. Nesting ecology of Nile crocodiles (*Crocodylus niloticus*) in the Olifants River, Kruger National Park. Koedoe 43: 35-46.

Tanaka, K., Lü, J., Liu, Y., Huang, Z., Kobayashi, Y., Huang, D. and Zelenitsky, D.K., 2012. Statistical approach for classification of dinosaur eggs from the Heyuan Basin at the northeast of Guangdong Provice. Acta Geologica Sinica 86: 294-303.

Tanaka, K., Lü, J.C., Kobayashi, Y., Zelenitsky, D.K., Xu, L., Jia, S.G., Qin, S.A. and Tang, M.N., 2011. Description and phylogenetic position of dinosaur eggshells from the Luanchuan area of western Henan Province, China. Acta Geologica Sinica (English Edition) 85: 66-74.

Tanaka, K., Zelenitsky, D.K., Saegusa, H., Ikeda, T., DeBuhr, C.L. and Therrien, F., 2016. Dinosaur eggshell assemblage from Japan reveals unknown diversity of small theropods. Cretaceous Research 57: 350-363.

Thorbjarnarson, J., Wang, X.M. and He, L.J., 2001. Reproductive ecology of the Chinese alligator (*Alligator sinensis*) and implications for conservation. Journal of Herpetology 35: 553-558.

Thorbjarnarson, J.B. and Hernandez, G., 1993. Reproductive ecology of the Orinoco Crocodile (*Crocodylus intermedius*) in Venezuela. I. Nesting ecology and egg and clutch relationships. Journal of Herpetology 27: 363-370.

van Straelen, V., 1925. The microstructure of the dinosaurian egg-shells from the Cretaceous beds of Mongolia. American Museum Novitates 173: 1-4.

van Weerd, M., 2010. Philippine Crocodile *Crocodylus mindorensis*. In: S.C. Manolis and C. Stevenson (Editors), Crocodiles: Status, Survey and Conservation Action Plan. Crocodile Specialist Group, Darwin, Australia, pp. 71-78.

van Weerd, M., van der Ploeg, J., Rodriguez, D., Guerrero, J., Tarun, B., Telan, S. and de Jonge, J., 2006. Philippine crocodile conservation in Northeast Luzon: an update of population status and new insights into *Crocodylus mindorensis* ecology. In: Crocodiles: Proceedings of the 18th Working Meeting of the Crocodile Specialist Group. IUCN - the World Conservation Union, Gland, Switzerland and Cambridge, UK, pp. 306-321.

Varricchio, D.J., Horner, J.R. and Jackson, F.D., 2002. Embryos and eggs for the Cretaceous theropod dinosaur *Troodon formosus*. Journal of Vertebrate Paleontology 22: 564-576.

Varricchio, D.J., Jackson, F., Borkowski, J.J. and Horner, J.R., 1997. Nest and egg clutches of the dinosaur *Troodon formosus* and the evolution of avian reproductive traits. Nature 385: 247-250.

Varricchio, D.J., Jackson, F. and Trueman, C.N., 1999. A nesting trace with eggs for the Cretaceous theropod dinosaur *Troodon formosus*. Journal of Vertebrate Paleontology 19: 91-100.

Varricchio, D.J., Jin, X.-S. and Jackson, F.D., 2015. Lay, brood, repeat: nest reuse and site fidelity in ecologic time for two Cretaceous troodontid dinosaurs. Journal of Vertebrate Paleontology 35: e932797.

Venczel, M., Vasile, S. and Csiki-Sava, Z., 2015. A Late Cretaceous madtsoiid snake from Romania associated with a megaloolithid egg nest - paleoecological inferences. Cretaceous Research 55: 152-163.

Vianey-Liaud, M., Hirsch, K.E., Sahni, A. and Sige, B., 1997. Late Cretaceous Peruvian eggshells and their relationships with Laurasian and Eastern Gondowanian material. Geobios 30: 75-90.

Vianey-Liaud, M., Khosla, A. and Garcia, G., 2003. Relationships between European and Indian dinosaur eggs and eggshells of the oofamily Megaloolithidae. Journal of Vertebrate Paleontology 23: 575-585.

Vianey-Liaud, M. and Lopez-Martinez, N., 1997. Late Cretaceous dinosaur eggshells from the Tremp Basin, southern Pyrenees, Lleida, Spain. Journal of Paleontology 71: 1157-1171.

Vianey-Liaud, M., Mallan, P., Buscail, O. and Montgelard, C., 1994. Review of French dinosaur eggshells; morphology, structure, mineral, and organic composition. In: K. Carpenter, K.F. Hirsch and J.R. Horner (Editors), Dinosaur Eggs and Babies. Cambridge University Press, Cambridge, UK, pp. 151-183.

Vila, B., Galobart, A., Oms, O., Poza, B. and Bravo, A.M., 2010b. Assessing the nesting strategies of Late Cretaceous titanosaurs: 3-D clutch geometry from a new megaloolithid eggsite. Lethaia 43: 197-208.

Vila, B., Jackson, F.D., Fortuny, J., Selles, A.G. and Galobart, A., 2010a. 3-D modelling of megaloolithid clutches: insights about nest construction and dinosaur behaviour. PLoS One 5: 10.1371/journal.pone.0010362.

Vila, B., Selles, A.G. and Beetschen, J.-C., 2017. The controversial Les Labadous eggshells: a new and peculiar dromaeosaurid (Dinosauria: Theropoda) ootype from the Upper Cretaceous of Europe. Cretaceous Research 72: 117-123.

VillamarIn-Jurado, F. and Suarez, E., 2007. Nesting of the Black Caiman (*Melanosuchus niger*) in Northeastern Ecuador. Journal of Herpetology 41: 164-167.

Waitkuwait, W.E., 1985. Investigations of the breeding biology of the West-african slender-snouted crocodile *Crocodylus cataphractus* Cuvier, 1824. Amphibia-Reptilia 6: 387-399.

Wang, D. and Zhou, S., 1995. The discovery of new typical dinosaur egg fossils in Xixia Basin. Henan Geology 13: 262-267.

Wang, Q., Wang, X.L., Zhao, Z.K. and Jiang, Y.G., 2010. A new oogenus of Elongatoolithidae from the Upper Cretaceous Chichengshan Formation of Tiantai Basin, Zhejiang Province. Vertebrata Palasiatica 48: 111-118.

Wang, Q., Zan, S., Jin, L.Y. and Chen, J., 2006. A new oospecies, *Dictyoolithus gongzhulingensis*, from the Early Cretaceous Quantou Formation in the central Jilin Province. Journal of Jilin University (Earth Science Edition) 36: 153-157.

Wang, Q., Zhao, Z., Wang, X., Zhang, S.R. and Jiang, Y., 2013. New forms of dictyoolithids from the Tiantai Basin, Zhejiang Province of China and a parataxonomic revision of the dictyoolithids. Vertebrata PalAsiatica 51: 43-54.

Wang, Q., Zhao, Z., Wang, X.L. and Jiang, Y., 2011. New ootypes of dinosaur eggs from the Late Cretaceous in Tiantai Basin, Zhejiang Province, China. Vertebrata Palasiatica 49: 446-449.

Wang, S., Zhang, S., Sullivan, C. and Xu, X., 2016. Elongatoolithid eggs containing oviraptorid (Theropoda, Oviraptorosauria) embryos from the Upper Cretaceous of Southern China. BMC Evolutionary Biology 16: 1-21.

Watabe, M., 2004. New dinosaur ovifauna from the Upper Cretaceous vertebrate fossil locality, Abdrant Nuru, Central part of the Gobi desert, Mongolia. Hayashibara Museum of Natural Sciences Research Bulletin 2: 15-27.

Watabe, M., Tsogtbaatar, K., Suzuki, S. and Saneyoshi, M., 2010. Geology of dinosaur-fossil-bearing localities (Jurassic and Cretaceous: Mesozoic) in the Gobi Desert: results of the HMNS-MPC Joint Paleontological Expedition. Hayashibara Museum of Natural Sciences Research Bulletin 3: 41-118.

Webb, G.J.W., Buckworth, R. and Manolis, S.C., 1983a. *Crocodylus johnstoni* in the Mckinlay River N.T. VI. Nesting biology. Australian Wildlife Research 10: 607-637.

Webb, G.J.W., Messel, H. and Magnusson, W., 1977. Nesting of *Crocodylus porosus* in Arnhem Land, Northern Australia. Copeia: 238-249.

Webb, G.J.W., Sack, G.C., Buckworth, R. and Manolis, S.C., 1983b. An examination of *Crocodylus porosus* nests in two northern Australian fresh water swamps, with an analysis of embryo mortality. Australian Wildlife Research 10: 571-605.

Weir, D.G., 1973. Status and habits of *Megapodius pritchardii*. Wilson Bulletin 85: 79-82.

Welsh, E. and Sankey, J.T., 2005. First dinosaur eggshells from Texas, USA: Aguja Formation (Late Campanian), Big Bend National Park. In: J.T. Sankey and S. Baszio (Editors), Vertebrate Microfossil Assembrages: Their Role in Paleoecology and Paleobiogeography. Indiana University Press, Bloomington and Indianapolis, pp. 166-177.

Whitaker, R. and Whitaker, Z., 1976. Collection and hatching of Marsh Crocodile (*C. palustris*) eggs. Journal of Bombay Natural

History Society 73: 403-407.

Wiles, G.J. and Conry, P.J., 2001. Characteristics of nest mounds of Micronesian Megapodes in Palau. Journal of Field Ornithology 72: 267-275.

Williams, D.L.G., Seymour, R.S. and Kerourio, P., 1984. Structure of fossil dinosaur eggshell from the Aix Basin, France. Palaeogeography Palaeoclimatology Palaeoecology 45: 23-37.

Wilson, J.A., Mohabey, D.M., Peters, S.E. and Head, J.J., 2010. Predation upon hatchling dinosaurs by a new snake from the Late Cretaceous of India. PLoS Biology 8: 10.1371/journal.pbio.1000322

Wu, J. and Peng, A., 2003. Identification, paleogeography, and paleoclimate analyses of fossil dinosaur eggs from Baiyuan-Zhenyuan, Pingxiang, Jiangxi Province. Relics From South 3: 42-44.

Xie, J.-F., Zhang, S.-K., Jin, X.-S., Li, D.-Q. and Zhou, L.-Q., 2016. A new type of dinosaur eggs from Early Cretaceous of Gansu Province, China. Vertebrata PalAsiatica 54: 1-10.

Xu, L., 2007. The characters and protection of dinosaurs from Henan Province. Ph.D. Thesis, China University of Geosciences, Beijing, China, 120 pp.

Young, C.C., 1965. Fossil eggs from Nanhsiung, Kwangtung and Kanchou, Kiangsi. Vertebrata Palasiatica 9: 141-170.

Yun, C.-S. and Yang, S.-Y., 1997. Dinosaur eggshells from the Hasandong Formation, Gyeongsang Supergroup, Korea. Journal of the Paleontological Society of Korea 13: 21-36.

Zelenitsky, D.K., 2004. Description and phylogenetic implications of extant and fossil oologic remains. Ph.D. Thesis, University of Calgary, Calgary, Canada, 364 pp.

Zelenitsky, D.K., Carpenter, K. and Currie, P.J., 2000. First record of elongatoolithid theropod eggshell from North America: the Asian oogenus *Macroelongatoolithus* from the Lower Cretaceous of Utah. Journal of Vertebrate Paleontology 20: 130-138.

Zelenitsky, D.K. and Hills, L.V., 1996. An egg clutch of *Prismatoolithus levis* oosp. nov. from the Oldman Formation (Upper Cretaceous), Devil's Coulee, southern Alberta. Canadian Journal of Earth Sciences 33: 1127-1131.

Zelenitsky, D.K. and Hills, L.V., 1997. Normal and pathological eggshells of *Spheroolithus albertensis*, oosp. nov., from the Oldman Formation (Judith River Group, late Campanian), southern Alberta. Journal of Vertebrate Paleontology 17: 167-171.

Zelenitsky, D.K., Hills, L.V. and Currie, P.J., 1996. Parataxonomic classification of ornithoid eggshell fragments from the Oldman Formation (Judith River Group; Upper Cretaceous), southern Alberta. Canadian Journal of Earth Sciences 33: 1655-1667.

Zelenitsky, D.K. and Modesto, S.P., 2002. Re-evaluation of the eggshell structure of eggs containing dinosaur embryos from the Lower Jurassic of South Africa. South African Journal of Science 98: 407-408.

Zelenitsky, D.K. and Sloboda, W., 2005. Eggshells. In: P.J. Currie and E.B. Koppelhus (Editors), Dinosaur Provincial Park: a Spectacular Ancient Ecosystem Revealed. Indiana University Press, Bloomington, pp. 398-404.

Zelenitsky, D.K. and Therrien, F., 2008a. Phylogenetic analysis of reproductive traits of maniraptoran theropods and its implications for egg parataxonomy. Palaeontology 51: 807-816.

Zelenitsky, D.K. and Therrien, F., 2008b. Unique maniraptoran egg clutch from the Upper Cretaceous Two Medicine Formation of Montana reveals theropod nesting behaviour. Palaeontology 51: 1253-1259.

Zelenitsky, D.K., Therrien, F., Tanaka, K., Currie, P.J. and DeBuhr, C.L. 2017b. Latest Cretaceous eggshell assemblage from the Willow Creek Formation (upper Maastrichtian - lower Paleocene) of Alberta, Canada, reveals higher dinosaur diversity than represented by skeletal remains. Canadian Journal of Earth Science 54: 134-140.

Zelenitsky, D.K., Therrien, F., Tanaka, K., Kobayashi, Y. and DeBuhr, C.L. 2017a. Dinosaur eggshells from the Santonian Milk River Formation of Alberta, Canada. Cretaceous Research 74: 181-187.

Zeng, D. and Zhang, J., 1979. On the dinosaurian eggs from the western Dongting Basin, Hunan. Vertebrata Palasiatica 17: 131-136.

Zhang, S.-K., 2010. A parataxonomic revision of the Cretaceous faveoloolithid eggs of China. Vertebrata PalAsiatica 48: 203-219.

Zhang, W. and Huang, P., 1999. The strata with fossil dinosaur eggs and their distribution in Guangdong. Journal of Stratigraphy 23: 89-95.

Zhao, H. and Zhao, Z., 1998. Dinosaur eggs from Xichuan Basin, Henan Province. Vertebrata Palasiatica 36: 282-296.

Zhao, H. and Zhao, Z., 1999. A new form of elongatoolithid dinosaur eggs from the Lower Cretaceous Shahai Formation of Heishan, Liaoning Province. Vertebrata Palasiatica 37: 278-284.

Zhao, Z., 1979. Discovery of the dinosaurian eggs and footprint from Neixiang County, Henan Province. Vertebrata PalAsiatica 17: 304-309.

Zhao, Z. and Ding, S., 1976. Discovery of the dinosaurian eggshells from Alxa, Ningxia and its stratigraphic significance. Vertebrata PalAsiatica 14: 42-44.

Zhao, Z. and Jiang, Y., 1974. Microscopic studies on the dinosaurian egg-shells from Laiyang, Shantung Province. Scientia Sinica 17: 73-83.

Zhao, Z. and Li, R., 1988. A new structural type of the dinosaur eggs from Anlu Country, Hubei Province. Vertebrata PalAsiatica 26: 107-115.

Zhao, Z. and Li, R., 1993. First record of Late Cretaceous hypsilophodontid eggs from Bayan Manduhu, Inner Mongolia. Vertebrata Palasiatica 31: 77-84.

Zhao, Z., Zhang, S., Wang, Q. and Wang, X.L., 2013. Dinosaur diversity during the transition between the middle and late parts of the Late Cretaceous in eastern Shangdong Province, China: evidence from dinosaur eggshells. Chinese Science Bulletin 58: 4663-4669.

Zhao, Z.K., 1975. Microstructures of dinosaurian eggshells of Nanxiong, Guangdong, and the problem in egg classification. Vertebrata Palasiatica 13: 105-117.

Zhao, Z.K., Mao, X.Y., Chai, Z.F., Yang, G.C., Kong, P., Ebihara, M. and Zhao, Z.H., 2002. A possible causal relationship between extinction of dinosaurs and K/T iridium enrichment in the Nanxiong Basin, South China: evidence from dinosaur eggshells. Palaeogeography Palaeoclimatology Palaeoecology 178: 1-17.

Zhou, X., Ren, Y., Xu, S. and Guan, K., 1998. Dinosaur eggs of the Late Cretaceous from the Qinglongshan region, Yunxian County, Hubei Province. Hubei Geology and Mineral Resources 12: 1-8.

Zou, S.L., Wang, Q. and Wang, X.L., 2013. A new oospecies of parafaveoloolithids from the Pingxiang Basin, Jiangxi Province of China. Vertebrata PalAsiatica 51: 102-106.
